# Photochemical approach to functionalized benzobicyclo[3.2.1]octene structures via fused oxazoline derivatives from 4- and 5-(*o*-vinylstyryl)oxazoles

**DOI:** 10.3762/bjoc.10.230

**Published:** 2014-09-18

**Authors:** Ivana Šagud, Simona Božić, Željko Marinić, Marija Šindler-Kulyk

**Affiliations:** 1 Department of Organic Chemistry, Faculty of Chemical Engineering and Technology, University of Zagreb, Marulićev trg 19, 10000 Zagreb, Croatia; 2 NMR Center, Rudjer Bošković Institute, Bijenička cesta 54, 10000 Zagreb, Croatia

**Keywords:** bicyclo[3.2.1]octane, intramolecular photocycloaddition, oxazole, styryl, vinyl

## Abstract

Novel *cis*/*trans*-4- and *cis*/*trans*-5-(2-vinylstyryl)oxazoles have been synthesized by Wittig reactions from the diphosphonium salt of α,α’-*o*-xylene dibromide, formaldehyde and 4- and 5-oxazolecarbaldehydes, respectively. In contrast, *trans*-5-(2-vinylstyryl)oxazole has been synthesized by the van Leusen reaction from *trans*-3-(2-vinylphenyl)acrylaldehyde which is prepared from *o*-vinylbenzaldehyde and (formylmethylene)triphenylphosphorane. The 4- and 5-(2-vinylstyryl)oxazoles afford, by photochemical intramolecular cycloaddition, diverse fused oxazoline-benzobicyclo[3.2.1]octadienes, which are identified and characterized by spectroscopic methods. The photoproducts formed are relatively unstable and spontaneously or on silica gel undergo oxazoline ring opening followed by formation of formiato- or formamido-benzobicyclo[3.2.1]octenone derivatives. On irradiation of 4-(2-vinylstyryl)oxazole small quantities of electrocyclization product, 4-(1,2-dihydronaphthalen-2-yl)oxazole, are isolated and spectroscopically characterized.

## Introduction

The bicyclo[3.2.1]octane skeleton is the basic framework of numerous important biologically active natural compounds or their metabolites [1]. Properly functionalized bicyclo[3.2.1]octanes have proved as useful reactive intermediates in stereoselective transformations making these derivatives powerful building blocks in organic synthetic strategies [2]. Various methodologies and new synthetic approaches for their preparation and reactivity have been reviewed [3]. Continuing our long-standing interest for photochemical intramolecular cycloaddition reactions of various β-heteroaryl-*o*-divinylbenzenes, furans [4–6], thiophenes [6–8], pyroles [9–10] and sydnones [11–13], as routes to polycyclic compounds, we turned our attention to oxazole derivatives. The oxazole structure is commonly found in natural products and pharmaceuticals [14–17] and is applied in useful reagents and intermediates in organic synthesis [18–25]. There are examples of oxazole photochemical intermolecular cycloadditions [26–32], but to the best of our knowledge, there are no examples of intramolecular photocycloaddition. We describe herein, the synthesis of new 4- and 5-(2-vinylstyryl)oxazoles (**1**, **2**) and their intramolecular photocycloaddition to diverse fused tetracyclic oxazoline compounds which further spontaneously or during the work-up procedure hydrolyze to benzobicyclo[3.2.1]octenone derivatives. This is a new method for the synthesis of functionalized benzobicyclo[3.2.1]octenes.

## Results and Discussion

**Scheme 1:**
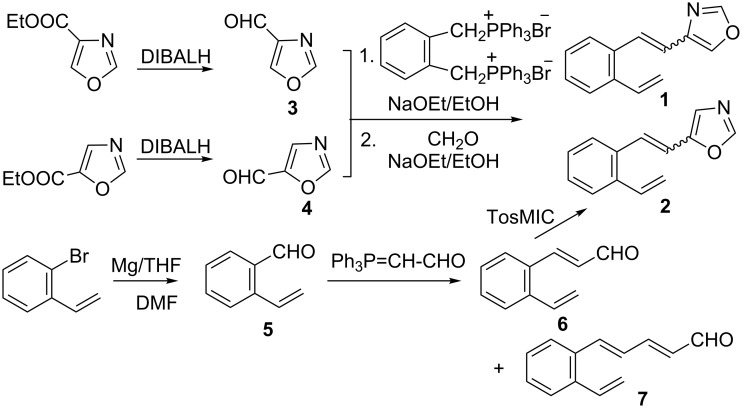
Synthesis of 4- (**1**) and 5-(2-vinylstyryl)oxazoles (**2**).

c*is/trans*-Isomers of 4- and 5-oxazole derivatives (**1**, **2**) were synthesized by Wittig reactions from the diphosphonium salt of α,α’-*o*-xylene dibromide, formaldehyde and oxazole-4- and 5-carbaldehydes (**3**, **4**), respectively, in absolute ethanol with sodium ethoxide as a base (Scheme 1).

The procedure of this multicomponent reaction is slightly modified, compared to the described method [33], in order to optimize the yields. The yield of 4-oxazole derivative **1** was 50% whereas the best result found for the 5-oxazole derivative **2** was 22%. The required oxazole-4/5-carbaldehydes (**3**, **4**) [34–36] were prepared from commercially available esters by DIBALH reduction following the procedure [34] for oxazole-4-carbaldehyde (**3**). The crude products obtained were used in the next step of the synthesis without purification because of their volatility. Reduction of ethyl oxazole-4-carboxylate proceeds completely whereas the crude reaction sample of the oxazole-5-carbaldehyde (**4**) contains 10% of unreacted ester. The unreacted ester is difficult to separate by column chromatography from the *trans*-isomer **2**. It could be removed from the reaction mixture by mild basic hydrolysis [37].

To avoid the use of volatile oxazole-5-carbaldehyde (**4**) we developed a new synthetic route to 5-(2-vinylstyryl)oxazole (**2**) in which the oxazole ring is formed at the end of the reaction sequence (Scheme 1). An oxazole ring substituted in the 5-position can be synthesized from the corresponding aldehydes using van Leusen’s reagent, tosyl methyl isocyanide (TosMIC) [38–39]. For the preparation of 5-(2-vinylstyryl)oxazole (**2**) by this method 3-(2-vinylphenyl)acrylaldehyde (**6**) was needed. This new *o*-substituted phenylacrylaldehyde **6** was prepared using (formylmethylene)triphenylphosphorane by a Wittig reaction from *o*-vinylbenzaldehyde (**5**). The yield of desired product **6** is lower, compared to the yields of previously prepared β-heteroarylacrylaldehydes [23]. This can be explained by the diminished nucleophilic attack of the reagent to the carbonyl moiety due to the steric hindrance of the *o*-vinyl group in **5** and continued competitive reaction of the carbonyl from the formed *o*-vinylphenylacrylaldehyde **6** with (formylmethylene)triphenylphosphorane to give 5-(2-vinylphenyl)penta-2,4-dienal (**7**) as byproduct. Under optimal reaction conditions (see Supporting Information File 1) 32% *trans*-3-(2-vinylphenyl)prop-2-enal (**6**) is obtained in addition with 5% *trans*,*trans*-5-(2-vinylphenyl)penta-2,4-dienal (**7**) as a contaminant, alongside with a large amount of resinous material. The required *o*-vinylbenzaldehyde (**5**) [40] was synthesized from 2-bromostyrene and used without purification. As the starting aldehyde **6** for the reaction with TosMIC was in *trans* configuration the 5-(2-vinylstyryl)oxazole (**2**) obtained retained the *trans* configuration. This is clearly seen from the coupling constants of the ethylene protons (*J* = 16 Hz) in the ^1^H NMR. All new compounds for further experiments, *cis*/*trans*-**1**, *cis*/*trans*-**2** and *trans*-**6** are isolated by column chromatography on silica gel in moderate yields (22–50%) and characterized by spectroscopic methods (see Supporting Information File 1).

**Scheme 2:**
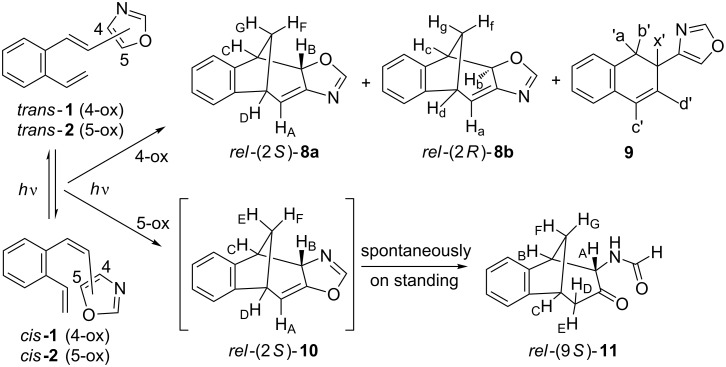
Irradiation of 4- (**1**) and 5-(2-vinylstyryl)oxazoles (**2**) (crude reaction mixtures).

The irradiation experiments have been performed in a Rayonet reactor (>300 nm, rt, using up to 16 lamps each with a power of 8 W) with petroleum ether, acetonitrile or benzene as a solvent. Benzene gave the cleanest reaction profile and was used as the solvent in further preparative experiments. The ^1^H NMR spectra of the crude photomixtures showed complete conversion after 3–5 hours of irradiation. In the case of *cis*/*trans*-4-(2-vinylstyryl)oxazoles **1** two dominant products **8a** (74%) and **8b** (20%) were observed in the ^1^H NMR spectra and a small quantity of **9** (6%) was observed as well (Scheme 2).

**Figure 1:**
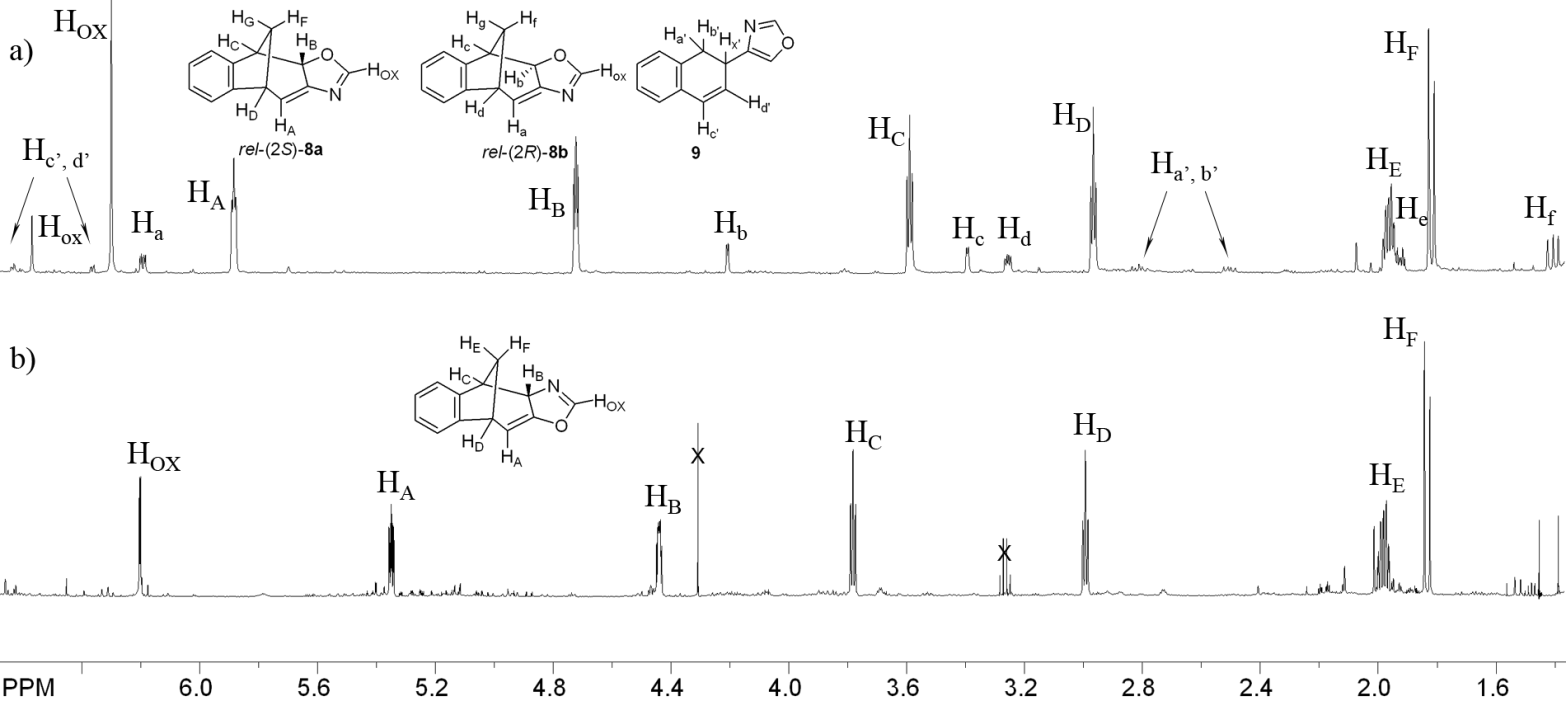
Part of ^1^H NMR spectra in C_6_D_6_ of the crude photomixtures after 200 min (300 nm, rt ) of irradiation of *cis*/*trans*-**1** (a) and *cis*/*trans*-**2** (b).

Whereas in the case of *cis*/*trans*-5-(2-vinylstyryl)oxazoles (**2**) one major product **10** (75%) and minor product **11** (25%) along with a lot of small quantities of unidentified side products (Scheme 2) were observed. Irradiation of compounds **1** and **2** in NMR tubes dissolved in deuterated benzene and recording the spectra at timed intervals demonstrated that **11** is not the photochemical product as can be clearly seen in Figure 1.

After complete conversion of the starting compound **2** only compound **10** was present in the ^1^H NMR spectrum in contrast to photochemical conversion of compound **1** in which the same mixture of three products (**8a**, **8b** and **9**) is obtained as in preparative experiments.

**Scheme 3:**
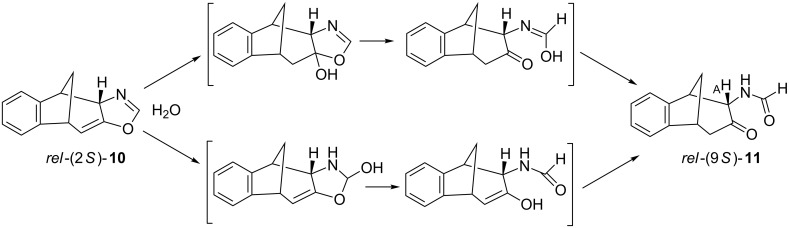
Plausible mechanisms of oxazoline ring-opening in photoproduct **10**.

The structure of photoproduct **10** was completely determined using COSY, NOESY and HSQC techniques (see Supporting Information File 1). Aromatic protons of **10** are at 6.9–7.3 ppm and the proton on the oxazoline moiety is a singlet at 6.2 ppm. The specific aliphatic protons H_A_–H_F_ of the bicyclic skeleton (Figure 1) show a similar pattern as the previously described benzofuran intermediate [41]. In the ^13^C NMR spectrum there are 5 signals in the region from 108 to 40 ppm. The doublet at 108 ppm indicates the structure with sp^2^-hybridized carbon (C_H(A)_) and the triplet at 44 ppm indicates the existence of one geminal carbon atom. The tetracyclic oxazoline stereoisomer *rel*-(2*S*)-**10** undergoes spontaneously oxazoline ring-opening to **11** during the solvent evaporation after the irradiation and therefore the identification of this compound had to be done immediately after the work-up procedure. The formation of tricyclic formamido derivative *rel*-(9*S*)-**11** can be explained by the addition of water to the *exo*-double bond of the bicyclic skeleton or the C=N double bond and further oxazoline ring-opening (Scheme 3).

**Figure 2:**
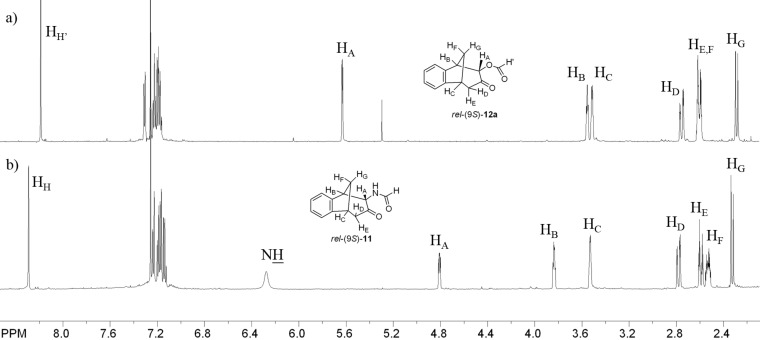
^1^H NMR spectra in C_6_D_6_ of *rel*-(9*S*)-**12a** (a) and *rel*-(9*S*)-**11** (b).

Formamido derivative *rel*-(9*S*)-**11** is completely characterized by spectroscopic methods. In its IR spectrum there are signals at 3334 cm^−1^ of the NH group, and strong signals at 1722 and 1683 cm^−1^ of two carbonyl groups. The formamido proton in the ^1^H NMR spectrum appears at 8.29 ppm and the proton of the NH group as a broad singlet at 6.29 ppm. Specific signals for the aliphatic protons H_A_–H_G_ of the benzobicyclo[3.2.1]octenone structure are present at higher field from 4.8 to 2.3 ppm as expected (Figure 2: (b)). From the NOESY spectrum was evident that the H_A_ is oriented towards the methano bridge.

The diastereomeric fused tetracyclic oxazolines *rel*-(2*S*)-**8a** and *rel*-(2*R*)-**8b** (Scheme 2, Figure 1), that were obtained on irradiation of *cis*/*trans*-4-(2-vinylstyryl)oxazole (**1**), are more stable than **10** but not sufficiently so that they can be separated chromatographically on silica gel. The major diastereomer **8a** is isolated mixing the diastereomeric mixture for several hours in dry ether with some quantity of silica gel. The minor diastereomer **8b** is identified and characterized in the NMR spectra of the photomixtures. The difference in structures of **8a** and **8b** is in the orientation of H_B_ (**8a**) or H_b_ (**8b**) protons. In the NOESY spectrum of the diastereomeric mixture the interaction between H_B_ and H_F_ protons can be seen which is a clear proof that the H_B_ is facing the methano bridge in the major diastereomer **8a**. There is no interaction between H_b_ and H_f_ protons in the minor isomer **8b** suggesting that H_b_ proton is opposite to the methano-bridge. The diastereomer in which the H_B_ is oriented towards the methano bridge is the main product in photochemical reactions of either 4- or 5-(2-vinylstyryl)oxazole.

**Scheme 4:**
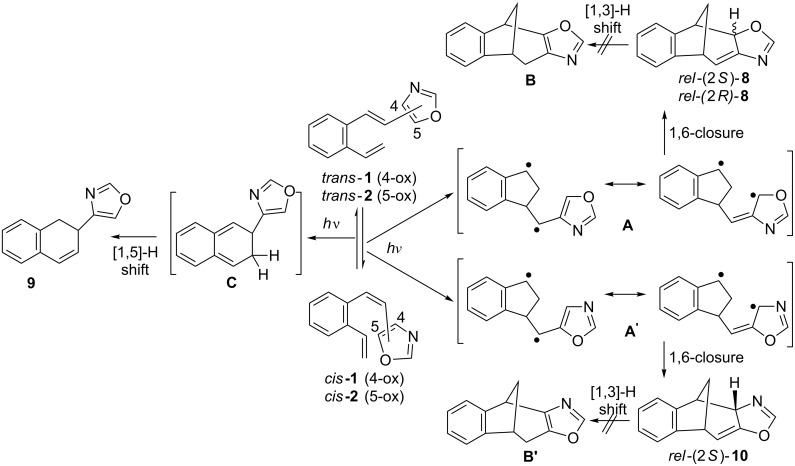
Mechanism of the formation of polycyclic compounds (**8**–**10**).

The formation of the photoproducts **8** and **10** can be explained by intramolecular cycloaddition and formation of resonance stabilized biradicals **A**/**A’** followed by the 1,6-ring closure (Scheme 4).

An 1,3-H shift, as in furan and thiophene derivatives [6], and rearomatization to fused oxazole derivatives **B**/**B’** is not detected. The 1,6-ring closure of the biradicals **A**/**A’** occurs stereoselectively giving the major products *rel*-(2*S*)-**8a**/*rel*-(2*S*)-**10** in which the hydrogen on C-2 is oriented toward the methano bridge. The formation of dihydronaphthalene derivative **9**, found only on irradiation of **1**, is explained by 6π electrocyclization of the benzodivinyl moiety to intermediate **C** followed by 1,5-H shift and rearomatization of the benzene ring. Analogue electrocyclization was not detected in phenyl or furyl-substituted *o*-divinylbenzenes but instead stilbene-like 6π electrocyclization and formation of 1-vinylphenanthrene [42] or 6-vinylnaphtho[2,1-*b*]thiophene [6] occured, respectively.

**Scheme 5:**
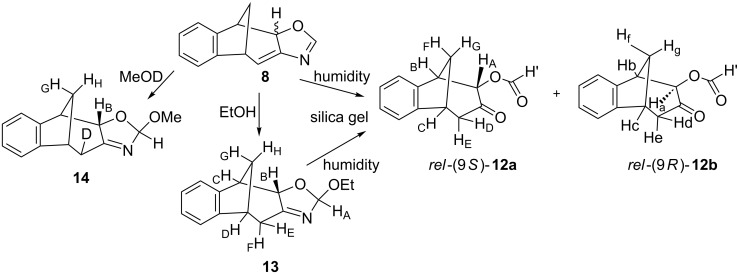
Reactions of the photochemical product **8** with EtOH, MeOD and H_2_O/silica gel.

During chromatography on silica gel the tricyclic formiato derivative *rel*-(9*S*)-**12** is formed from **8** (Scheme 5).

In the ^1^H NMR spectrum the main diastereomer *rel*-(9*S*)-**12a** shows the signal of the formiato proton in the low field at 8.2 ppm. The signals from 5.6 to 2.3 ppm are assigned to H_A_–H_G_ protons of the bicyclic skeleton. Using COSY and NOESY techniques all corresponding interactions are found. The signals of two carbonyl groups located at 200.8 (C=O) and 159.3 (CH'=O) ppm in the ^13^C NMR spectrum were confirmed in the IR spectrum with the bands at 1740 and 1714 cm^–1^. No signal for a NH proton was found in the ^1^H NMR (Figure 2: (a)) nor the NH band in the IR spectrum. Its HRMS also confirmed the compound without the presence of nitrogen. The NOE interaction between protons H_A_ and H_G_ proved that the H_A_ proton is facing to the methano bridge and that in the open structure *rel*-(9*S*)-**12a** H_A_ retained the same orientation as it had in the closed product **8a**. ^1^H NMR spectra of *rel*-(9*S*)-**12a** and *rel*-(9*R*)-**12b** are similar and have comparable interactions of protons in the COSY spectra. Unlike the NOESY spectrum of *rel*-(9*S*)-**12a** with H_A_ at 5.61 ppm in interaction with H_G_, the H_a_ proton at 5.07 ppm of *rel*-(9*R*)-**12b** has no interaction with H_g_.

The photoproduct **8** is more stable than **10**. The stability can be attributed to the existence of conjugated double bonds present in the structure. The structure of **8** in which the *exo*-double bond is in conjugation with the oxazoline double bond is also confirmed by isolation of adducts **13** and **14** (Scheme 5).

**Scheme 6:**
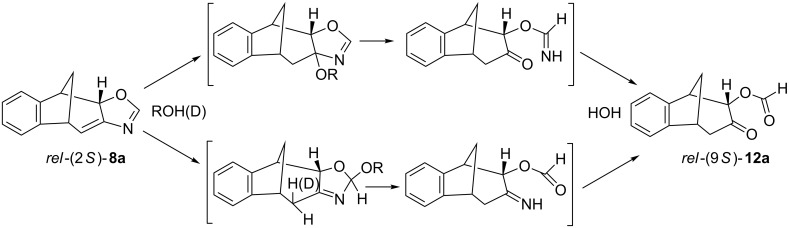
Plausible mechanisms of oxazoline ring opening in photoproduct **10** and formation of **12**.

When the crude photomixture, containing **8a**, **8b** and **9**, was dissolved in absolute ethanol and left in a refrigerator at 13 °C over the weekend the adduct **13** was formed as a main product. The same procedure with MeOD showed the incorporation of deuterium in the bicyclo[3.2.1]octadiene moiety and a methoxy group on the N=C oxazoline bond by 1,4-addition or more likely by addition to the N=C bond followed by keto–enol tautomerization giving **14**. The adducts are spectroscopically completely identified (see Supporting Information File 1). The spectra of alcohol adducts show that alongside with (2*S*)-**13**/**14** there were traces of **9** and traces of what we suspect to be (2*R*)-**13**/**14**. Products **13**/**14** easily undergo ring opening on silica gel giving the same formiato derivative **12**. Plausible mechanism of the ring opening of oxazoline derivative **8** might be as outlined in Scheme 6.

## Conclusion

In summary, photochemical fused oxazoline-benzobicyclo[3.2.1]octadiene products **8** and **10** are formed by photochemical intramolecular cycloaddition of 4- (**1**) and 5-(2-vinylstyryl)oxazoles (**2**), respectively. Product **10** spontaneously undergoes ring opening and formation of benzobicyclo[3.2.1]octenone derivative **11**. Diastereomers **8** are stable under non-acidic conditions allowing the isolation of the main diastereomer **8a**. They are easily hydrolyzed under mildly acidic conditions (silica gel) to functionalized benzobicyclo[3.2.1]octenone derivatives **12**. Related benzobicyclo[3.2.1]octen-3-ones have been prepared by the method of Lansbury from chloroallylindene [43–45], by carbene reaction from benzonorbornadiene [46–47] or by intramolecular insertion of the vinyl group into a carbon–carbon single bond using organometallic catalysts [48]. Herein we have reported a new simple method for the synthesis of functionalized benzobicyclo[3.2.1]octene derivatives using light as a traceless reagent [49].

## Supporting Information

File 1 Experimental part, NMR and IR spectra.
