# Supplementary material for: Photochemical approach to functionalized benzobicyclo[3.2.1]octene structures via fused oxazoline derivatives from 4- and 5-(o-vinylstyryl)oxazoles
Source: Beilstein J Org Chem. 2014 Sep 18;10:2222–9. doi: 10.3762/bjoc.10.230 (PMC4168845; doi:10.3762/bjoc.10.230)

Supporting Information

for

Photochemical approach to functionalized benzobicyclo[3.2.1]octene structures via fused oxazoline derivatives from 4- and 5-(*o*-vinylstyryl)oxazoles

Ivana Šagud[1], Simona Božić[1], Željko Marinić[2] and Marija Šindler-Kulyk[1*]

Address: [1]Department of Organic Chemistry, Faculty of Chemical Engineering and Technology, University of Zagreb, Marulićev trg 19, 10000 Zagreb, Croatia and [2]NMR Center, Rudjer Bošković Institute, Bijenička cesta 54, 10000 Zagreb, *Croatia*

Emai: Marija Šindler-Kulyk* - marija.sindler@fkit.hr

*Corresponding author

Experimental part, NMR and IR spectra

Experimental section 5S

Oxazole-4-carbaldehyde [1] (**3**) ^{1}H and ^{13}C NMR spectra 17S

Oxazole-4-carbaldehyde [1] (**3**) entire range ^{1}H and ^{13}C NMR spectra 18S

Oxazole-5-carbaldehyde [2] (**4**) ^{1}H and ^{13}C NMR spectra 19S

Oxazole-5-carbaldehyde [2] (**4**) entire range ^{1}H and ^{13}C NMR spectra 20S

2-Vinylbenzaldehyde [3] (**5**) ^{1}H NMR spectra 21S

trans-3-(2-Ethenylphenyl)prop-2-enal (**6**) ^{1}H and ^{13}C NMR spectra 22S

trans-3-(2-Ethenylphenyl)prop-2-enal (**6**) entire range ^{1}H and ^{13}C NMR spectra 23S

trans,trans-5-(2-Vinylphenyl)penta-2,4-dienal (**7**) ^{1}H NMR spectra 24S

cis-4-[2-(2-Vinylphenyl)ethenyl]oxazole (*cis*-**1**) ^{1}H and ^{13}C NMR spectra 25S

cis-4-[2-(2-Vinylphenyl)ethenyl]oxazole (*cis*-**1**) entire range ^{1}H and ^{13}C NMR spectra 26S

trans-4-[2-(2-Vinylphenyl)ethenyl]oxazole (*trans*-**1**) ^{1}H NMRspectra in $CDCl_3$, C_6D_6 27S

trans-4-[2-(2-Vinylphenyl)ethenyl]oxazole (*trans*-**1**) ^{13}C NMR spectrum in $CDCl_3$ 28S

trans-4-[2-(2-Vinylphenyl)ethenyl]oxazole (*trans*-**1**) entire range ^{1}H NMR and ^{13}C NMR spectra 29S

cis-5-[2-(2-Vinylphenyl)ethenyl]oxazole (*cis*-**2**) ^{1}H and ^{13}C NMR spectra 30S

cis-5-[2-(2-Vinylphenyl)ethenyl]oxazole (*cis*-**2**) entire range ^{1}H and ^{13}C NMR spectra 31S

trans-5-[2-(2-Vinylphenyl)ethenyl]oxazole (*trans*-**2**) ^{1}H and ^{13}C NMR spectra 32S

trans-5-[2-(2-Vinylphenyl)ethenyl]oxazole (*trans*-**2**) entire range ^{1}H and ^{13}C NMR spectra 33S

rel-(2*S*)-3-oxa-5-azatetracyclo[6.6.1.0^{2,6}.0^{9,14}]pentadeca-4,6,9,11,13-pentaene (**8a**) ^{1}H NMR spectra 34S

Irradiation mixture (**8a**, **8b**, **9**) ^{1}H NMR spectra 35S

Irradiation mixture (**8a**, **8b**) ^{13}C NMR spectra 36S

Irradiation mixture (**8a**, **8b**) COSY and HSQC spectra 37s

Irradiation mixture (**8a**, **8b**) NOESY spectrum 38S

4-(1,2-dihydronaphthalen-2-yl)oxazole (**9**) ^{1}H and ^{13}C NMR spectra 39S

Irradiation mixture (**10**, **11**) ^{1}H NMR spectra in $CDCl_3$, C_6D_6 40S

Irradiation mixture (**10**, **11**) ^{13}C NMR spectra in $CDCl_3$, C_6D_6 41S

Irradiation mixture (**10**, **11**)entire range ^{1}H NMR and ^{13}C NMR spectra in 42S

Irradiation mixture (**10**, **11**) COSY spectrum 43S

Irradiation mixture (**10**, **11**) NOESY spectrum 44S

N-[*rel*-(9*S*)-10-Oxotricyclo[6.3.1.0^{2,7}]dodeca-2,4,6-trien-9-yl]formamide (**11**) ^{1}H and ^{13}C NMR spectra 45S

N-[*rel*-(9*S*)-10-Oxotricyclo[6.3.1.$0^{2,7}$]dodeca-2,4,6-trien-9-yl]formamide (**11**) ^{1}H and ^{13}C NMR

entire range spectra 46S

N-[*rel*-(9*S*)-10-Oxotricyclo[6.3.1.$0^{2,7}$]dodeca-2,4,6-trien-9-yl]formamide (**11**) COSY, HSQC spectra 47S

N-[*rel*-(9*S*)-10-Oxotricyclo[6.3.1.$0^{2,7}$]dodeca-2,4,6-trien-9-yl]formamide (**11**) NOESY spectrum 48S

N-[*rel*-(9*S*)-10-Oxotricyclo[6.3.1.$0^{2,7}$]dodeca-2,4,6-trien-9-yl]formamide (**11**) IR spectrum 49S

rel-(9*S*)-10-Oxotricyclo[6.3.1.$0^{2,7}$]dodeca-2,4,6-trien-9-yl formiate (**12a**) ^{1}H and ^{13}C NMR spectra 50S

rel-(9*S*)-10-Oxotricyclo[6.3.1.$0^{2,7}$]dodeca-2,4,6-trien-9-yl formiate (**12a**) ^{1}H and ^{13}C NMR

entire range spectra 51S

rel-(9*S*)-10-Oxotricyclo[6.3.1.$0^{2,7}$]dodeca-2,4,6-trien-9-yl formiate (**12a**) COSY, HSQC spectra 52S

rel-(9*S*)-10-Oxotricyclo[6.3.1.$0^{2,7}$]dodeca-2,4,6-trien-9-yl formiate (**12a**) NOESY spectrum 53S

rel-(9*S*)-10-Oxotricyclo[6.3.1.$0^{2,7}$]dodeca-2,4,6-trien-9-yl formiate (**12a**) IR spectrum 54S

rel-(9*R*)-10-Oxotricyclo[6.3.1.$0^{2,7}$]dodeca-2,4,6-trien-9-yl formiate (**12b**) ^{1}H and ^{13}C NMR spectra 55S

rel-(9*R*)-10-Oxotricyclo[6.3.1.$0^{2,7}$]dodeca-2,4,6-trien-9-yl formiate (**12b**) ^{1}H and ^{13}C NMR

entire range spectra 56S

rel-(9*R*)-10-Oxotricyclo[6.3.1.$0^{2,7}$]dodeca-2,4,6-trien-9-yl formiate (**12b**) COSY, HSQC spectra 57S

rel-(9*R*)-10-Oxotricyclo[6.3.1.$0^{2,7}$]dodeca-2,4,6-trien-9-yl formiate (**12b**) NOESY spectrum 58S

rel-(2*S*)-4-Ethoxy-3-oxa-5-azatetracyclo[6.6.1.$0^{2,6}$.$0^{9,14}$]pentadeca-5,9,11,13-tetraen (**13**)

^{1}H and ^{13}C NMR spectra 59S

rel-(2*S*)-4-Ethoxy-3-oxa-5-azatetracyclo[6.6.1.$0^{2,6}$.$0^{9,14}$]pentadeca-5,9,11,13-tetraen (**13**)

entire range ^{1}H and ^{13}C NMR spectra 60S

rel-(2*S*)-4-Ethoxy-3-oxa-5-azatetracyclo[6.6.1.$0^{2,6}$.$0^{9,14}$]pentadeca-5,9,11,13-tetraen (**13**)

COSY, HSQC spectra 61S

rel-(2*S*)-4-Ethoxy-3-oxa-5-azatetracyclo[6.6.1.$0^{2,6}$.$0^{9,14}$]pentadeca-5,9,11,13-tetraen (**13**)

NOESY spectrum 62S

rel-(2*S*)-4-Methoxy(7-$^{2}H_{1}$)-3-oxa-5-azatetracyclo[6.6.1.$0^{2,6}$.$0^{9,14}$]pentadeca-5,9,11,13-tetraen (**14**)

^{1}H and ^{13}C NMR spectra 63S

rel-(2*S*)-4-Methoxy(7-$^{2}H_{1}$)-3-oxa-5-azatetracyclo[6.6.1.$0^{2,6}$.$0^{9,14}$]pentadeca-5,9,11,13-tetraen (**14**)

entire range ^{1}H and ^{13}C NMR spectra 64S

rel-(2*S*)-4-Methoxy(7-$^{2}H_{1}$)-3-oxa-5-azatetracyclo[6.6.1.$0^{2,6}$.$0^{9,14}$]pentadeca-5,9,11,13-tetraen (**14**)

COSY, HSQC spectra 65S

rel-(2*S*)-4-Methoxy(7-$^{2}H_{1}$)-3-oxa-5-azatetracyclo[6.6.1.$0^{2,6}$.$0^{9,14}$]pentadeca-5,9,11,13-tetraen (**14**)

NOESY spectrum 66S

EXPERIMENTAL SECTION

General procedures. Reactions which required the use of anhydrous, inert atmosphere techniques were carried out under an atmosphere of nitrogen. Petroleum ether, bp 40–60 °C, was used. Solvents were purified by distillation. Column chromatography was carried out on columns with silica gel (Fluka 0,063-0,2 nm and Fluka 60 Å, technical grade). TLC was carried out using plates coated with silica gel (0,2 mm, 0,5 mm, 1,0 mm, Kiselgel 60 F_{254}). Organic layers were routinely dried with anhydrous $MgSO_4$ and evaporated using a rotary evaporator. ^1H and ^{13}C NMR spectra were recorded on a spectrometer at 300 and 600 MHz. All NMR spectra were measured in $CDCl_3$ using tetramethylsilane as reference and some in C_6D_6. The assignment of the signals is based on 2D-CH correlation and 2D-HH-COSY experiments. The following abbreviations are used: s, singlet; d, doublet; t, triplet; q, quartet, dd, doublet of doublets; m, multiplet and br, broad. UV spectra were measured on a UV/VIS spectrophotometer. IR spectra were recorded on a FTIR-ATR and FTIR. Mass spectra were obtained on a GC-MS system. Melting points were obtained using a microscope equipped apparatus and are uncorrected. HRMS analysis were carried out on a mass spectrometer (MALDI TOF/TOF analyzer), equipped with Nd:YAG laser operating at 355 nm with firing rate 200 Hz in the positive (H+) or negative (−H) ion reflector mode and on a EXTREL FTMS 2001 DD. Irradiation experiments were performed in a tightly closed quartz vessel in benzene solution in a photochemical reactor equipped with 3000 Å lamps. The solvent was removed on the rotatory evaporator under reduced pressure in a ventilated hood.

Synthesis of 4/5-[2-(2-vinylphenyl)ethenyl]oxazoles (1, 2)

Oxazole-4-carbaldehyde [1] (**3**). Yield on crude product: 0.56 g (81%; lit [1] 64%) as colorless solid; ^{1}H NMR ($CDCl_3$, 600 MHz) δ 10.01 (s, 1H, C$\underline{H}$O), 8.32 (brs, 1H, H-2), 7.98 (s, 1H, H-5); ^{13}C NMR ($CDCl_3$, 75 MHz) δ 184.2 (d, $\underline{C}$HO), 151.8 (d, C-2), 143.7 (d, C-5), 140.4 (s).

Oxazole-5-carbaldehyde [2] (**4**). Yield on crude product: 0.73 g (**4** : ester = 6:1 by ^{1}H NMR; lit.[2] 56%) as oil; ^{1}H NMR (600 MHz, $CDCl_3$) δ 9.87 (d, $J_{H,CHO}$ = 0.5 Hz, 1H, C$\underline{H}$O), 8.11 (brs, $J_{H,CHO}$ = 0.5 Hz, 1H, H-4), 7.89 (s, 1H, H-2); ^{13}C NMR (150 MHz, $CDCl_3$) δ 176.9 (d, $\underline{C}$HO), 154.5 (d, C-2), 150.0 (s, C-5), 136.0 (d, C-4).

2-Vinylbenzaldehyde [3] (**5**). Yield on crude product: 0.62 g (90%; lit [3] 66%) as colorless oil; ^{1}H NMR (600 MHz $CDCl_3$) δ 10.30 (s, 1H, C$\underline{H}$O), 7.84 (d, J_{ar} = 7.8 Hz, 1H, H-ar), 7.59-7.57 (m, 2H, H-ar), 7.54 (dd, $J_{c,a}$ = 17.5 Hz, $J_{c,b}$= 11.0 Hz, 1H, H-c), 7.45-7.43 (m, 1H, H-ar), 5.70 (dd, $J_{a,c}$ = 17.5 Hz, $J_{a,b}$ = 1.02 Hz, 1H, H-a), 5.52 (dd, $J_{b,c}$ = 11.0 Hz, $J_{b,a}$ = 1.0 Hz, 1H, H-b).

trans-3-(2-Ethenylphenyl)prop-2-enal (**6**): 0.50 g (3.8 mmol) of 2-vinylbenzaldehyde and 1.15 g (4.2 mmol) of Ph_3P=CH-CHO was refluxed in 100 ml benzene (dried on Na) in strictly anhydrous conditions under nitrogen for 70 h and then let to cool down to room temperature. After removal of the solvent the residue was worked up with ice-water and extracted with ice cold ether. Organic layers were washed with water and dried over anhydrous $MgSO_4$. Evaporation of solvent under reduced pressure afforded the crude product which was further purified by column chromatography on silica gel using petroleum ether/ether (variable ratio) as eluent affording 0.22 g (37%) of the mixture: *trans*-**6** and *trans,trans*-**7** (6:1 according to ^{1}H NMR). By multiple column and thin layer chromatography products were separated and pure ***trans*-6** was isolated as colorless oil: R_f 0.40 (5%

ether : petroleum ether); UV (EtOH; λ_{max}/nm (ε/dm^3 mol^{-1} cm^{-1})): 279 (3838); IR (evaporated film from $CHCl_3$): 3028, 1672, 1475, 1292, 1124, 970 cm^{-1}; ^{1}H NMR (300 MHz, $CDCl_3$) δ 9.73 (d, $J_{et2,CHO}$= 7.7 Hz, 1H, C<u>H</u>O), 7.85 (d, $J_{et1,et2}$= 15.8 Hz, 1H, H-et1), 7.58 (d, J_{ar} = 7.6 Hz, 1H, H-ar), 7.50 (d, J_{ar} = 7.6 Hz, 1H, H-ar), 7.41(t, J_{ar} = 7.6 Hz, 1H, H-ar), 7.33 (t, J_{ar} = 7.4 Hz, 1H, H-ar), 7.1 (dd, $J_{a,c}$= 17.3 Hz, $J_{b,c}$= 11.0 Hz, 1H, H-c), 6.66 (dd, $J_{et1,et2}$= 15.8 Hz, $J_{et2,CHO}$ = 7.7 Hz, 1H, H-2), 5.66 (dd, $J_{a,c}$= 17.3 Hz, $J_{a,b}$ = 1.1 Hz, 1H, H-a), 5.48 (dd, $J_{b,c}$= 11.0 Hz, $J_{a,b}$ = 1.1 Hz, 1H, H-11); ^{13}C NMR (75 MHz, $CDCl_3$) δ 193.8 (d, <u>C</u>HO), 150.3 (d), 138.5 (s), 133.9 (d), 131.9 (s), 131.0 (d), 130.4 (d), 128.1 (d), 127.5 (d), 127.2 (d), 119.0 (t); HRMS (MALDI TOF/TOF; *m*/*z*): [M + H]$^+$calcd for $C_{11}H_{10}O$ 158.0804, found 158.0800.

trans,trans-5-(2-Vinylphenyl)penta-2,4-dienal (***trans,trans*-7**) was isolated in traces as colorless oil : R_f 0.35 (5% ether : petroleum ether); ^{1}H NMR (600 MHz, $CDCl_3$) δ 9.63 (d, $J_{1,2}$= 7.9 Hz, 1H, C<u>H</u>O), 7.69-7.66 (m, 1H, H-ar), 7.57 (d, J_{ar}= 7.5 Hz, 1H, H-ar), 7.47 (d, J_{ar}= 7.5 Hz, 1H, H-ar), 7.33 (d, $J_{3,4}$= 15.3 Hz, 1H, H-4), 7.36-7.28 (m, 2H, H-ar, c), 7.03 (dd, $J_{3,4}$= 17.5 Hz, $J_{2,3}$= 11.0 Hz, 1H, H-3), 6.92 (dd, $J_{1,2}$= 15.5 Hz, $J_{2,3}$ = 11.0 Hz, 1H, H-2), 6.28 (dd, $J_{1,2}$ = 15.5 Hz, $J_{2,CHO}$ = 7.9 Hz, 1H, H-2), 5.64 (dd, $J_{a,c}$ = 17.3 Hz, $J_{a,b}$= 1.08 Hz, 1H, H-a), 5.43 (dd, $J_{b,c}$= 10.9 Hz, $J_{a,b}$= 1.1 Hz, Hz, H-b).

General procedure for the synthesis of 4/5-[2-(2-vinylphenyl)ethenyl]oxazoles (1, 2) *<u>by Wittig reaction</u>*: To a stirred solution of diphosphonium salt of *α,α'*-*o*-xylenedibromide (4.6 g, 6 mmol) in absolute ethanol (100 ml, kept on 3 Å sieves) simultaneously was added dropwise a solution of oxazole-4/5-carbaldehyde (**3**, **4**) (0.58 g, 6 mmol) in 4 mL of ethanol and half of a solution of sodium ethoxide (0.31 g Na, 13 mmol, 2.2 equiv in 30 mL of ethanol) in strictly anhydrous

conditions under nitrogen. Reaction mixture was left to stir for 20 minutes. Under the stream of dry nitrogen, gaseous formaldehyde (obtained by decomposition of paraformaldehyde taken in excess, 1.0 g) was introduced together with the second quantity of sodium ethoxide that was added dropwise. Reaction mixture was left to stir for 2 more hours. After removal of the solvent the residue was worked up with ice-water, and extracted with benzene (6 × 20 mL). Benzene extracts were dried over anhydrous $MgSO_4$. Evaporation of solvent under reduced pressure afforded the crude product. Column chromatography on silica gel using petroleum ether/ether (variable ratio) as eluent afforded a mixture of *cis* and *trans* isomers, *cis*/*trans*-**1** and *cis*/*trans*-**2**, respectively. The isomers were separated by repeated column chromatography on silica gel. *cis/trans*-4-[2-(2-vinylphenyl)ethenyl]oxazole (**1**): 0.58 g (50%) a mixture of *cis*-**1** : *trans*-**1** = 2:3.

cis-**1**: (0.23 g) colorless solid, mp 39-41 °C; R_f 0.45 (12.5% ether:petroleumether); IR (evaporated film from $CHCl_3$): 3024, 1626, 1513, 1100, 913 cm^{-1}; UV ((EtOH) λ_{max}/nm (ε/$dm^3mol^{-1}cm^{-1}$)): 243 (14002), 290 (Sh 5198); ^{1}H NMR ($CDCl_3$, 600 MHz) δ 7.72 (s, 1H, H-2), 7.60 (brd, 1H, J_{ar} = 7.56 Hz, H-ar), 7.32-7.29 (m, 1H, H-ar), 7.26-7.21 (m, 2H, H-ar), 6.88 (s, 1H, H-5), 6.86 (dd, 1H, $J_{a,c}$ = 17.4 Hz, $J_{b,c}$ = 11.0 Hz, H-c), 6.78 (d, J_{et} = 12.0 Hz, 1H, H-et), 6.58 (d, J_{et} = 12.0 Hz, ,1H H-et), 5.68 (dd, $J_{a,b}$ = 1.0 Hz, $J_{a,c}$ = 17.4 Hz, 1H, H-a), 5.23 (dd, $J_{a,b}$ = 1.0 Hz, $J_{b,c}$ = 11.0 Hz, 1H, H-b); ^{13}C NMR ($CDCl_3$, 150 MHz) δ 149.9 (d, C-2), 136.7 (s), 136.6 (s), 126.2 (d, C-5), 135.6 (s), 134.5 (d, C-c), 130.8 (d, C-et), 128.6 (d, C-Ar), 128.0 (d,C-Ar), 125.5 (d, C-Ar), 121.1 (d, C-et), 115.4 (d, C-et).

trans-**1**: (0.35 g) as oil, R_f 0.5 (12.5% ether:petroleumether); IR (evaporated film from $CHCl_3$): 2019, 1638, 1519, 1063 cm^{-1}; UV ((EtOH) λ_{max}/nm (ε/$dm^3mol^{-1}cm^{-1}$)): 248 (17597), 287 (19858);

^{1}H NMR ($CDCl_3$, 600 MHz) δ 7.87 (s, 1H, H-2), 7.67 (s, 1H, H-5), 7.65 (d, 1H, J_{et} = 15.8 Hz, H-et), 7.53-7.47 (m, 2H, H-ar), 7.27-7.26 (m, 2H, H-ar), 7.13 (dd, 1H, $J_{a,c}$ = 17.3 Hz, $J_{b,c}$ = 11.1 Hz, H-c), 6.82 (d, 1H, J_{et} = 15.8 Hz, H-et), 5.64 (dd, 1H, $J_{a,b}$ = 1.1 Hz, $J_{a,c}$ = 17.3 Hz, H-a), 5.36 (dd, 1H, $J_{a,b}$ = 1.1 Hz, $J_{b,c}$ = 11.1 Hz, H-b); ^{1}H NMR (C_6D_6, 600 MHz) δ 8.01 (d, 1H, J_{et} = 15.8 Hz, H-et), 7.40-7.35 (m, 2H, H-ar), 7.11-7.04 (m, 3H, H-ar, H-c), 7.06 (s, 1H, H-2), 6.94 (s, 1H, H-5), 6.61 (d, 1H, J_{et} = 15.8 Hz, H-et), 5.49 (dd, 1H, $J_{a,b}$ = 1.3 Hz, $J_{a,c}$ = 17.4 Hz, H-a), 5.10 (dd, 1H, $J_{a,b}$ = 1.3 Hz, $J_{b,c}$ = 11.0 Hz, H-b); ^{13}C NMR ($CDCl_3$, 150 MHz) δ 151.2 (d, C-2), 138.9 (s), 136.7 (s), 135.5 (d, C-et), 134.9 (s), 134.9 (d, C-c), 128.6 (d, C-5), 127.9 (d, C-Ar), 127.8 (d, C-Ar), 126.6 (d, C-Ar), 126.1 (d, C-Ar), 118.4 (d, C-et), 116.7 (t, C-a,b); HRMS (MALDI TOF/TOF; *m*/*z*): $[M+H]^+$ calcd for $C_{13}H_{11}NO$ 198.0916, found 198.0913.

cis/trans-5-[2-(2-Vinylphenyl)ethenyl]oxazole (**2**): 0.26 g (22%) as a mixture of *cis*-**2**: *trans*-**2** : ethyl oxazole-5-carboxylate ester = 1.5:7.5:1. Unreacted ester (impurity from the preparation of **4**) was removed by treatment of the mixture with NaOH in water and THF.

cis-**2**: (0.04 g) as yellow oil, R_f 0.30 (23% ether:petroleumether); UV (EtOH; λ_{max}/nm (ε/dm^{3}mol^{-1}cm^{-1})): 249 (14149), 281 (7851); ^{1}H NMR (600 MHz, $CDCl_3$) δ 7.68 (s, 1H, H-2), 7.58 (d, 1H, J_{ar} = 7.8 Hz, H-ar), 7.32-7.30 (m, 1H, H-ar), 7.26-7.24 (m, 2H, H-ar), 7.68 (dd, 1H, $J_{a,c}$ = 17.5 Hz, $J_{b,c}$ = 11.0 Hz, H-c), 6.76 (d, 1H, J_{et} = 12.2 Hz, H-et), 6.59 (s, 1H, H-4), 6.54 (d, 1H, J_{et} = 12.2 Hz, H-et), 5.68 (dd, 1H, $J_{a,b}$ = 1.1 Hz, $J_{a,c}$ = 17.5 Hz, H-a), 5.25 (dd, 1H, $J_{a,b}$ = 1.1 Hz, $J_{b,c}$ = 11.0 Hz, H-b); ^{1}H NMR (600 MHz, C_6D_6) δ 7.40 (dd, 1H, J_{ar} = 7.8, 1.2 Hz, H-ar), 7.10 (ddd, 1H, J_{ar} = 7.6, 1.3, 0.6 Hz, H-ar), 7.04 (tdd, 1H, J_{ar} = 7.6, 1.3, 0.6 Hz, H-ar), 7.02 (s, 1H, H-2), 6.97 (td, 1H, J_{ar} = 7.5, 1.3 Hz, H-ar), 6.78 (dd, 1H, $J_{a,c}$ = 17.5 Hz, $J_{b,c}$ = 11.0 Hz, H-c), 6.64 (s, 1H, H-4), 6.38 (d, 1H, J_{et} = 12.2 Hz, H-et), 6.18 (d, 1H, J_{et} = 12.2 Hz, H-et), 5.52 (dd, 1H, $J_{a,b}$ = 1.2 Hz, $J_{a,c}$ = 17.5 Hz, H-a),

5.06 (dd, $J_{a,b}$ = 1.2 Hz, $J_{b,c}$ = 11.0 Hz, H-b); ^{13}C NMR (150 MHz, $CDCl_3$) δ 150.0 (d, C-2), 149.3 (s, C-5), 136.0 (s, C-ar), 135.6 (s, C-ar), 134.6 (d), 130.6 (d), 128.9 (d), 128.3 (d), 127.9 (d), 125.7 (d), 125.5 (d), 116.8 (d), 115.9 (t, C-a, b); HRMS (MALDI TOF/TOF; *m/z*): $[M + H]^+$ calcd for $C_{13}H_{11}NO$ 198.0912, found 198.0912. *Trans*-**2** was isolated as yellow oil: R_f 0.20 (23% ether:petroleumether).

***trans*-5-[2-(2-Vinylphenyl)ethenyl]oxazole** *by Van Leusen reaction*: 0.10 g (0.6 mmol) of (*E*)-3-(2-vinylphenyl)acrylaldehyde (**6**), 0.12 g (0.6 mmol) of TosMIC and 0.09 g (0.6 mmol) of K_2CO_3 was mixed in methanol and refluxed for 3 h. After removal of the solvent the residue was worked up with ice-water acidified to neutral and extracted with ice cold ether. Organic layers were washed with water and dried over anhydrous $MgSO_4$. Evaporation of solvent under reduced pressure afforded the crude product which was further purified by column chromatography on silica gel using petroleum ether/ether (variable ratio) as eluent.

trans-**2**: (0.07 g, 60%) as yellow oil, R_f 0.20 (23% ether:petroleumether); UV (EtOH; λ_{max}/nm (ε/dm^{3}mol^{-1}cm^{-1})): 252 (13934), 301 (18636), 311 (17444), 326 (9629); ^{1}H NMR (600 MHz, $CDCl_3$) δ 7.84 (s, 1H, H-2), 7.54-7.50 (m, 1H, H-ar), 7.50-7.46 (m, 1H, H-ar), 7.42 (d, 1H, J_{et} = 16.0 Hz, 14 Hz, H-et), 7.31-7.27 (m, 2H, H-ar), 7.08 (dd, 1H, $J_{a,c}$ = 17.3 Hz, $J_{b,c}$ = 11.0 Hz, H-c), 7.08 (s, 1H, H-4), 6.81 (d, 1H, J_{et} = 16.1 Hz, H-et), 5.65 (dd, 1H, $J_{a,b}$ = 1.3 Hz, $J_{a,c}$ = 17.3 Hz, H-a), 5.40 (dd, 1H, $J_{a,b}$ = 1.3 Hz, $J_{b,c}$ = 11.0 Hz, H-b); ^{1}H NMR (600 MHz, C_6D_6) δ 7.39 (d, 1H, J_{et} = 16.1 Hz, H-et), 7.34-7.32 (m, 1H, H-ar), 7.25-7.23 (m, 1H, H-ar), 7.20 (s, 1H, H-2), 7.07-7.03 (m, 2H, H-ar), 6.95 (dd, 1H, $J_{a,c}$ = 17.3 Hz, $J_{b,c}$ = 11.0 Hz, H-c), 6.80 (s, 1H, H-4), 6.42 (d, 1H, J_{et} = 16.1 Hz, H-et), 5.47 (dd, 1H, $J_{a,b}$ = 1.4 Hz, $J_{a,c}$ = 17.3 Hz, H-a), 5.14 (dd, $J_{a,b}$ = 1.4 Hz, $J_{b,c}$ = 11.0 Hz, H-b); ^{13}C NMR (150 MHz, $CDCl_3$) δ 150.7 (s, C-5), 150.5 (d, C-2), 137.0 (s, C-ar), 134.8 (d, C-c),

134.4 (s, C-ar), 128.5 (d, C-ar), 128.1 (d, C-ar), 128.1 (d, C-ar), 127.0 (d, C-et), 126.1 (d, C-ar), 124.4 (d, C-4), 117.4 (t, C-ab), 115.1 (d, C-et); MS *m/z* (%, fragment): 197 (100%, M^+), 141 (9%), 115 (23%).

Irradiation of 4-[2-(2-vinylphenyl)ethenyl]oxazole (1) in benzene. 0.20 g of mixture of *cis*- and *trans*-**1** was dissolved in 340 mL of benzene (c = 3 × 10^{-3} mol/L) (purged with argon for 30 minutes) and irradiated with 16 UV lamps at 300 nm during 5h. After removal of solvent at reduced pressure ^{1}H NMR spectrum of crude product show mixture of products *rel*-(2*S*)-**8a** : *rel*-(2*R*)-**8b** : **9** = 12:3.3:1. Crude product was stirred in dry ether with equimolar quantity of silica gel and *rel-(2S)-3-oxa-5-azatetracyclo[6.6.1.0^{2,6}.0^{9,14}]pentadeca-4,6,9,11,13-pentaene* (*rel*-(2*S*)-**8a**) was isolated (0.13 g, 88.4%) as colorless powder, mp 132-133 °C; IR (evaporated film from $CHCl_3$): 3060, 2925, 1563, 1308, 1057 cm^{-1}; ^{1}H NMR ($CDCl_3$, 600 MHz) δ 7.16 – 7.14 (m, 2H, H-Ar), 7.11 – 7.09 (m, 2H, H-Ar), 6.82 (s, 1H, H-OX), 5.89 (dd, $J_{A,B}$ = 3.6 Hz, $J_{A,D}$ = 4.9 Hz, $J_{A,E}$ = 1.8 Hz, 1H, H–A), 5.18 (dd, $J_{A,B}$ = 3.6 Hz, $J_{B,C}$ = 5.1 Hz, 1H, H–B), 4.06 (t, $J_{B,C}$ = $J_{C,E}$ = 5.1 Hz, 1H, H–C), 3.44 (t, $J_{A,D}$ = $J_{D,E}$ = 4.9 Hz, 1H, H–D), 2.49 – 2.45 (m, 1H, H–E), 2.34 (d, $J_{E,F}$ = 11.3, 1H, H–F); ^{13}C NMR ($CDCl_3$, 150 MHz) δ 158.7 (d, C-OX), 153.1 (s), 144.2 (s), 138.2 (s), 127.1 (d, C-Ar), 126.6 (d, C-Ar), 126.4 (d, C-Ar), 120.9 (d, C-Ar), 120.0 (d, C-A), 79.7 (d, C-B), 44.1 (d, C-C), 43.7 (t, C-E,F), 40.9 (d, C-D); MS *m/z* (EI): 197 (100, M^+); HRMS (MALDI TOF/TOF; *m/z*): $[M + H]^+$ calcd for $C_{13}H_{11}NO$ 198.0905, found 198.0913.

rel-(2R)-3-Oxa-5-azatetracyclo[6.6.1.0^{2,6}.0^{9,14}]pentadeca-4,6,9,11,13-pentaene (*rel*-(2*R*)-**8b**) (Not isolated because of its small quantity and instability. ^{1}H and ^{13}C NMR assigned from a mixture of diastereomers): ^{1}H NMR ($CDCl_3$, 600 MHz) δ 7.26-7.24 (m, 2H, H-ar), 7.18-7.17 (m, 2H, H-ar), 7.06 (s, 1H, H-ox), 6.26 (dd, $J_{a,b}$ = 3.2 Hz, $J_{a,c}$ = 7.8 Hz, 1H, H-a), 4.40 (dd, $J_{a,b}$ = 3.2 Hz, $J_{b,c}$ = 1.5

Hz, 1H, H-b), 3.75 (dd, $J_{a,c}$ = 7.8 Hz, $J_{c,e}$ = 4.4 Hz, 1H, H-c), 3.71 (brd $J_{b,d}$ = 1.5 Hz, $J_{d,e}$ = 3.0 Hz, 1H, H-d), 2.32-2.29 (m, 1H, H-e), 1.62 (d, $J_{e,f}$ = 10.5 Hz, 1H, H-f); ^{13}C NMR ($CDCl_3$, 150 MHz) δ 158.1 (d, C-ox), 148.3 (s), 147.4 (s), 144.4 (s), 128.3 (d, C-ar), 127.3 (d, C-ar), 126.8 (d, C-ar), 123.5 (d, C-ar), 121.5 (d, C-ar), 79.9 (d, C-b), 41.2 (d, C-c), 40.9 (t, C-e,f), 40.9 (d, C-d).

4-(1,2-Dihydronaphthalen-2-yl)oxazole (**9**) was isolated as oil by repeated thin layer chromatography of crude irradiation mixture: IR (evaporated film from $CHCl_3$): 2917, 2846, 1514, 1459, 1097 cm^{-}1; ^{1}H NMR ($CDCl_3$, 600 MHz) δ 7.86 (s, 1H, H-2'), 7.35 (d, 1H, H-ar'), 7.30 (s, 1H, H-5'), 7.30 (dd, 1H, H-ar'), 7.26 (m, 1H, H-ar'), 7.18 (dd, 1H, H-ar'), 6.81 (dd, $J_{c,d}$ = 5.5 Hz, $J_{c,x}$ = 1.5 Hz, 1H, H-c'), 6.50 (dd, $J_{c,d}$ = 5.5 Hz, $J_{d,x}$ = 1.8 Hz, 1H, H-d'), 3.87-3.83 (m, 1H, H-x'), 3.07 (dd, $J_{a,b}$ = 14.6 Hz, $J_{a,x}$ = 6.2 Hz, 1H, H-a'), 2.74 (dd, $J_{a,b}$ = 14.6 Hz, $J_{b,x}$ = 8.8 Hz, 1H, H-b'); ^{13}C NMR ($CDCl_3$, 150 MHz) δ 150.8 (d, C-2'), 146.8 (s), 144.3 (s), 138.7 (d, C-d'), 138.5 (s), 134.9 (d, C-5'), 131.4 (d, C-c'), 126.8 (d, C-ar'), 124.8 (d, C-ar'), 123.0 (d, C-ar'), 121.2 (d, C-ar'), 40.1 (d, C-x'), 28.1 (t, C-a',b').

Experiments of addition on 8

In moist ether: 0.06 g of raw photochemical mixture (**8a**, **8b** and **9**) in moist ether (30mL) was stirred 24h with addition of some silica gel. Silica gel was filtered out and the rest purified by column chromatography on silica gel with petroleumether/ether (3:1) as eluens and gave 0.005 g (10.4%) of *rel-(9S)-10-oxotricyclo[6.3.1.0^{2,7}]dodeca-2,4,6-trien-9-yl formiate* (*rel*-(9*S*)-**12a**) as oil: R_f 0.2 (PE/E/EtOH = 3:3:1); UV ((EtOH) λ_{max}/nm (ε/dm^{3}mol^{-1}cm^{-1})): 254 (3740), 267 (Sh 2867), 273 (1798); IR (evaporated film from $CHCl_3$): 3024, 2952, 1740 (C=O), 1714 (C=O), 1471, 1167 (C-O-C), 1034 cm^{-1}; ^{1}H NMR ($CDCl_3$, 600 MHz) δ 8.16 (d, $J_{A,H'}$ = 0.8 Hz, 1H, H-H'), 7.30 (d, J_{ar} =

7.0 Hz, 1H, H-ar), 7.21-7.16 (m, 3H, H-ar), 5.61 (dd, $J_{A,B}$ = 3.6 Hz, $J_{A,H'}$ = 0.8 Hz 1H, H-A), 3.53 (dd, $J_{A,B}$ = $J_{B,F}$ = 4.0 Hz, 1H, H-B), 3.48 (dd, $J_{C,D}$ = 3.6 Hz, $J_{C,F}$ = 7.4 Hz, 1H, H-C), 2.72 (dd, $J_{D,E}$ = 15.4 Hz, $J_{C,D}$ = 3.6 Hz, 1H, H-D), 2.59 – 2.55 (m, 2H, H-E,F), 2.33 (d, $J_{F,G}$ = 11.9 Hz, 1H, H-G); ^{13}C NMR ($CDCl_3$, 150 MHz) δ 200.8 (s, C=O), 159.3 (d, <u>C</u>H'O), 144.9 (s), 140.6 (s), 127.8 (d, C-ar), 126.9 (d, C-ar), 124.9 (d, C-ar), 122.7 (d, C-ar), 78.3 (d, C-A), 42.2 (t, C-D,E), 45.3 (d, C-B), 41.6 (t, C-F,G), 40.5 (d, C-C); HRMS (MALDI TOF/TOF; *m/z*): [M + K]$^+$ calcd for $C_{13}H_{12}O_3$ 255.0419, found 255.0414.

By repeated thin layer chromatographies of the raw photochemical mixture *rel-(9R)-10-oxotricyclo[6.3.1.0^{2,7}]dodeca-2,4,6-trien-9-yl formiate* ((9*R*)-**12b**) was isolated as oil: R_f 0.25 (PE/E/EtOH = 3:3:1); ^{1}H NMR ($CDCl_3$, 600 MHz) δ 8.14 (d, $J_{a,H'}$ = 0.8 Hz, H-H'), 7.35-7.30 (m, 2H, H-ar), 7.21-7.20 (m, 2H, H-ar), 5.07 (dd, $J_{a,H'}$ = 0.8 Hz, $J_{a,b}$ = 3.8 Hz, $J_{a,g}$ = 1.5 Hz, 1H, H-a), 3.60 (t, $J_{a,b}$ = $J_{b,g}$ = 3.8 Hz, 1H, H-b), 3.45 (ddd, $J_{c,d}$ = 4.0 Hz, $J_{c,e}$ = 5.3 Hz, $J_{c,g}$ = 2.0 Hz, H-c), 2.88 (dd, $J_{d,e}$ = 17.0 Hz, $J_{c,d}$ = 4.0 Hz, 1H, H-d), 2.56 (dd, $J_{d,e}$ = 17.0 Hz, $J_{c,d}$ = 4.0 Hz, 1H, H-e), 2.42 (d, $J_{f,g}$ = 11.7 Hz, 1H, H-f), 2.35-2.32 (m, 1H, H-g); ^{13}C NMR ($CDCl_3$, 150 MHz) δ 203.5 (s), 158.8 (d, <u>C</u>H'O), 146.8 (s), 139.4 (s), 128.1 (d, C-ar), 127.4 (d, C-ar), 124.5 (d, C-ar), 123.1 (d, C-ar), 75.6 (d, C-a), 46.8 (t, C-d,e), 44.3 (d, C-b), 38.9 (d, C-c), 36.1 (t, C-f,g); HRMS (MALDI TOF/TOF; *m/z*): [M + Na]$^+$ calcd for $C_{13}H_{12}O_3$ 239.0679, found 239.0678.

<u>*In absolute ethanol:*</u> raw mixture of 0.006 g of diastereomers **8** is left in 20 mL of dry ethanol (sieves 3Å) over the weekend in the refrigerator. The solvent was removed and the product was spectroscopically indentified as *rel-(2S)-4-ethoxy-3-oxa-5-azatetracyclo[6.6.1.0^{2,6}.0^{9,14}]pentadeca-5,9,11,13-tetraen* (*rel*-(2*S*)-**13**): oil; IR (evaporated film from $CHCl_3$) 2926, 1664, 1471, 1099, 1071

cm^{-1}; ^{1}H NMR ($CDCl_3$, 600 MHz) δ 7.21 (d, J_{ar} = 7.2 Hz, 1H, H-ar), 7.17-7.15 (m, 2H, H-ar), 7.14-7.10 (m, 1H, H-ar), 5.85 (d, $J_{A,B}$ = 3.8 Hz, H-A), 4.90 (dd, $J_{A,B}$ = 3.8 Hz, $J_{B,C}$ = 4.0 Hz, 1H, H-B), 3.67 (dq, $J_{CHH,CH3}$ = 7.0 Hz, J_{CHH} = 9.0 Hz, 1H, OC$\underline{H}$H), 3.57 (dq, $J_{CHH,CH3}$ = 7.0 Hz, J_{CHH} = 9.0 Hz, 1H, OCH$\underline{H}$), 3.53 (dd, $J_{B,C}$ = 3.9 Hz, $J_{C,G}$ = 6.3 Hz, H-C), 3.48 (dd, $J_{D,E}$= 3.3 Hz, $J_{D,G}$ = 8.7 Hz, H-D), 2.76 (dd, $J_{D,E}$ = 3.3 Hz, $J_{E,F}$ = 14.0 Hz, H-E), 2.71 (dt, $J_{F,G}$ = $J_{D,F}$ = 3.5 Hz, $J_{E,F}$ = 14.0 Hz, H-F), 2.46-2.41 (m, 1H, H-G), 1.94 (d, $J_{G,H}$= 11.9 Hz, H-H), 1.20 (t, $J_{CHH,CH3}$ = 7.0 Hz, C$\underline{H}_3$); ^{13}C NMR ($CDCl_3$, 150 MHz) δ 176.7 (s, C=N), 144.6 (s), 140,77 (s), 127.3 (d, C-ar), 127.5 (d, C-ar), 125.7 (d, C-ar), 122.7 (d, C-ar), 119.1 (d, C-A), 84.5 (d, C-B), 61.9 (t, $\underline{C}H_2$), 46.2 (d, C-C), 41.1 (d, C-D), 39.9 (t, C-G/H), 37.3 (t, C-E,F), 14.6 (q, $\underline{C}H_3$); MS *m/z* (EI): 244 (100, M^+); HRMS (MALDI TOF/TOF; *m/z*): $[M + H]^+$ calcd for $C_{15}H_{17}NO_2$ 244.1332, found 244.1335.

In deuterated methanol: raw mixture of 0.01 g of diastereomers **8** is left in 3 mL of deuterated methanol (CH_3OD) over the weekend in refrigerator. The solvent was removed and the product was spectroscopically indentified as *rel-(2S)-4-methoxy(7-$^2H_1$)-3-oxa-5-azatetracyclo[6.6.1.0^{2,6}.0^{9,14}]pentadeca-5,9,11,13-tetraen* (*rel*-(2*S*)-**14**): oil, IR (evaporated film from $CHCl_3$): 2928, 1663, 1470, 1073 (C-O-C) cm^{-1}; ^{1}H NMR ($CDCl_3$, 600 MHz) δ 7.21 (brd, J_{ar} = 7.1 Hz, 1H, H-ar), 7.17-7.15 (m, 2H, H-ar), 7.14-7.12 (m, 1H, H-ar), 5.80 (d, $J_{A,B}$ = 4.0 Hz, H-A), 4.89 (t, $J_{A,B}$ = $J_{B,C}$ = 4.0 Hz, 1H, H-B), 3.54 (dd, $J_{B,C}$ = 4.0 Hz, $J_{C,G}$ = 5.5 Hz 1H, H-C), 3.48 (dd, $J_{D,F}$= 4.4 Hz, $J_{D,G}$ = 3.4 Hz, H-D), 3.34 (s, 3H, OC$\underline{H}_3$), 2.70 (brd, $J_{D,F}$ = 4.4 Hz, H-F), 2.46-2.42 (m, 1H, H-G), 1.94 (d, $J_{G,H}$= 12.0 Hz, H-H); ^{13}C NMR ($CDCl_3$, 150 MHz) δ 177.1 (s, C=N), 144.5 (s), 140.7 (s), 127.3 (d, C-ar), 126.6 (d, C-ar), 124.6 (d, C-ar), 122.6 (d, C-ar), 119.8 (d, C-A), 84.8 (d, C-B), 53.0 (q, O$\underline{C}H_3$), 46.3 (d, C-C), 41.1 (d, C-D), 40.0 (t, C-G/H), 37.0 (deutr. E/F); MS *m/z* (EI):

231 (100, M^+); HRMS (MALDI TOF/TOF; *m/z*): $[M + H]^+$ calcd for $C_{14}H_{14}DNO_2$ 231.1254, found 231.1250.

Irraddiation of 5-[2-(2-vinylphenyl)ethenyl]oxazole (2) in NMR tube. *cis*-**2** (c ≈ 0.178 mol/L) was irradiated in an NMR tube in deuterated benzene (purged with argon for 15 minutes) at 300 nm in a photo reactor equipped with 16 UV lamps. *Cis-trans* isomerisation was observed along with formation of the product *rel-(2S)-5-oxa-3-azatetracyclo[6.6.1.0^{2,6}.0^{9,14}]pentadeca-3,6,9,11,13-pentaene* (*rel*-(2S)-**10**): ^{1}H NMR (600 MHz, C_6D_6) δ 7.34-7.32 (m, 1H, H-ar), 6.99 (td, 1H, J_{ar} = 7.3, 1.4 Hz, H-ar), 6.96 (td, 1H, J_{ar} = 7.3, 1.4 Hz, H-ar), 6.96(td, 1H, J_{ar} = 7.3, 1.4 Hz, H-ar), 6.20 (d, 1H, $J_{2,B}$ = 2.3 Hz, H-2), 5.34 (ddd, 1H, $J_{A,B}$ = 3.4 Hz, $J_{A,D}$ = 5.1 Hz, $J_{A,E}$ = 1.3 Hz, H-A), 4.43-4.45 (m, 1H, H-B), 3.78 (t, 1H, $J_{B,C}$ = $J_{C,E}$ = 5.1 Hz, H-C), 2.99 (t, 1H, $J_{A,D}$ = $J_{D,E}$ = 5.1 Hz, H-D), 1.98 (tdd, $J_{A,E}$ = 1.3 Hz, $J_{C,E}$ = $J_{D,E}$ = 5.1 Hz, $J_{E,F}$ = 11.0 Hz, H-E), 1.83 (d, 1H, $J_{E,F}$ = 11.0 Hz, H-F); ^{1}H NMR (600 MHz, $CDCl_3$) δ 7.30-7.28 (m, 1H, H-ar), 7.15-7.13 (m, 1H, H-ar), 7.12-7.10 (m, 2H, H-ar), 6.82 (d, 1H, $J_{2,B}$ = 2.3 Hz, H-2), 5.50 (ddd, 1H, $J_{A,B}$ = 3.4 Hz, $J_{A,D}$ = 5.1 Hz, $J_{A,E}$ = 1.3 Hz, H-A), 4.75-4.73 (m, 1H, H-B), 4.07 (t, 1H, $J_{B,C}$ = $J_{C,E}$ = 5.1 Hz, H-C), 3.45 (t, 1H, $J_{A,D}$ = $J_{D,E}$ = 5.1 Hz, H-D), 2.42 (tdd, $J_{A,E}$ = 1.3 Hz, $J_{C,E}$ = $J_{D,E}$ = 5.1 Hz, $J_{E,F}$ = 11.0 Hz, H-E), 2.30 (d, 1H, $J_{E,F}$ = 11.0 Hz, H-F); ^{13}C NMR (150 MHz, $CDCl_3$) δ 154.3 (d, C-2), 127.7 (d, C-ar), 126.8 (d, C-ar), 126.0 (d, C-ar), 119.9 (d, C-ar), 108.2 (d, C-A), 65.8 (d, C-B), 44.1 (d, C-C), 43.7 (t, C-E,C-F), 39.8 (d, C-D).

Irradiation of 5-[2-(2-vinylphenyl)ethenyl]oxazole (2) in quartz vessel. 0.02 g (6 x 10^{-3} mol/L) of *trans*-**2** in 17 mL of benzene was irradiated at 300 nm in a photoreactor equipped with 12 UV lamps for 3 h. Evaporation of solvent under reduced pressure afforded the crude product which by ^{1}H NMR spectrum is a mixture of *rel*-(2*S*)-**10**: *rel*-(9*S*)-**11**= 3 : 1 which by further purification by column chromatography on silica gel using petroleum ether/ether/ethanol (variable ratio) as eluent

gave *N-[rel-(9S)-10-oxotricyclo[6.3.1.0^{2,7}]dodeca-2,4,6-trien-9-yl]formamide* (*rel*-(9*S*)-**11**) (0.005 g, 23.3%) as oil: R_f 0.05 (petroleum ether /ether/ethanol = 1 :1 : 0.2); UV ((EtOH λ_{max}/nm (ε/dm^{3}mol^{-1}cm^{-1})): 265 (2549), 272 (2467); IR (evaporated film from $CHCl_3$): 3334 (N-H), 2953, 1722 (C=O), 1683 (H-N-C=O), 1505, 1471, 1266 cm^{-1}; ^{1}H NMR (600 MHz, $CDCl_3$) δ 8.29 (s, 1H, C<u>H</u>O), 7.23 (brd, 1H, H-ar), 7.21-7.13 (m, 3H, H-ar), 6.29 (brs, 1H, N<u>H</u>), 4.81 (dd, $J_{A,NH}$ = 5.7 Hz, $J_{A,B}$ = 3.8 Hz, 1H, H-A), 3.84 (dd, $J_{B,F}$ = 7.6 Hz, $J_{A,B}$ = 3.8 Hz, 1H, H-B), 3.54 (m, 1H, H-C), 2.79 (dd, $J_{D,E}$ = 15.1 Hz, $J_{C,D}$ = 3.3 Hz, 1H, H-D), 2.60 (ddd, $J_{D,E}$ = 15.1 Hz, $J_{C/E}$ = 3.4 = $J_{E,F}$ = 3.4 Hz, 1H, H-E), 2.53 (m, 1H, H-F), 2.33 (d, $J_{F,G}$ = 11.9 Hz, 1H, H-G); ^{13}C NMR (150 MHz, $CDCl_3$) δ 204.6 (s, <u>C</u>=O), 160.2 (d, <u>C</u>HO), 145.5 (s), 141.6 (s), 127.7 (d, C-ar), 126.8 (d, C-ar), 124.1 (d, C-ar), 122.9 (d, C-ar), 61.5 (d, C-A), 47.5 (t, C-E/D), 46.0 (d, C-B), 42.0 (t, C-F/G), 41.2 (d, C-C); HRMS (MALDI TOF/TOF; *m/z*) [M + Na]$^+$ calcd for $C_{13}H_{13}NO_2$ 238.0839, found 238.0837.

References

1. Reeves, J. T.; Song, J. J.; Tan, Z.; Lee, H.; Yee, N. K.; Senanayake, C. H. *Org. Lett.* **2007**, *9*, 1875-1878.
2. Mulder, R. J.; Shafer, C. M.; Molinski, T. F. *J. Org. Chem.* **1999**, *64*, 4995-4998.
3. Dale, W. J.; Starr, L.; Strobel, C. W. *J. Org. Chem.* **1961**, *26*, 2225-2227.

Oxazole-4-carbaldehyde[1] (compound 3)

^{1}H NMR spectrum (600 MHz, $CDCl_3$) (**3**)

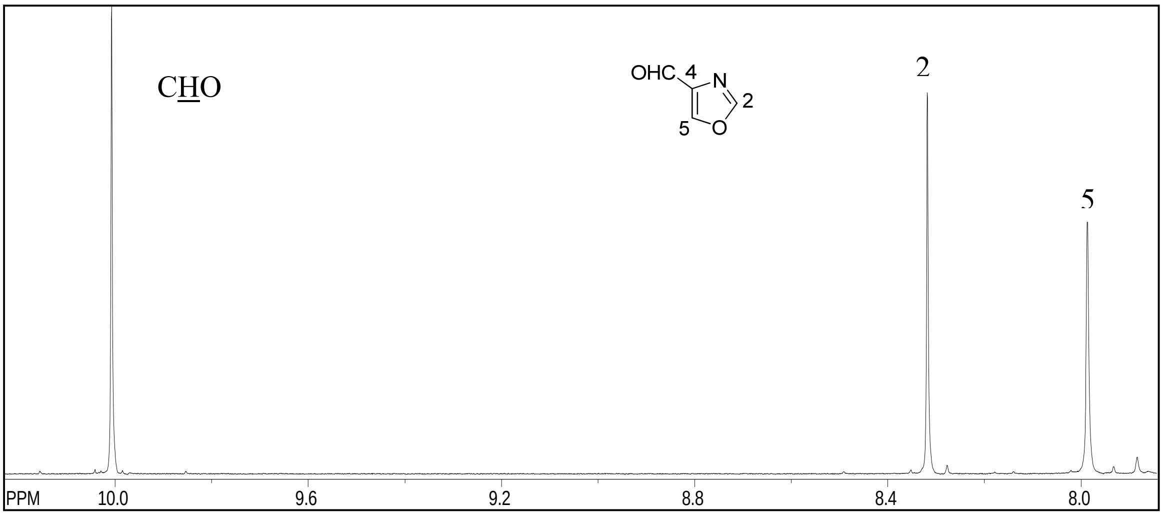

^{13}C NMR spectrum (75 MHz, 1s, 3d) ($CDCl_3$) (**3**)

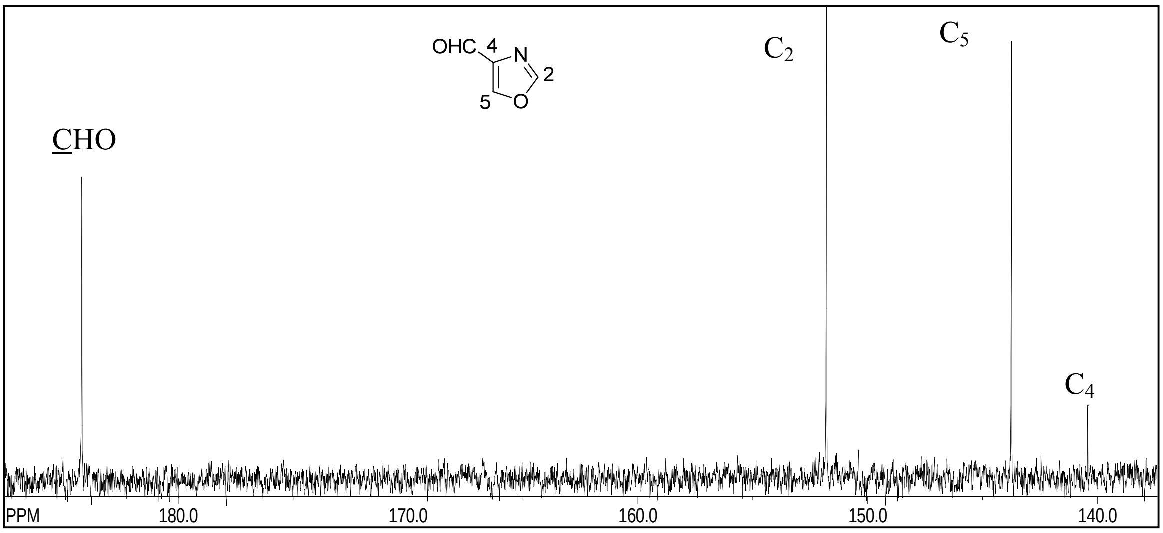

^{1}H NMR entire range spectrum (600 MHz, $CDCl_3$) (**3**)

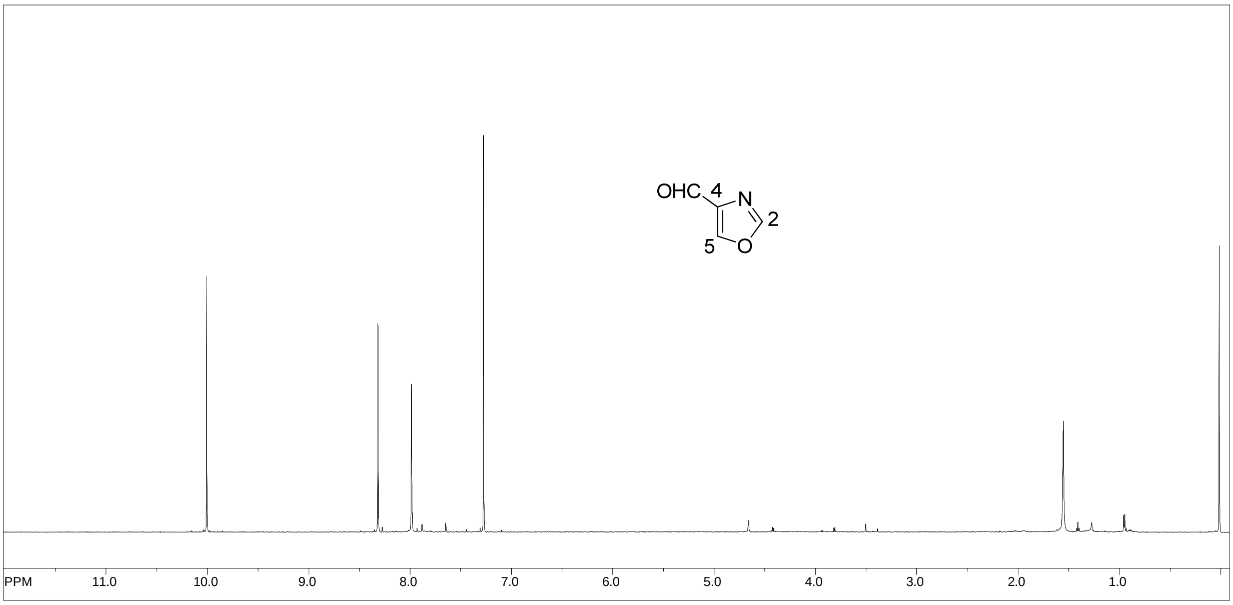

^{13}C NMR entire range spectrum (75 MHz, 1s, 3d) ($CDCl_3$) (**3**)

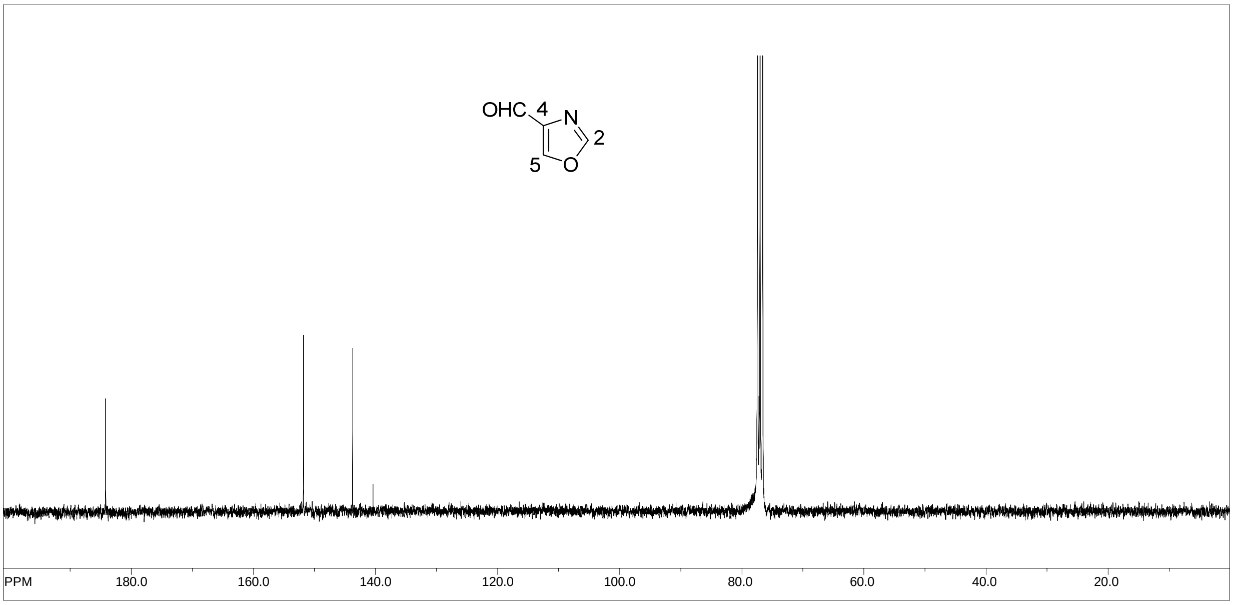

Oxazole-5-carbaldehyde[2] (compound 4)

^{1}H NMR spectrum (600 MHz, $CDCl_3$) (**4**)

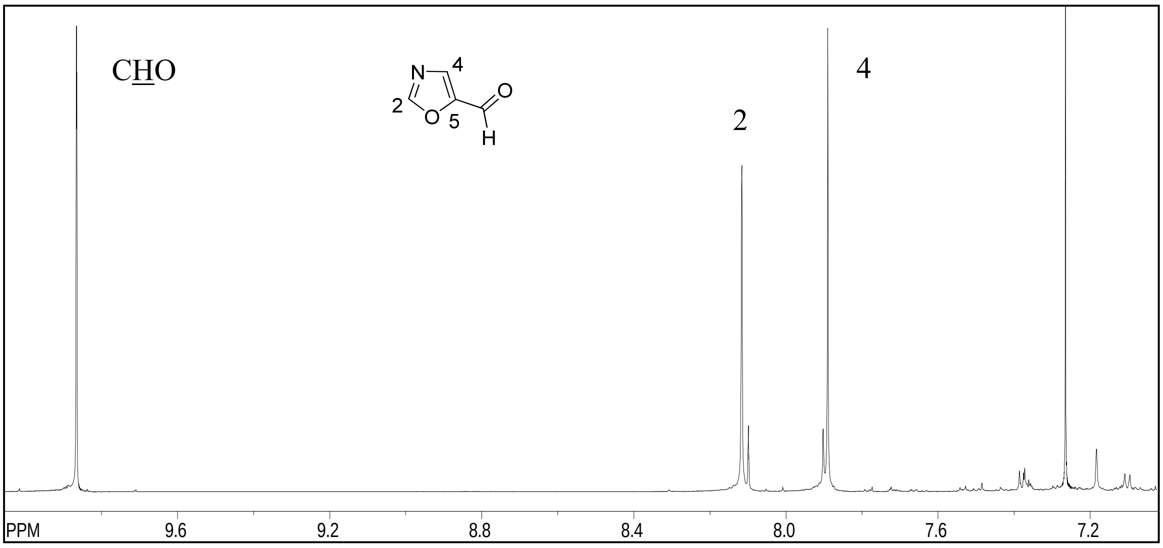

^{13}C NMR spectrum (1s, 3d) (150 MHz, $CDCl_3$) (**4**)

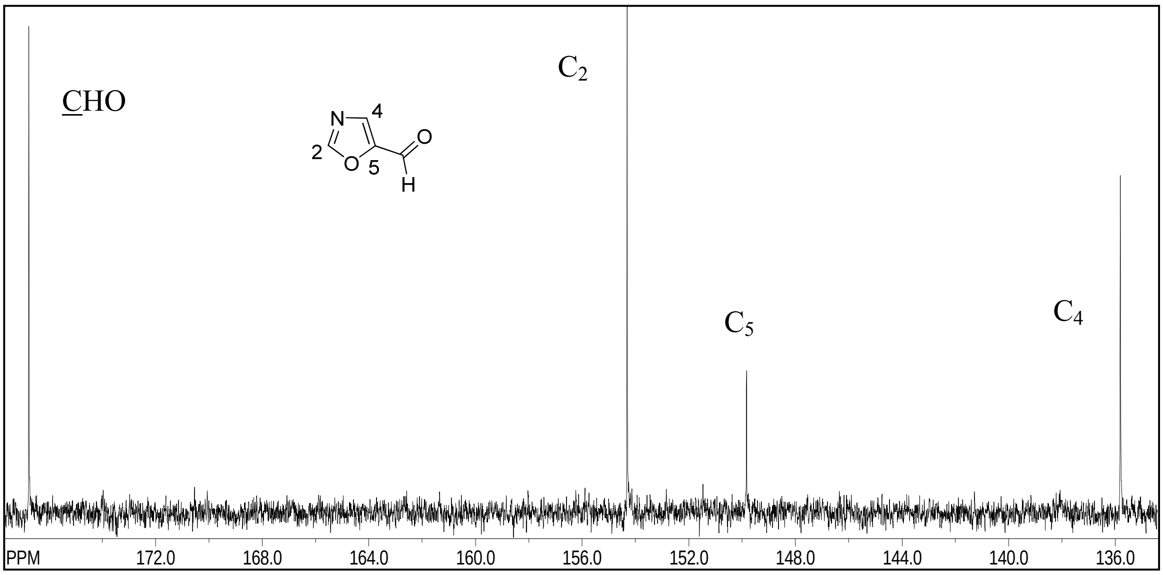

^1H NMR entire range spectrum of crude reaction mixture (600 MHz, $CDCl_3$) (**4**)

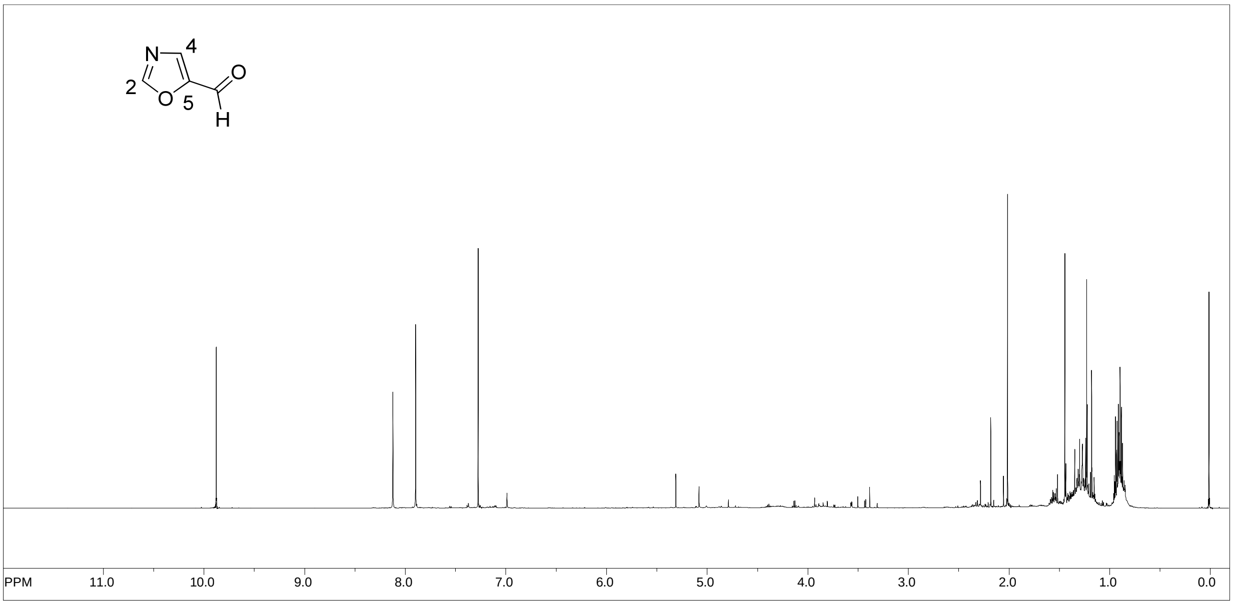

^{13}C NMR entire range spectrum of crude reaction mixture (1s, 3d) (150 MHz, $CDCl_3$) (**4**)

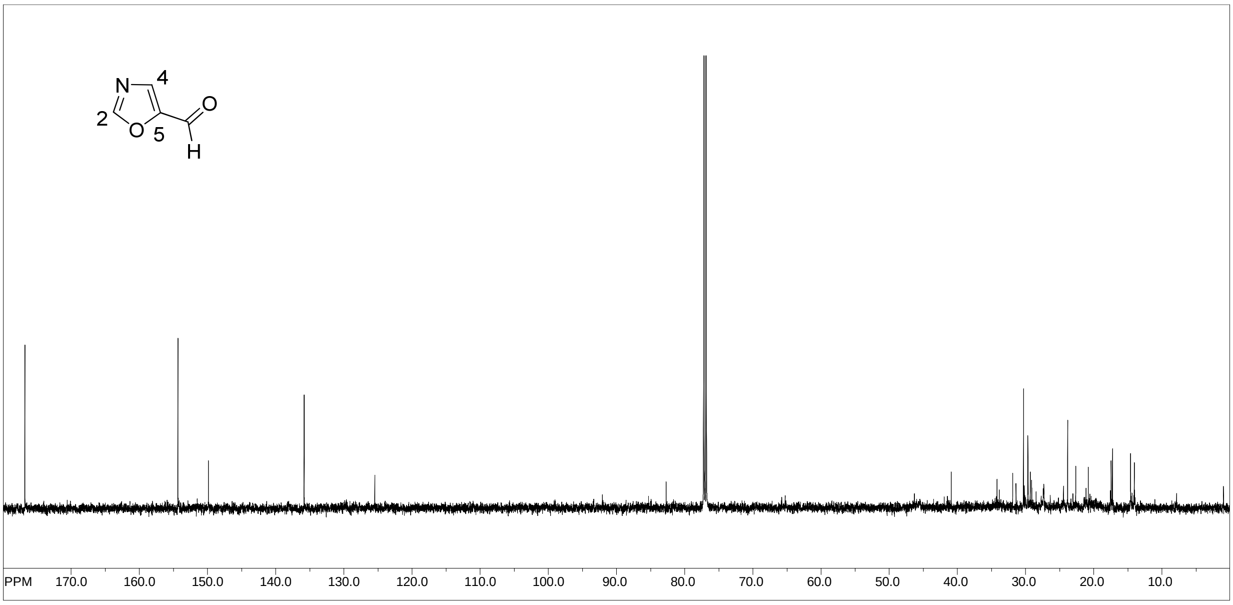

2-Vinylbenzaldehyde[3] (compound 5)

^{1}H NMR spectrum (600 MHz, $CDCl_3$)

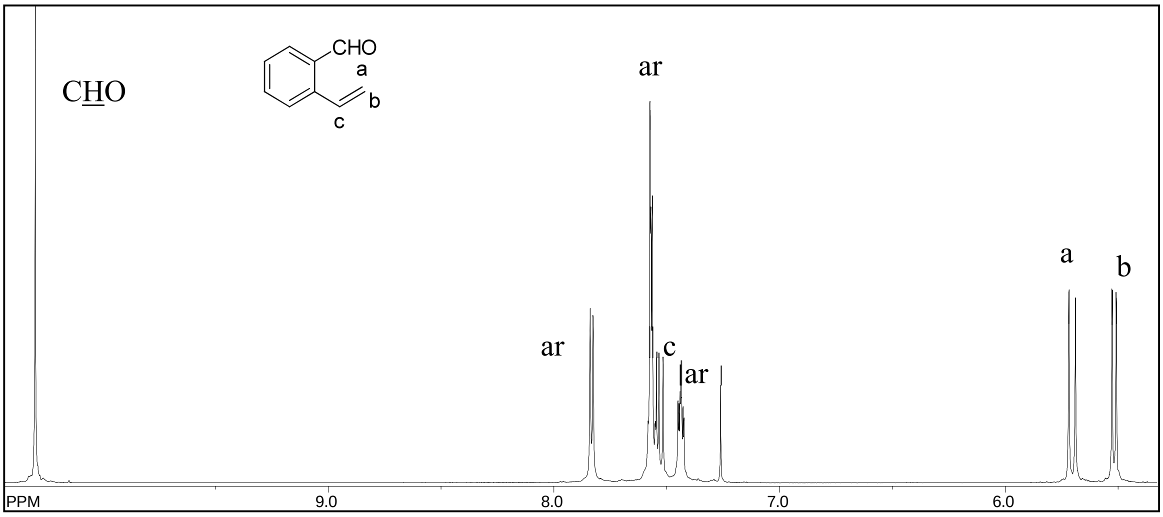

^{1}H NMR entire range spectrum (600 MHz, $CDCl_3$)

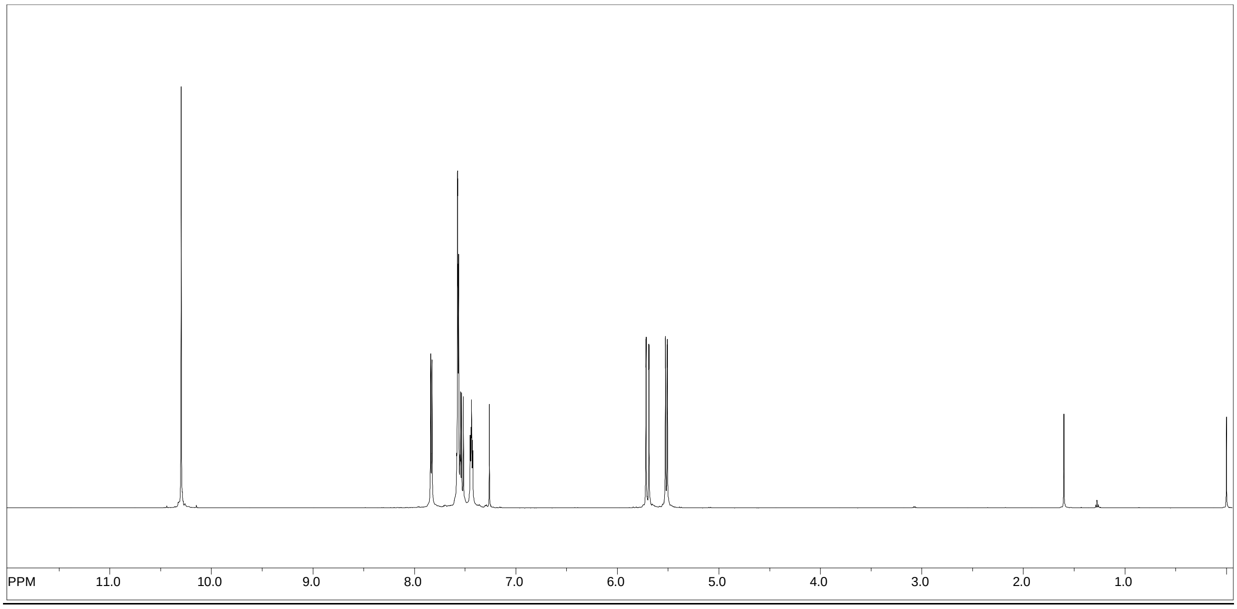

***trans*-3-(2-Ethenylphenyl)prop-2-enal (compound 6)**

^{1}H NMR spectrum (300 MHz, $CDCl_3$) (**6**)

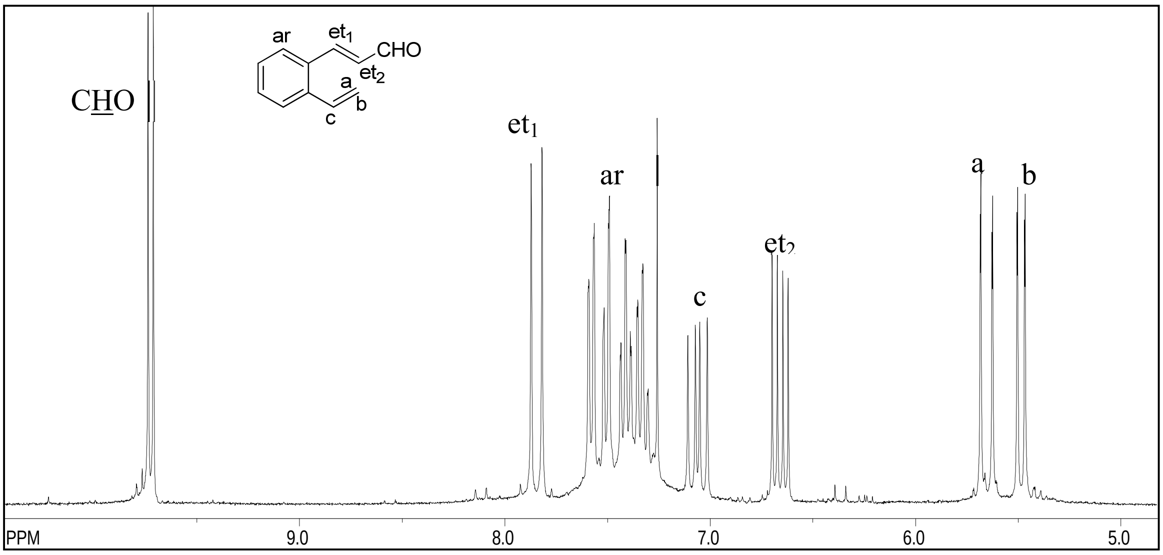

^{13}C NMR spectrum (2s, 8d, 1t) (75 MHz, $CDCl_3$) (**6**)

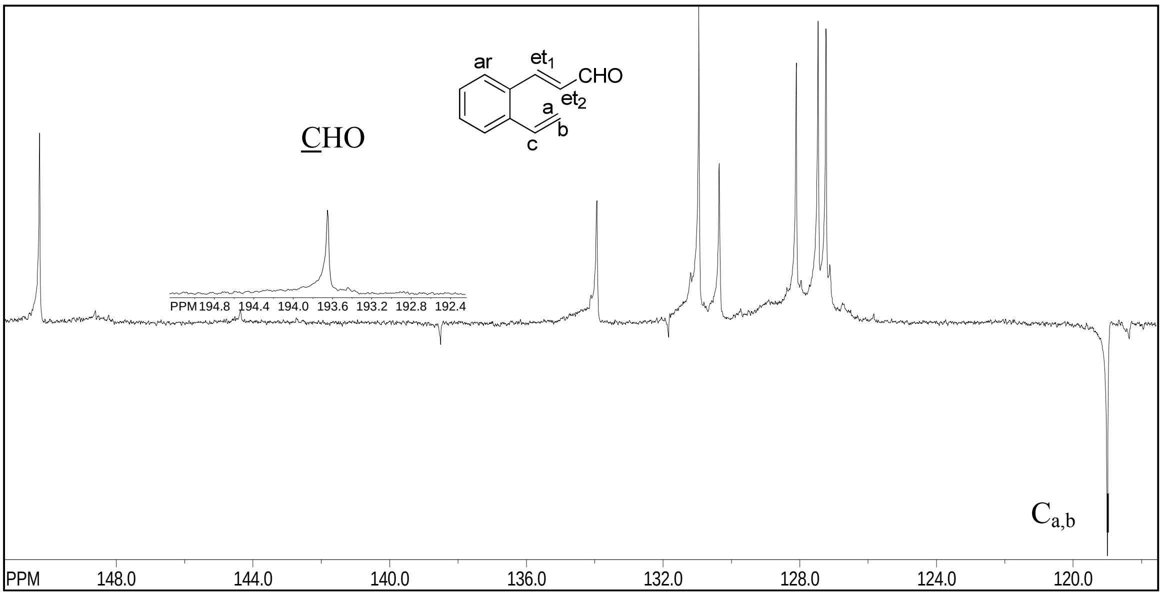

^{1}H NMR entire range spectrum (300 MHz, $CDCl_3$) (**6**)

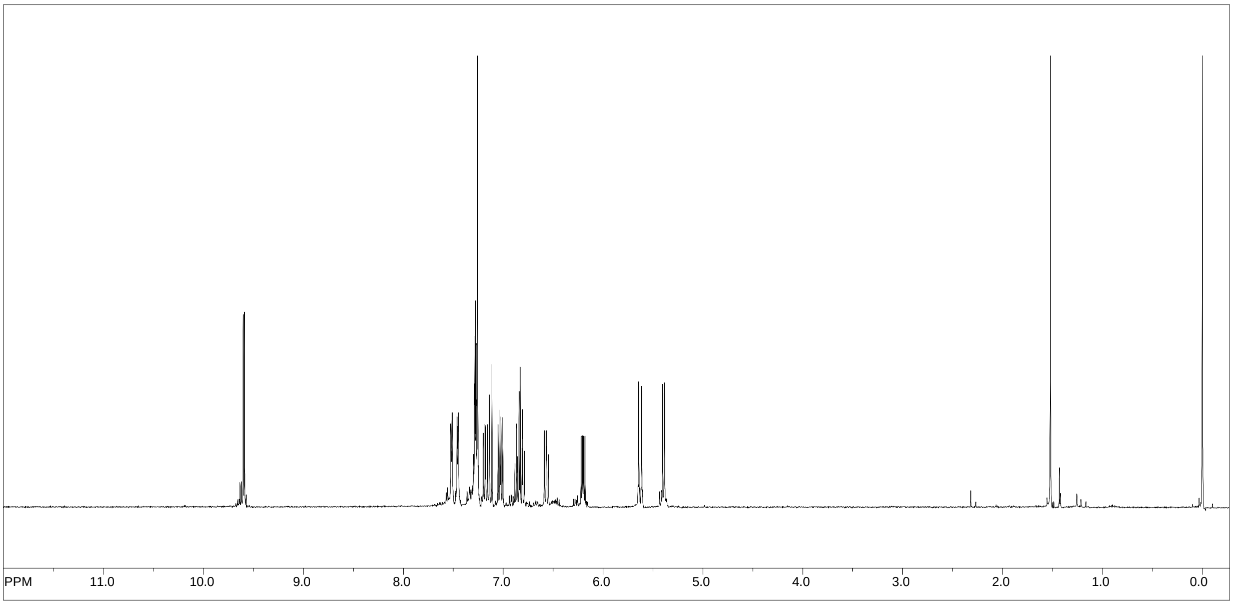

^{13}C NMR entire range spectrum (2s, 8d, 1t) (75 MHz, $CDCl_3$) (**6**)

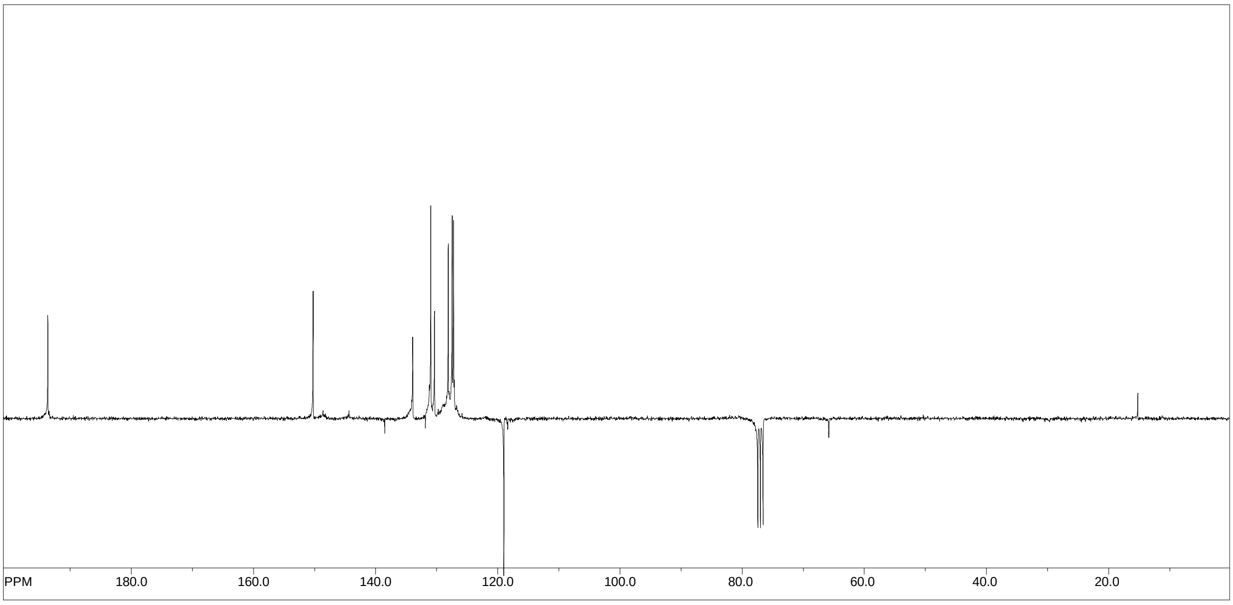

***trans,trans*-5-(2-Vinylphenyl)penta-2,4-dienal (compound 7)**

^{1}H NMR spectrum (600 MHz, $CDCl_3$) (**7**)

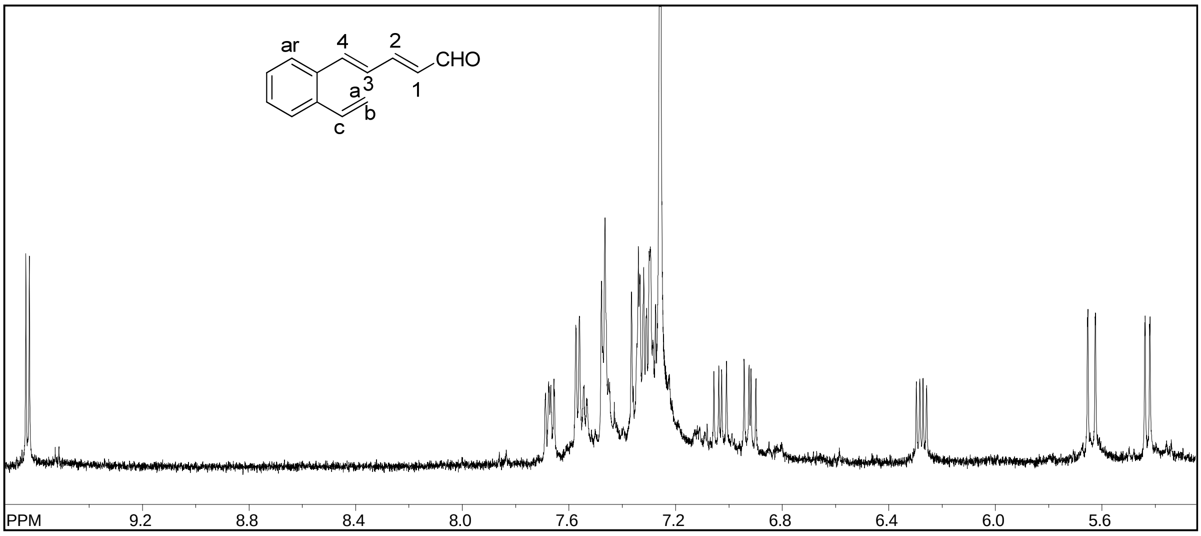

^{1}H NMR entire range spectrum (600 MHz, $CDCl_3$) (**7**) (peaks beetwen 0.8-1.2 ppm are residue from petroleumether)

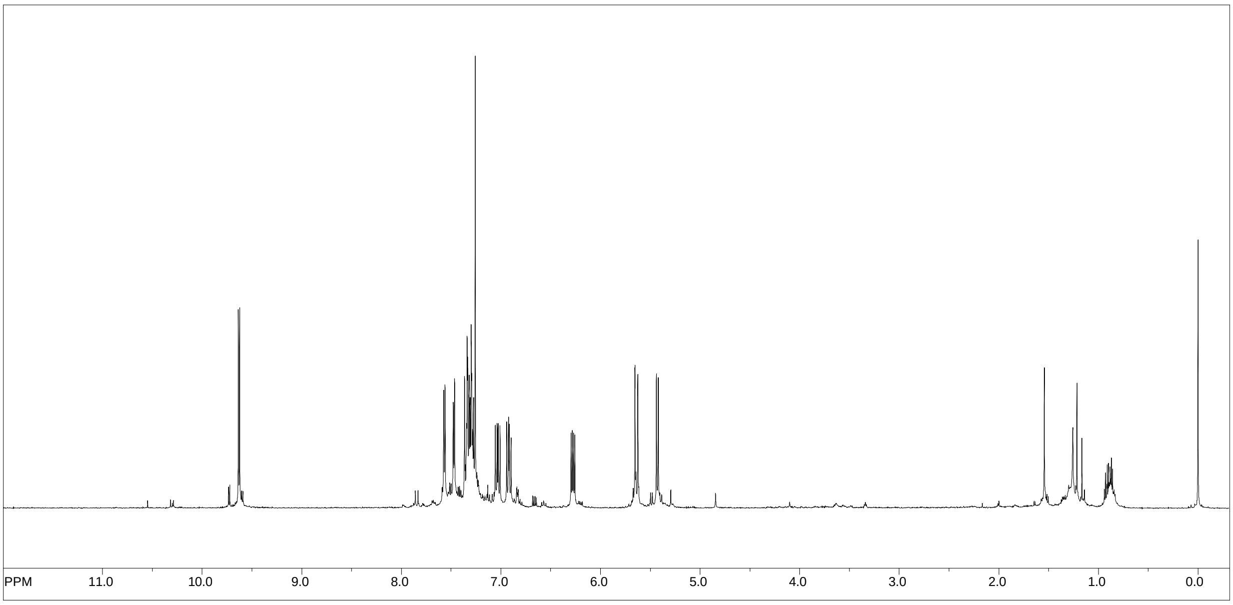

***cis*-4-2-(2-Vinylphenyl)ethenyl]oxazole** (**compound** *cis*-**1)**

^{1}H NMR spectrum (600 MHz, $CDCl_3$) (*cis*-**1**) (contains traces of *trans*-isomer)

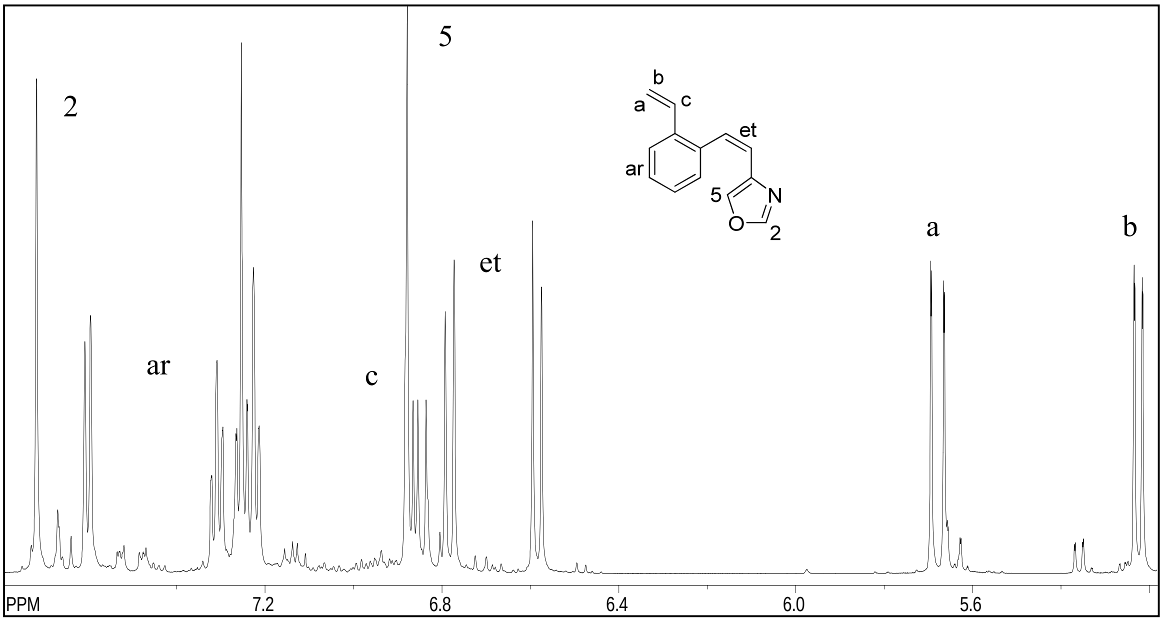

^{13}C NMR spectrum (150 MHz, 3s, 9d, 1t) ($CDCl_3$) (*cis*-**1**)

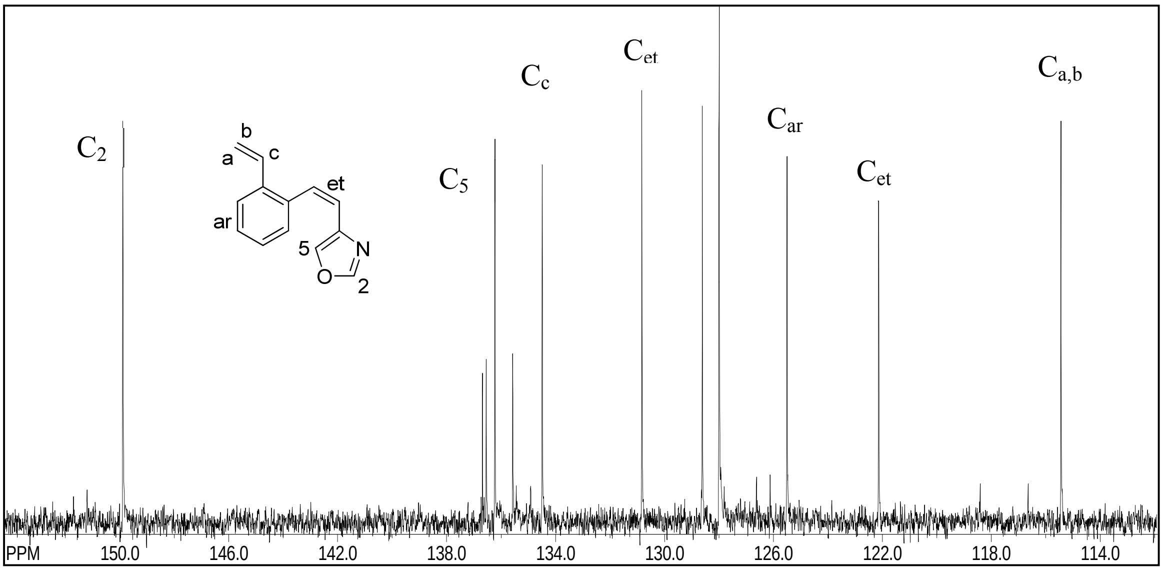

^{1}H NMR entire range spectrum (600 MHz, $CDCl_3$) (*cis*-**1**)

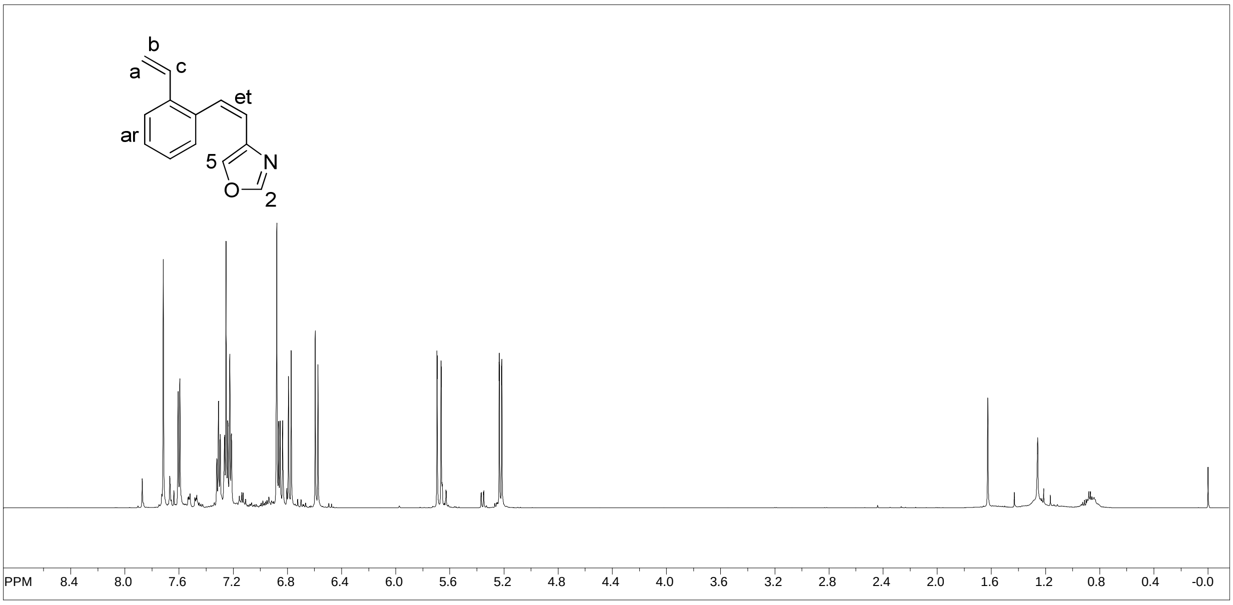

^{13}C NMR entire range spectrum (150 MHz, 3s, 9d, 1t) ($CDCl_3$) (*cis*-**1**)

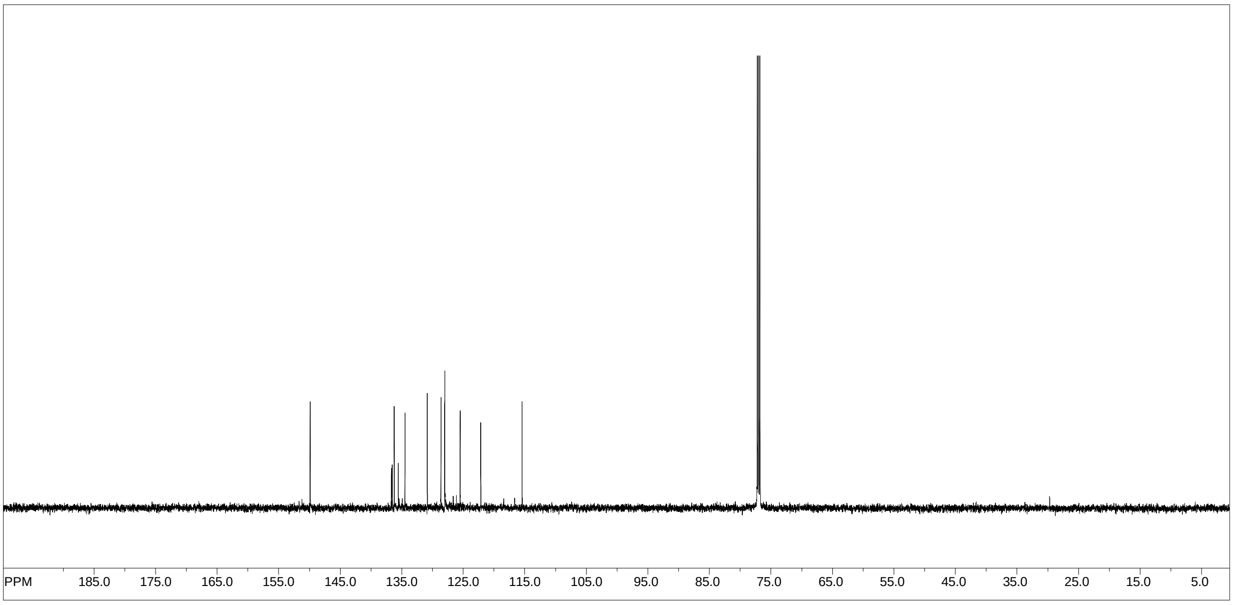

***trans*-4-2-(2-Vinylphenyl)ethenyl]oxazole** (**compound** *trans*-**1**)

^{1}H NMR spectrum (600 MHz, $CDCl_3$) (*trans*-**1**)

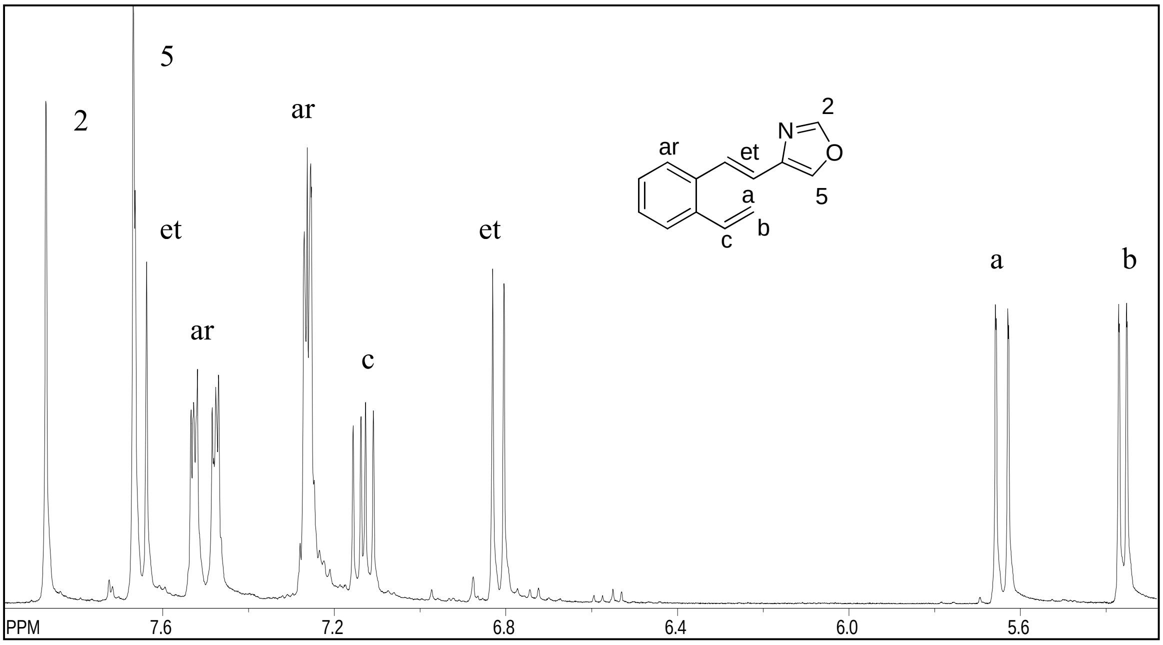

^{1}H NMR spectrum (600 MHz, C_6D_6) (*trans*-**1**)

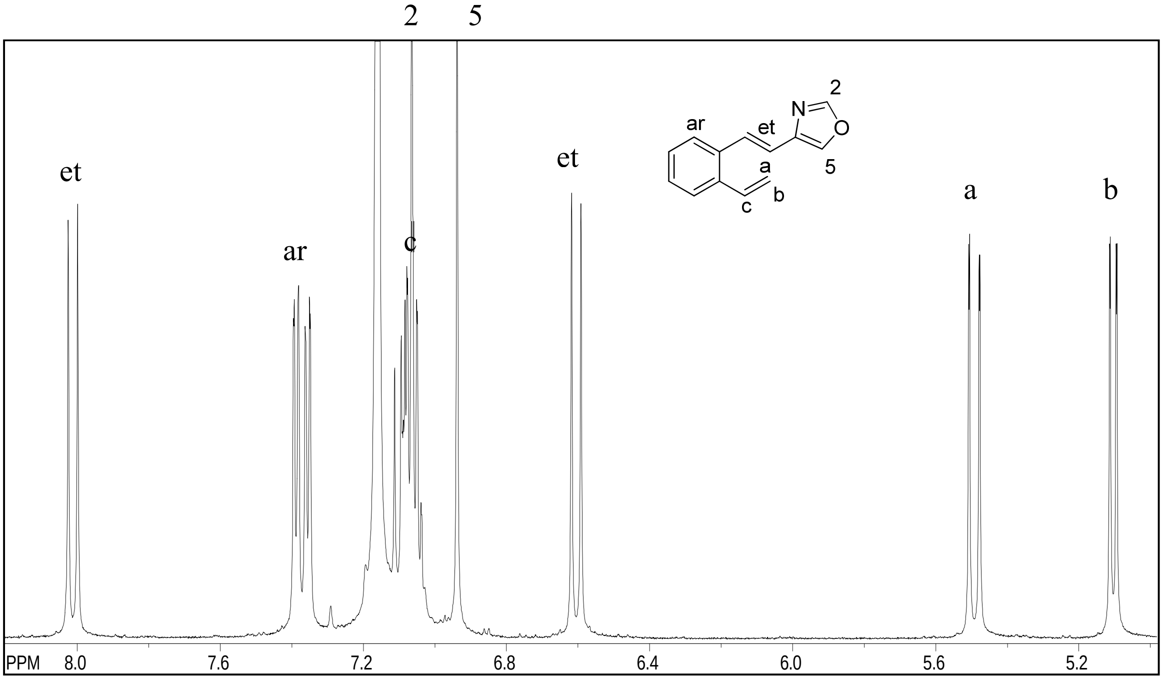

^{13}C NMR spectrum (150 MHz, 3s, 9d, 1t) ($CDCl_3$) (*trans*-**1**)

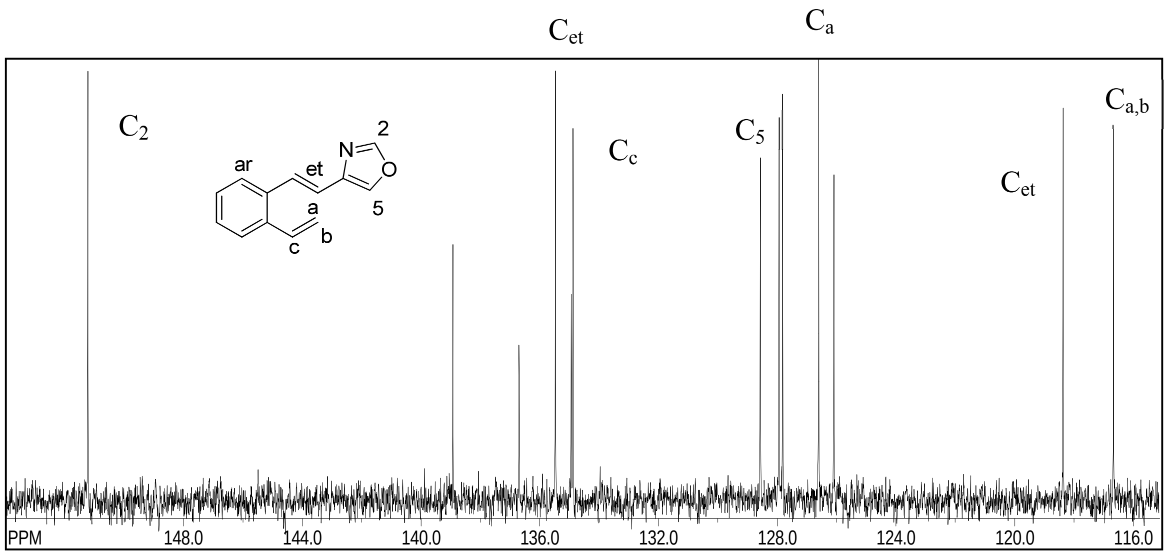

^{1}H NMR entire range spectrum (600 MHz, $CDCl_3$) (*trans*-**1**)

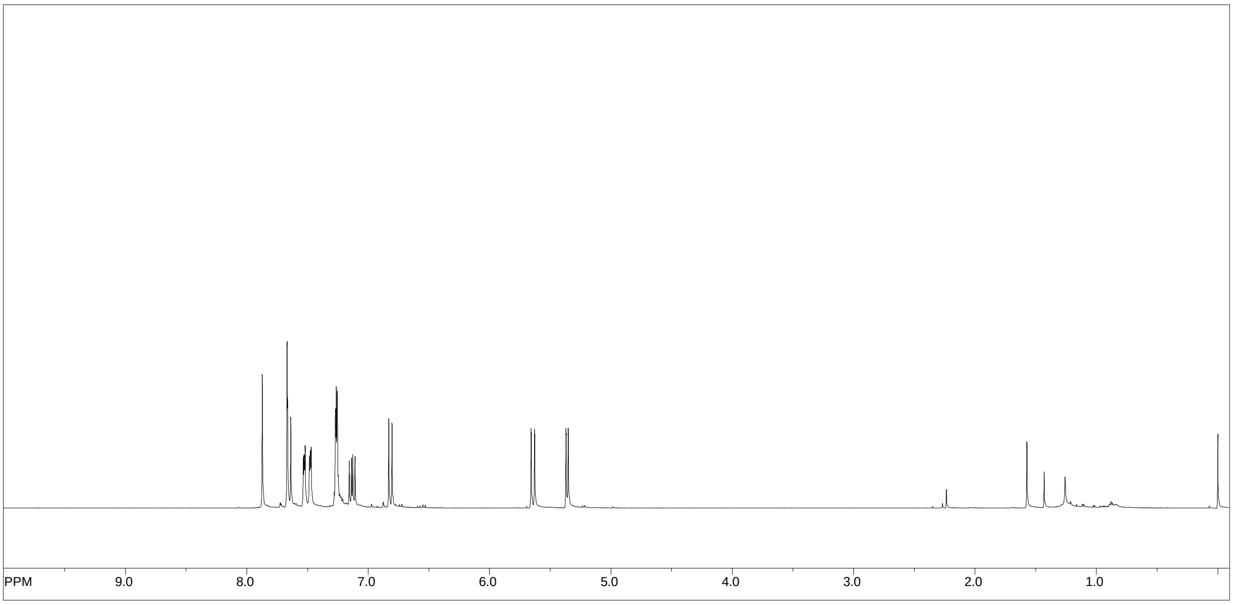

^{13}C NMR entire range spectrum (150 MHz, 3s, 9d, 1t) ($CDCl_3$) (*trans*-**1**)

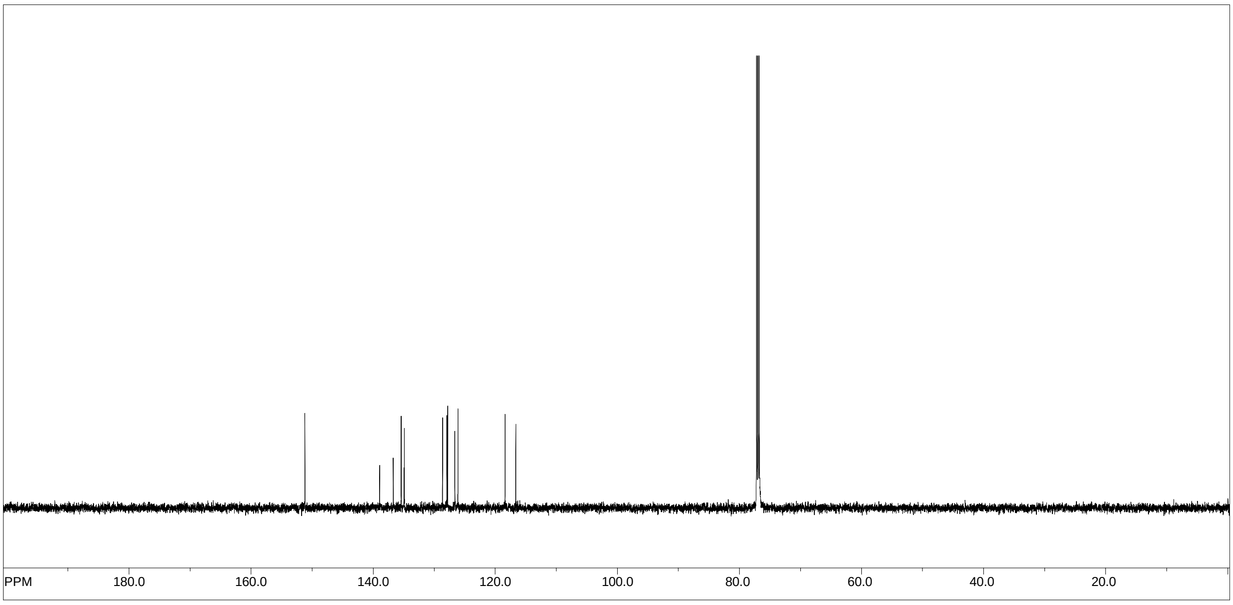

***cis*-5-[2-(2-Vinylphenyl)ethenyl]oxazole** (**compound** *cis*-**2)** (contains traces of *trans*-isomer)
^{1}H NMR spectrum (600 MHz, $CDCl_3$) (*cis*-**2**)

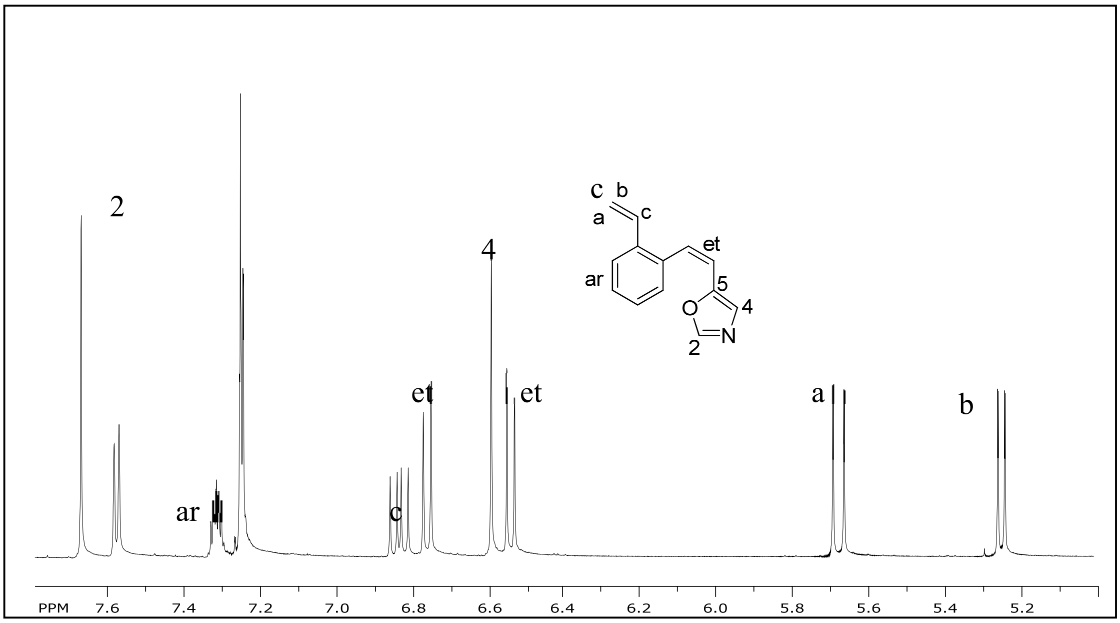

^{13}C NMR spectrum (3s, 8d, 1t) (150 MHz, $CDCl_3$) (*cis*-**2**)

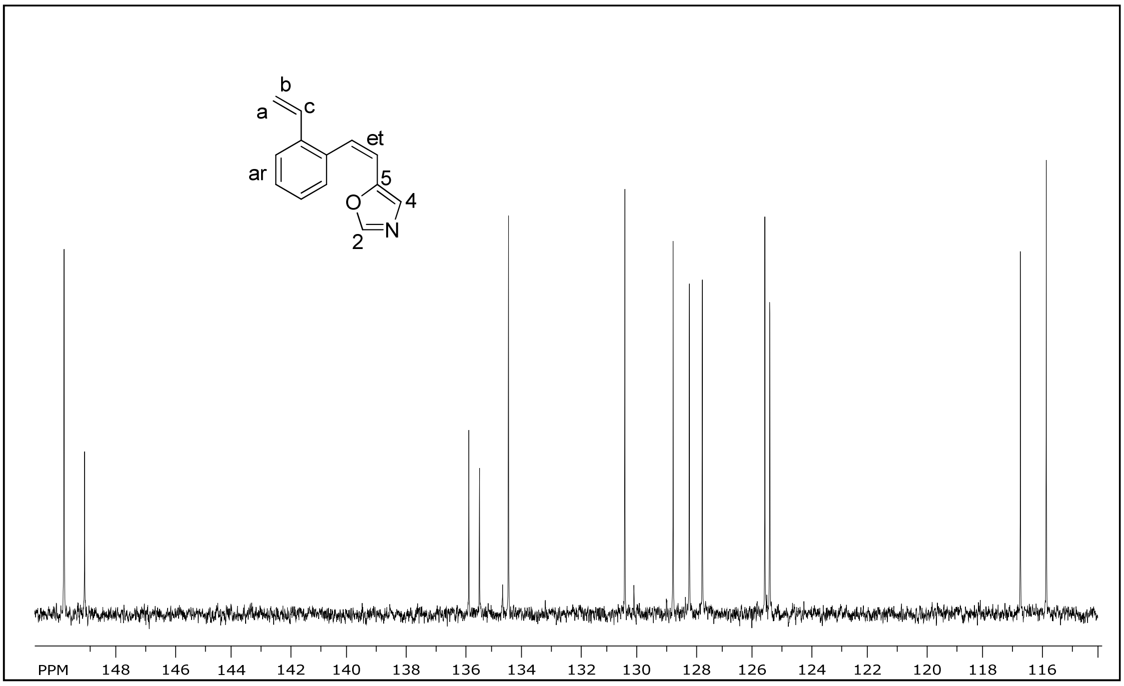

^{1}H NMR entire range spectrum (600 MHz, $CDCl_3$) (*cis*-**2**)

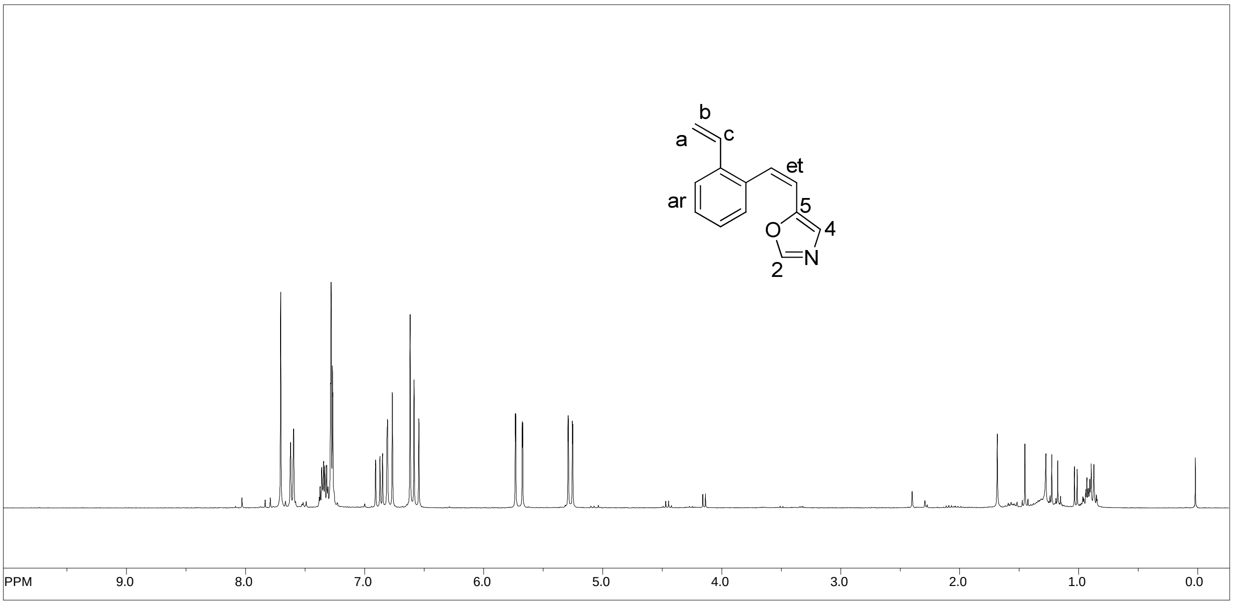

^{13}C NMR entire range spectrum (3s, 8d, 1t) (150 MHz, $CDCl_3$) (*cis*-**2**)

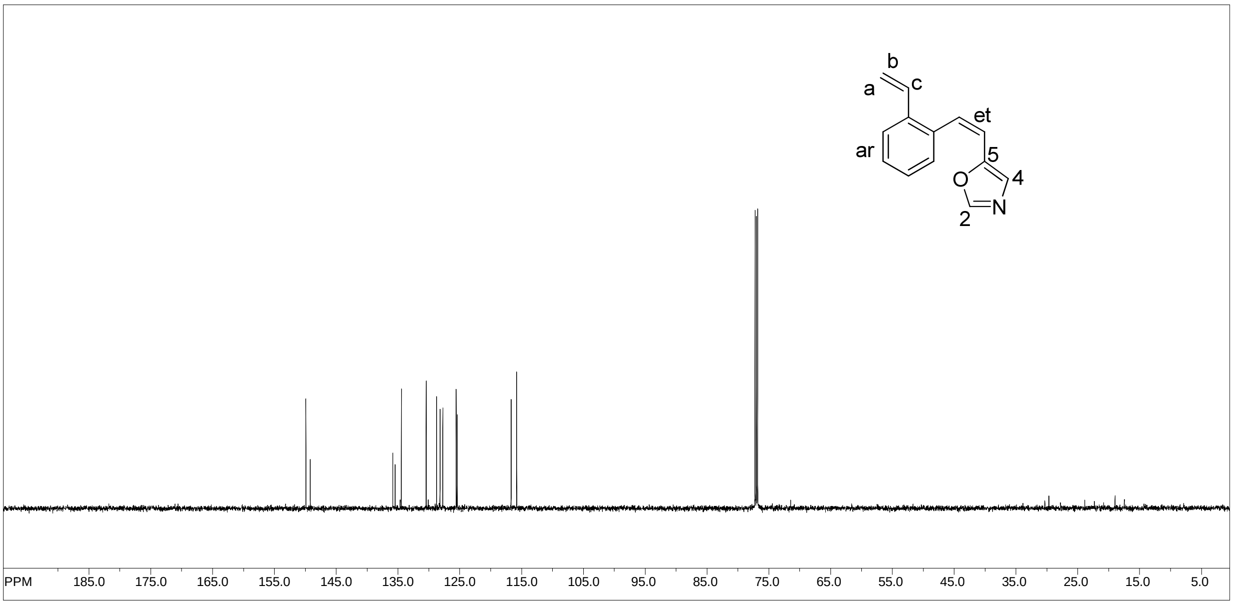

***trans*-5-[2-(2-Vinylphenyl)ethenyl]oxazole** (**compound** *trans*-**2)**

^{1}H NMR spectrum (600 MHz, $CDCl_3$) (*trans*-**2**)

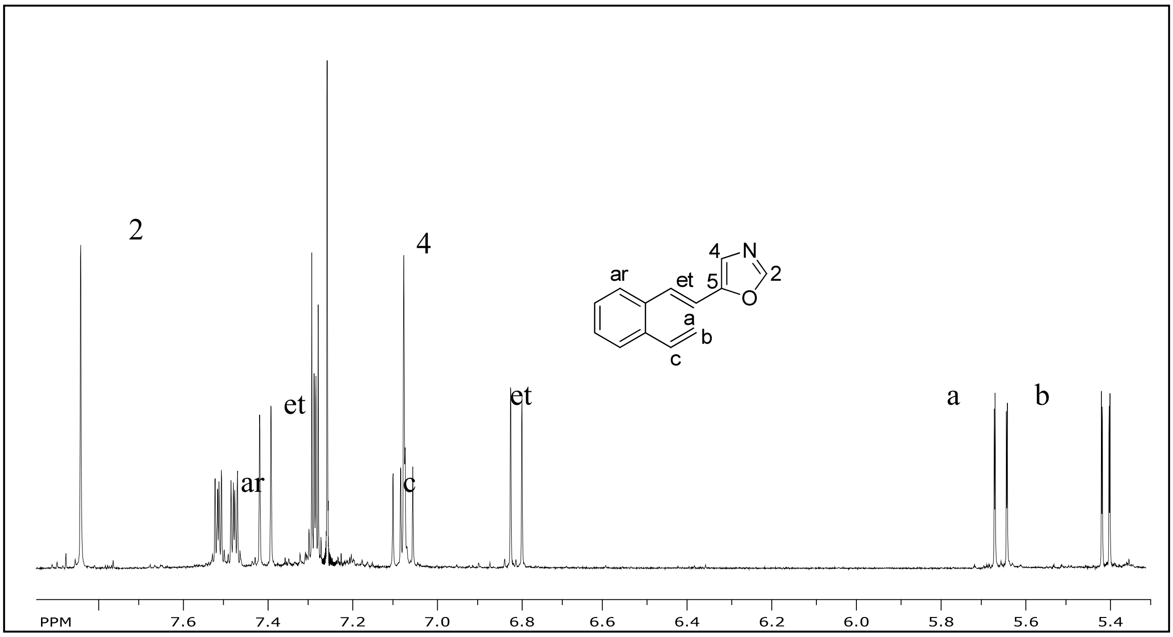

^{13}C NMR spectrum (3s, 8d, 1t) (150 MHz, $CDCl_3$) (*trans*-**2**)

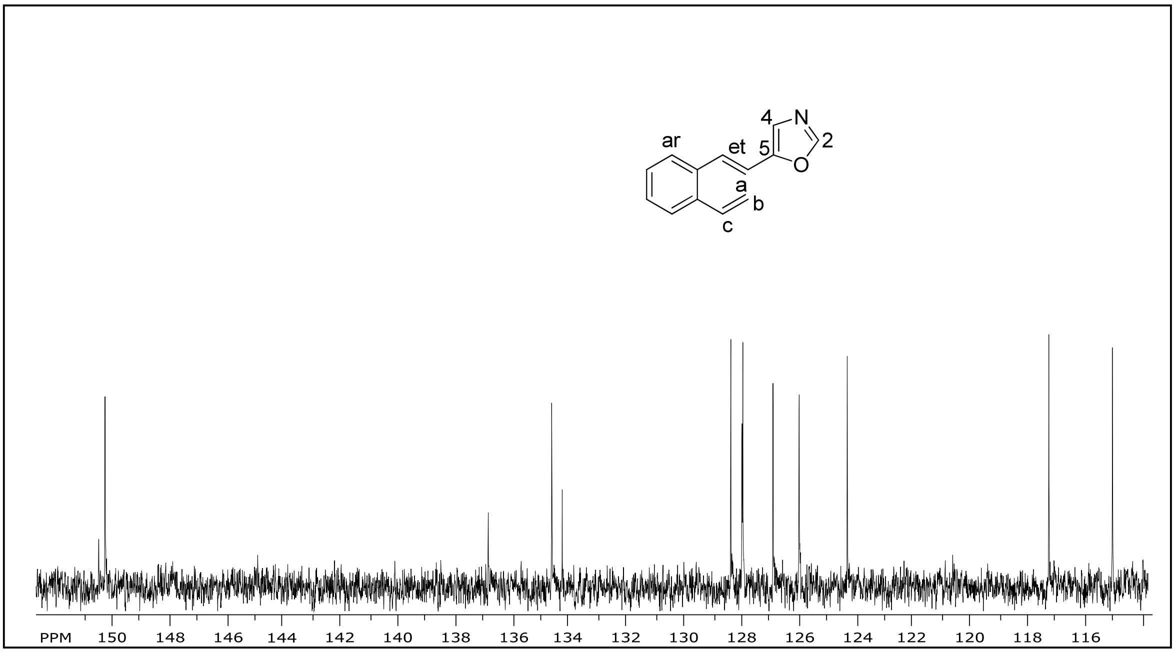

^{1}H NMR entire range spectrum (600 MHz, $CDCl_3$) (*trans*-**2**)

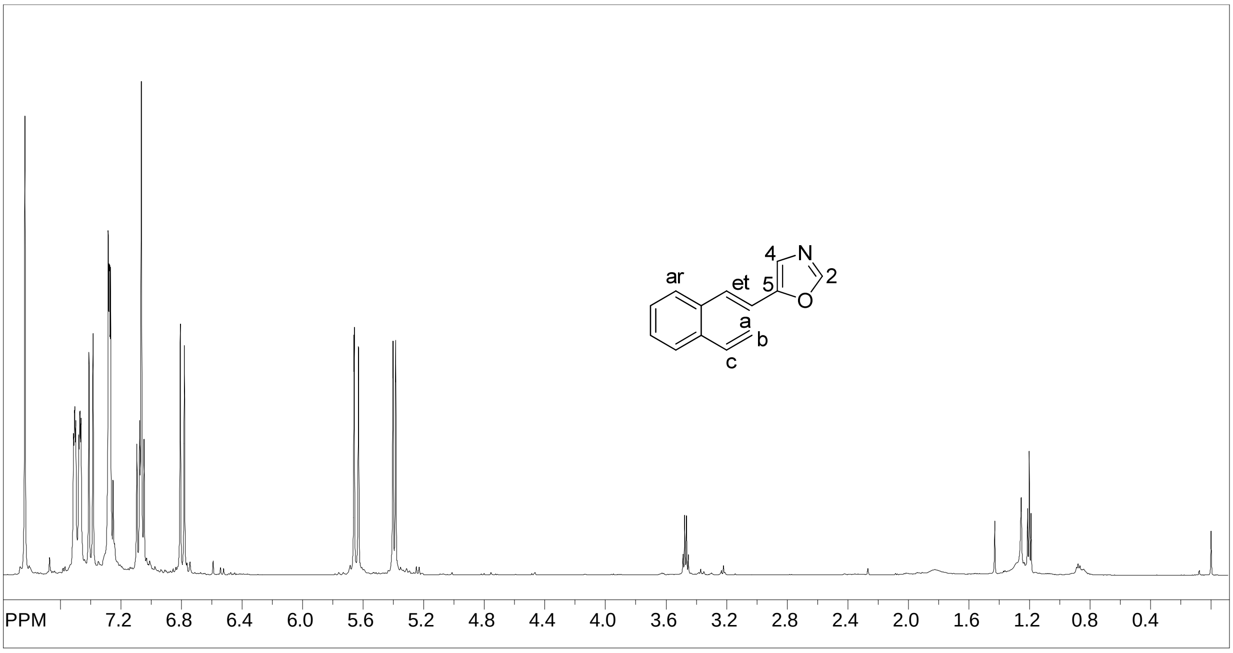

^{13}C NMR entire range spectrum (3s, 8d, 1t) (150 MHz, $CDCl_3$) (*trans*-**2**)

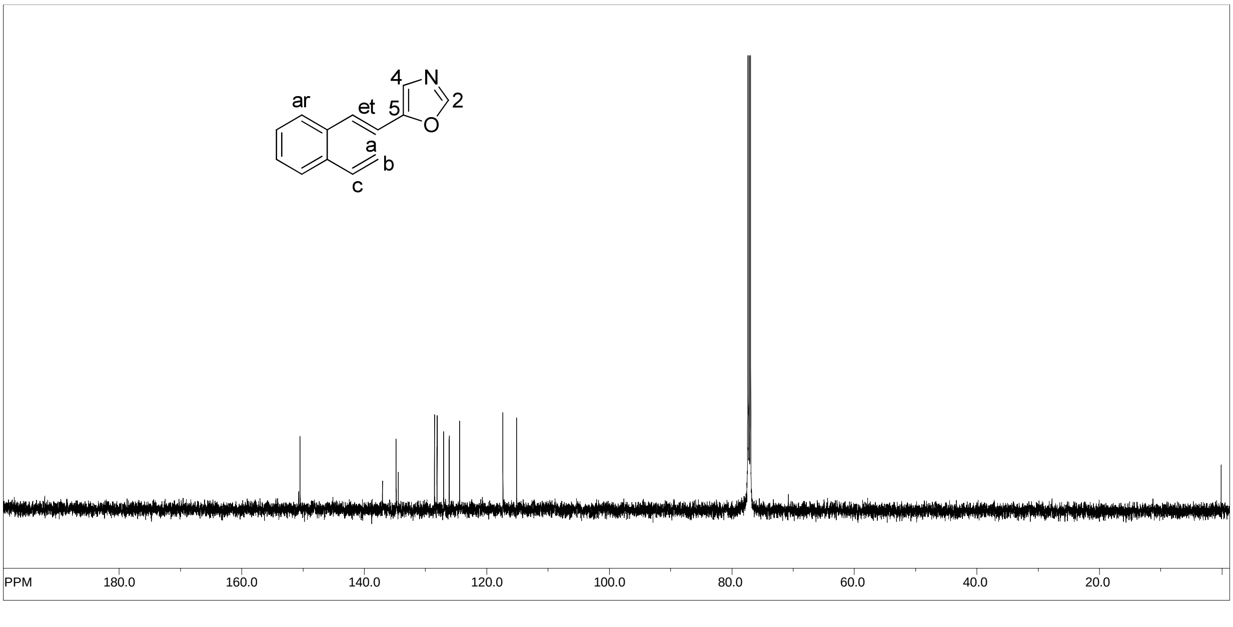

***rel*-(2*S*)-3-Oxa-5-azatetracyclo[6.6.1.0^{2,6}.0^{9,14}]pentadeca-4,6,9,11,13-pentaene (compound 8a)**

^{1} H NMR spectrum (600 MHz, $CDCl_3$) (**8a**)

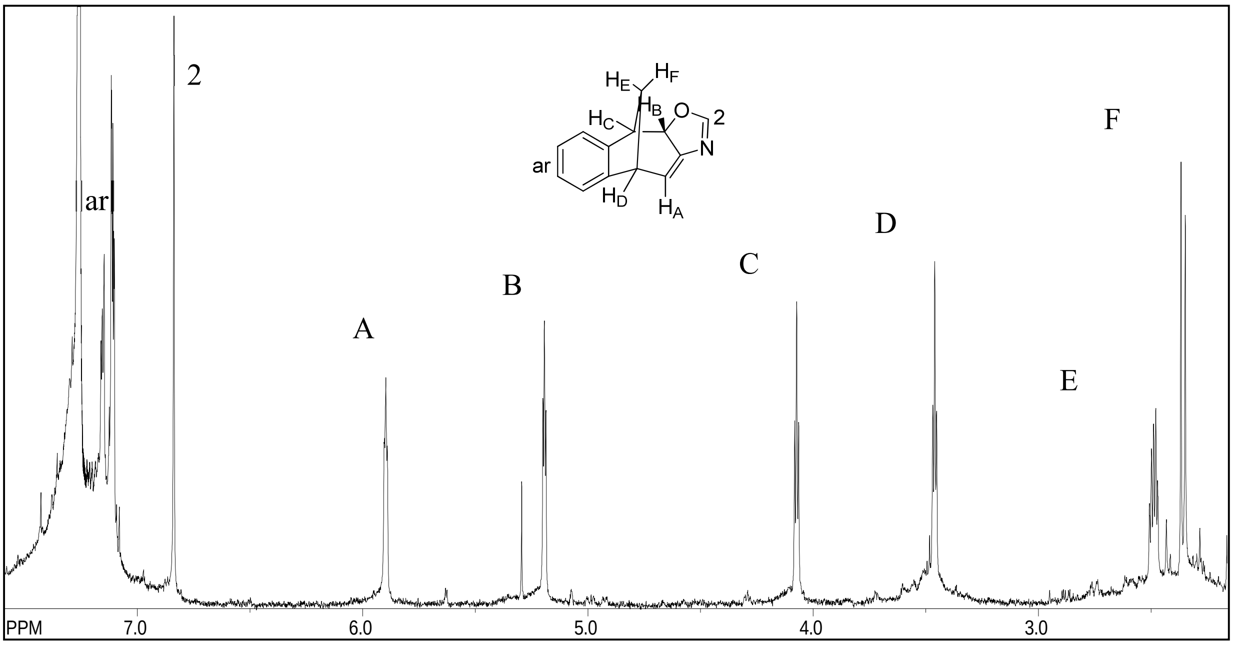

^{1} H NMR entire range spectrum (600 MHz, $CDCl_3$) (**8a**)

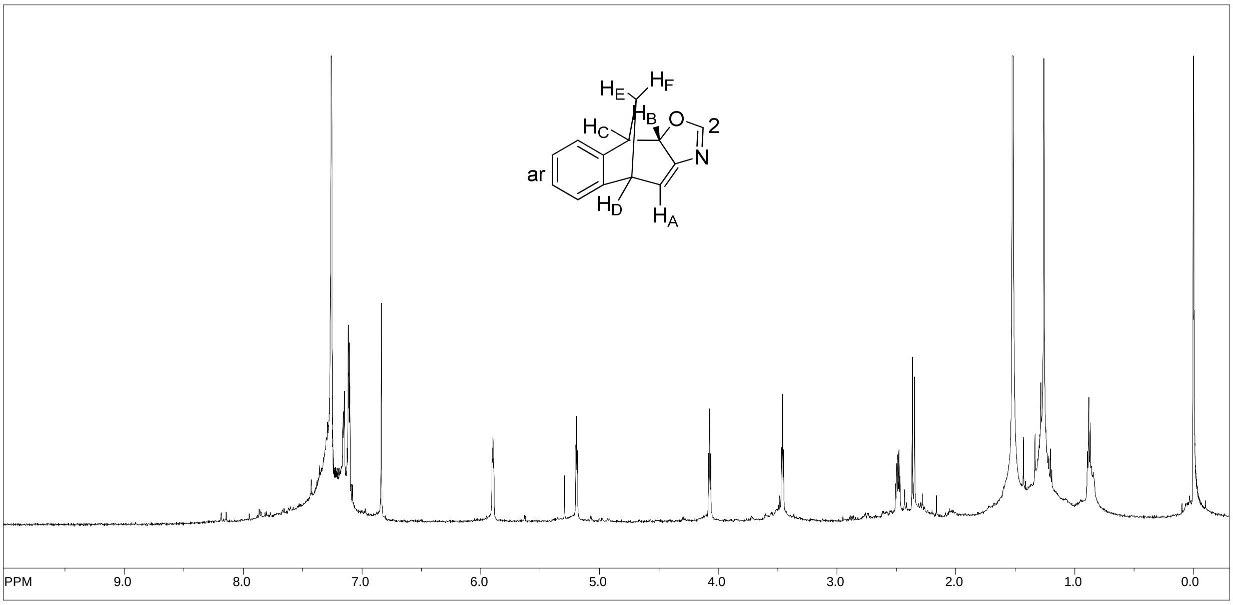

^{1}H NMR spectrum (600 MHz, $CDCl_3$) of crude mixture after irradiation (compound **8a**, **8b**, **9**)

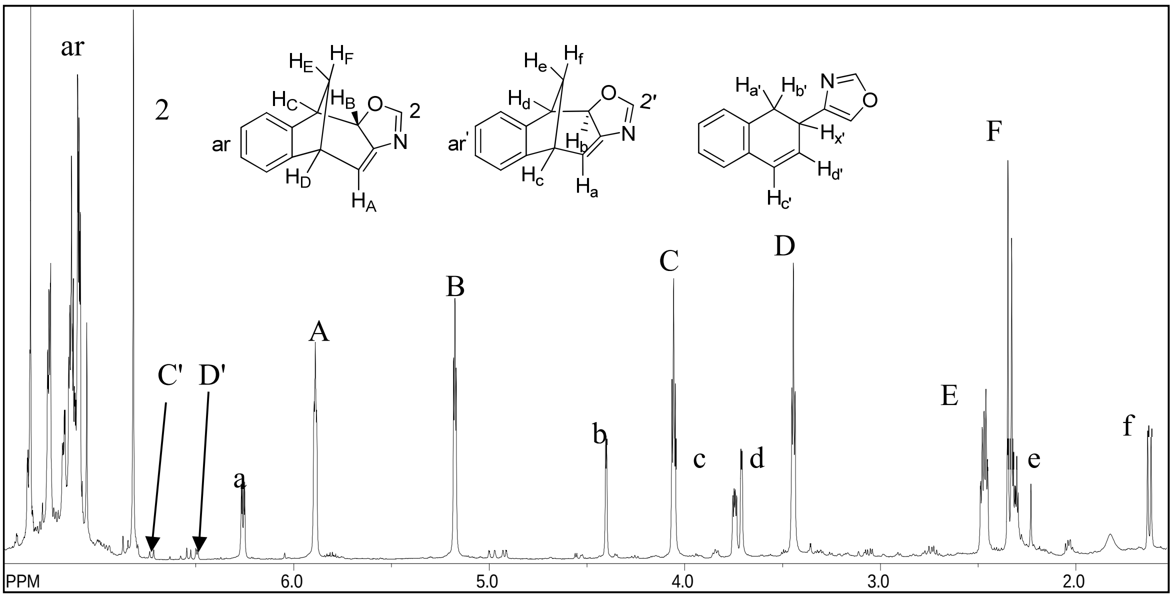

^{1}H NMR entire range spectrum (600 MHz, $CDCl_3$) of crude mixture after irradiation (compound **8a**, **8b**, **9**)

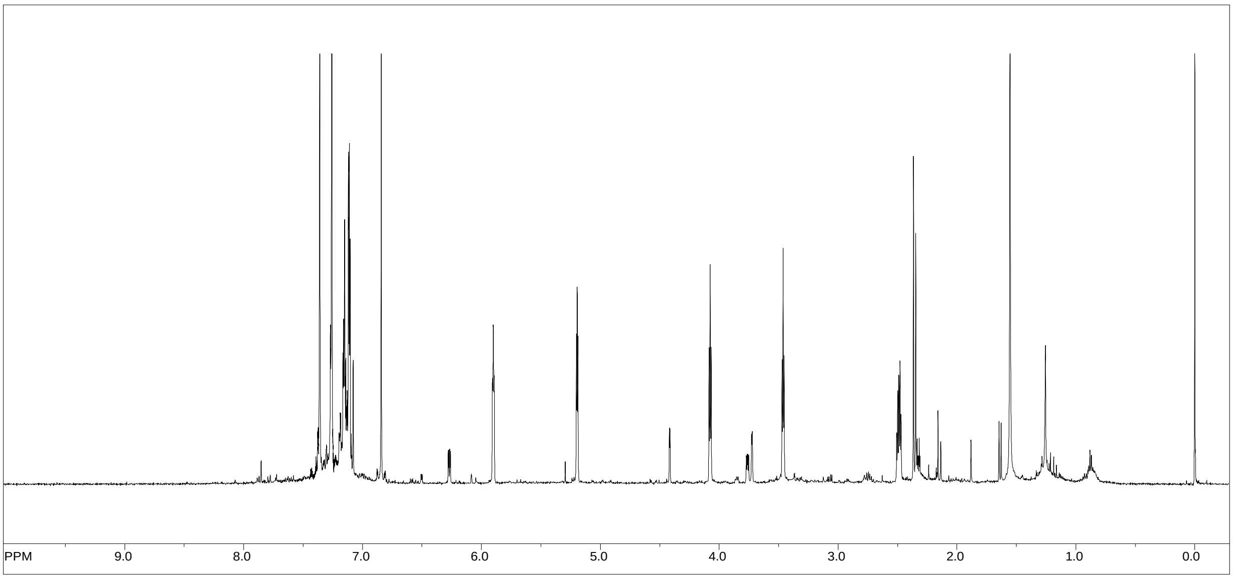

^{13}C NMR spectrum of crude mixture after irradiation (150 MHz, 3s, 9d, 1t) ($CDCl_3$) (**8a**, **8b**)

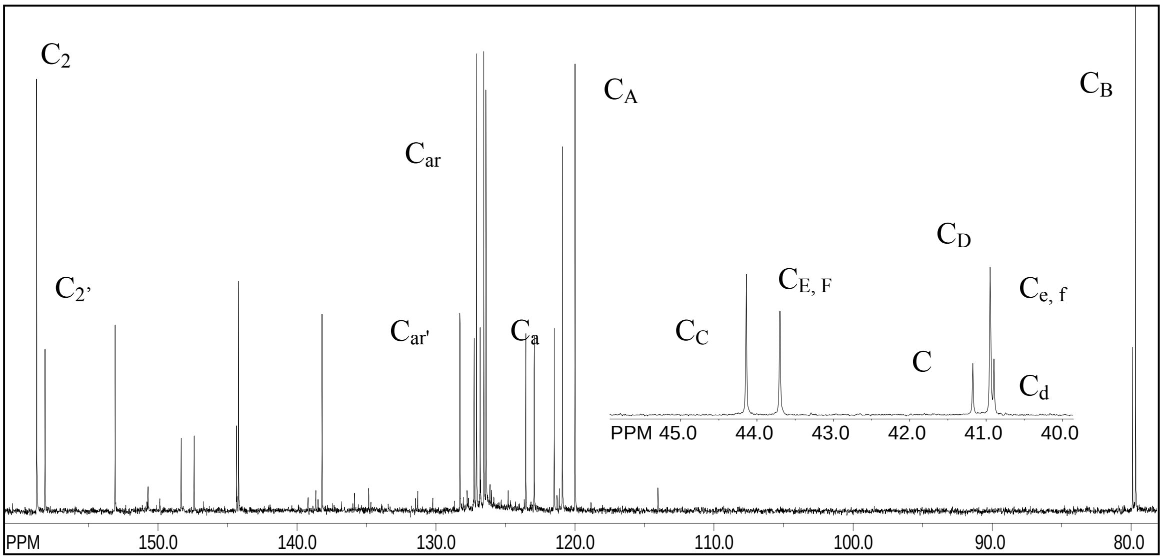

^{13}C NMR entire range spectrum of crude mixture after irradiation (150 MHz, 3s, 9d, 1t) ($CDCl_3$) (**8a**, **8b**)

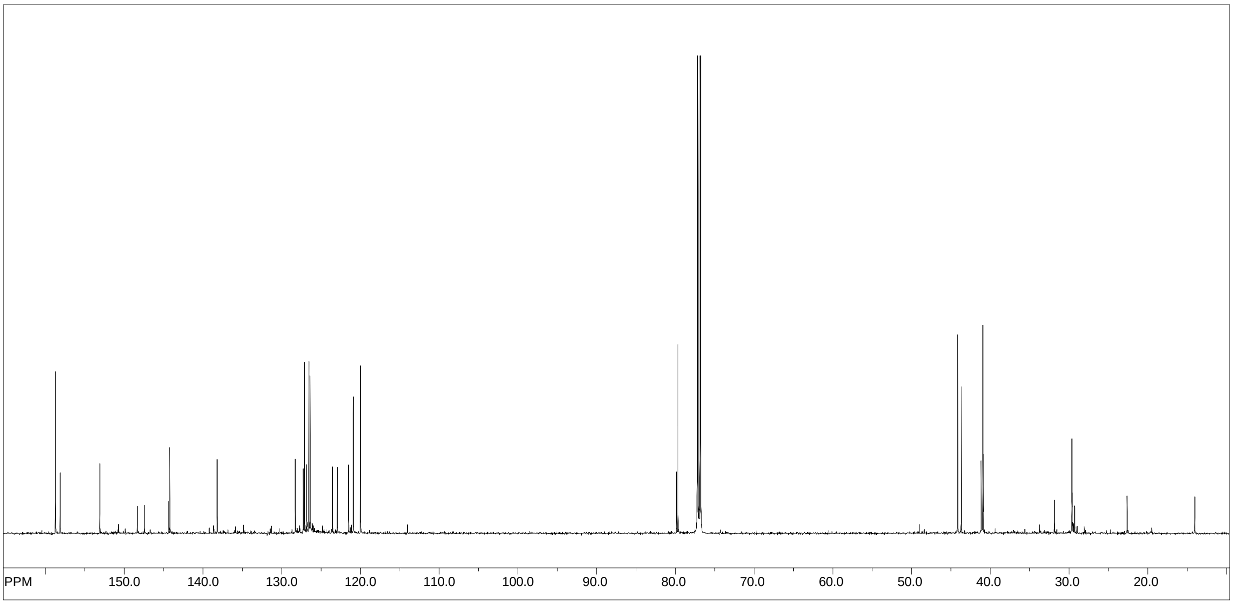

COSY spectrum of crude mixture after irradiation ($CDCl_3$) (**8a**, **8b**)

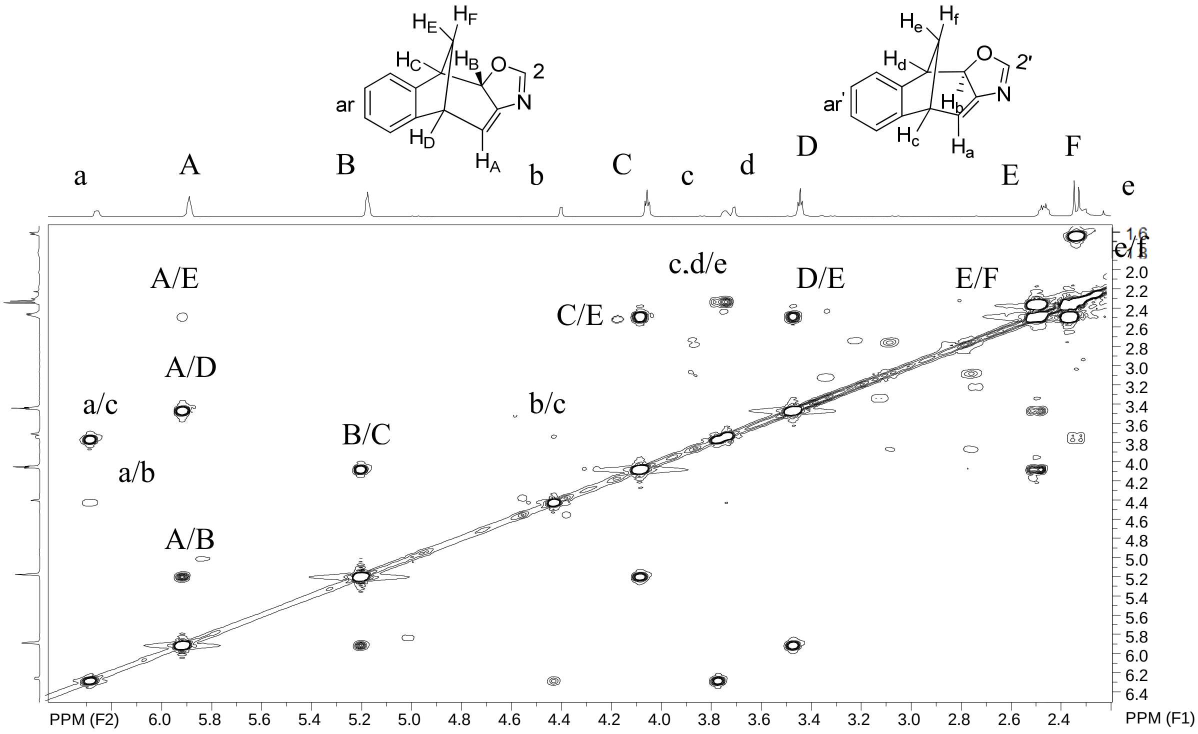

HSQC spectrum of crude mixture after irradiation ($CDCl_3$) (**8a**, **8b**)

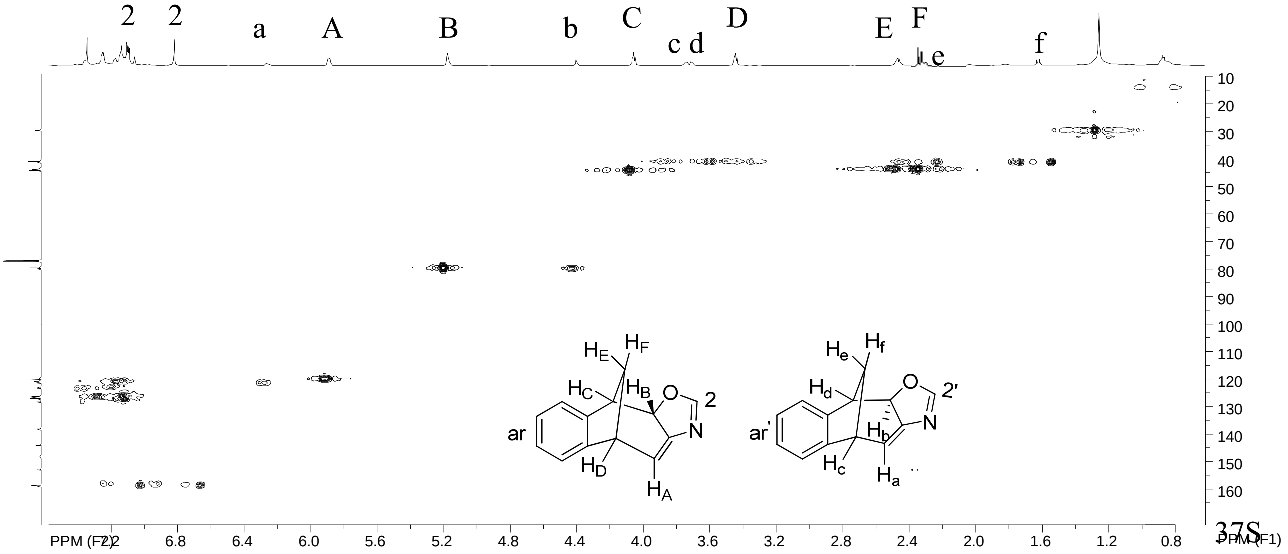

NOESY spectrum of crude mixture after irradiation ($CDCl_3$) (**8a**, **8b**)

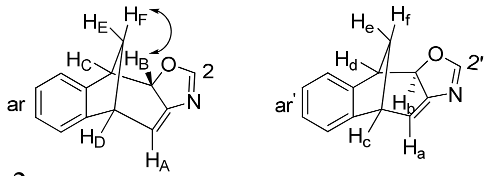

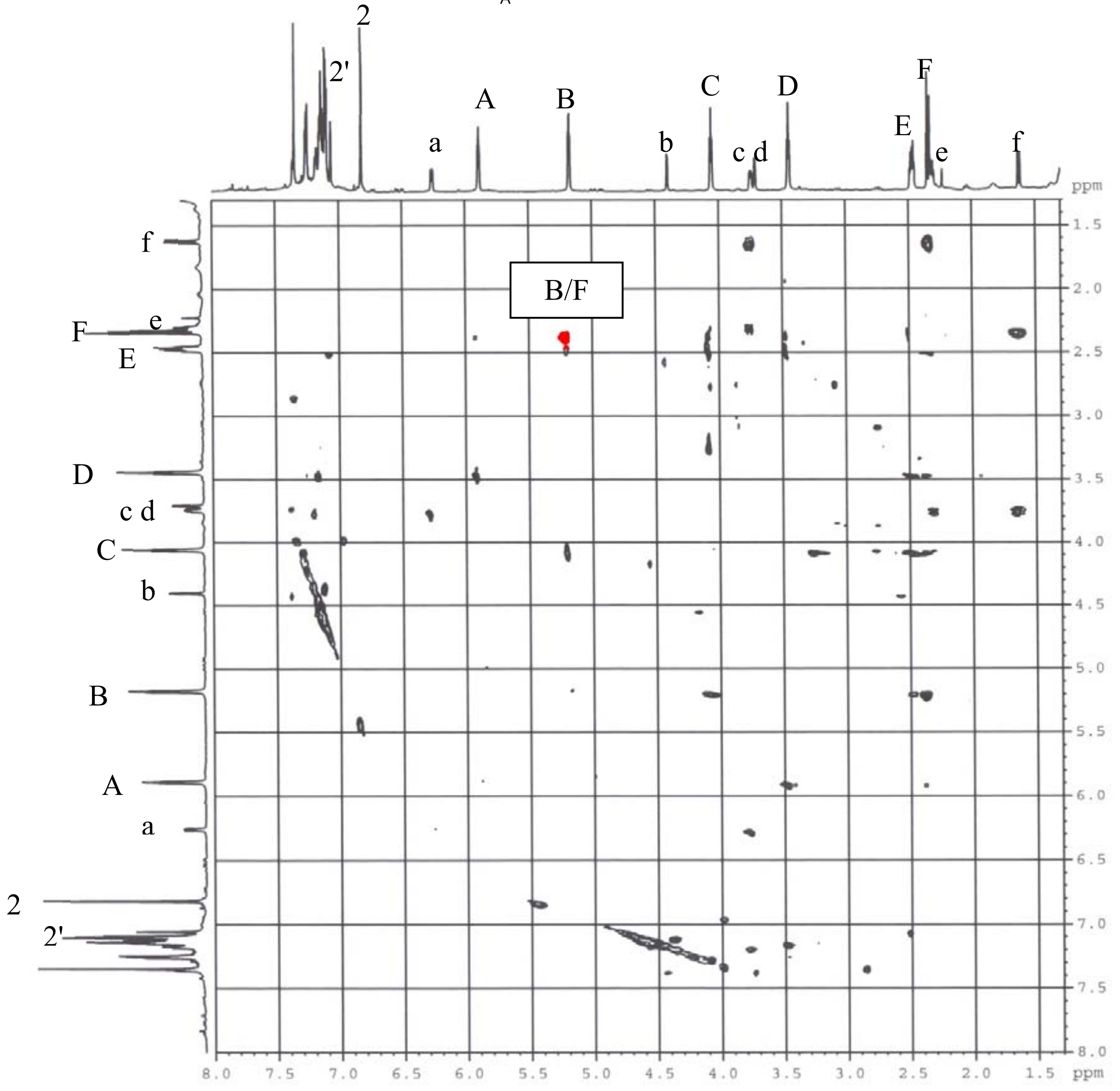

4-(1,2-Dihydronaphthalen-2-yl)oxazole (compound 9)

^{1}H NMR entire range spectrum of **9** (600 MHz, $CDCl_3$)

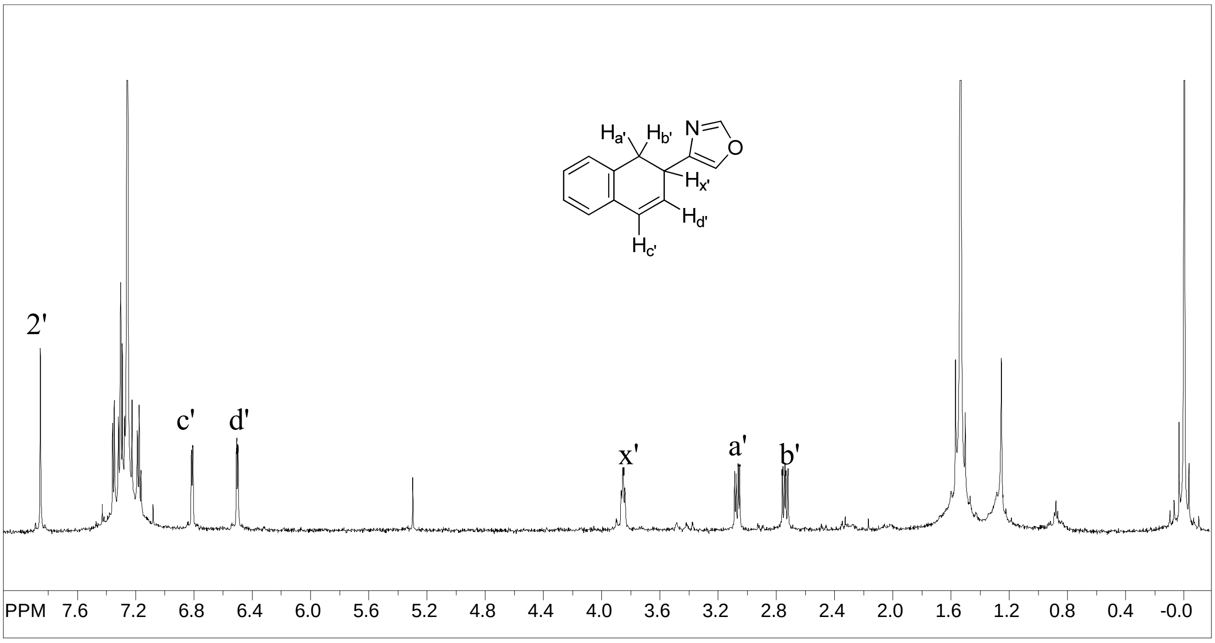

^{13}C NMR spectrum of **9** (3s, 9d, 1t) (150 MHz, $CDCl_3$)

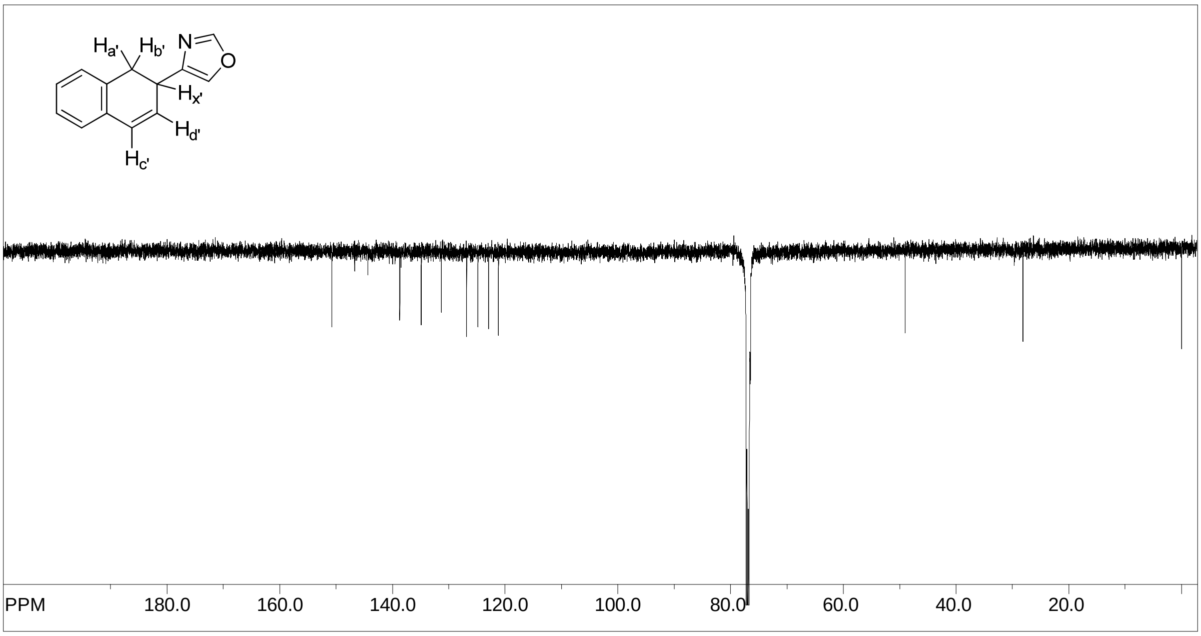

Irradiation mixture of 2 (compound *rel*-(2*S*)-10 with some quantity of **11)**

^{1}H NMR spectrum (600 MHz, $CDCl_3$)

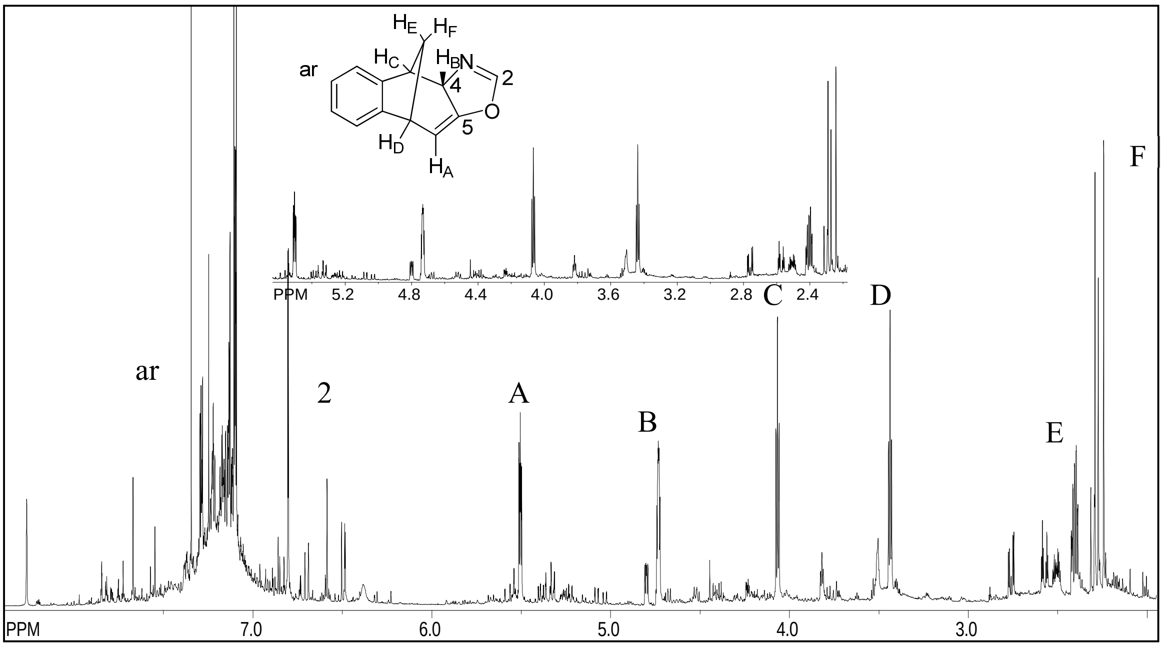

^{1}H NMR spectrum (600 MHz, C_6D_6)

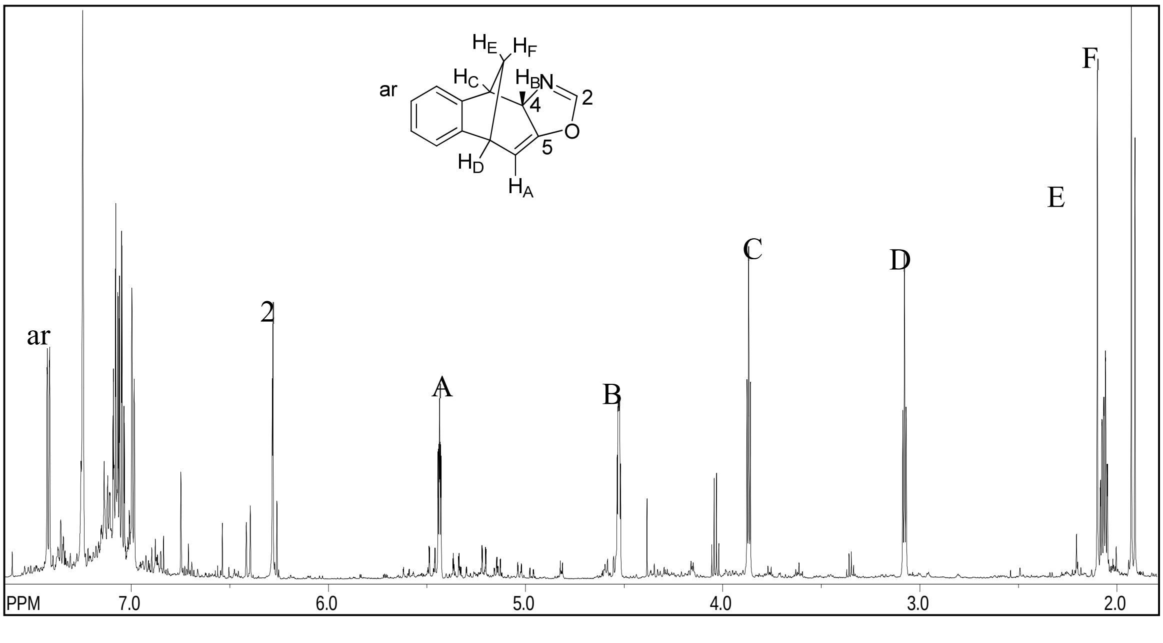

^{13}C NMR spectrum (3s, 9d, 1t) (150 MHz, $CDCl_3$)

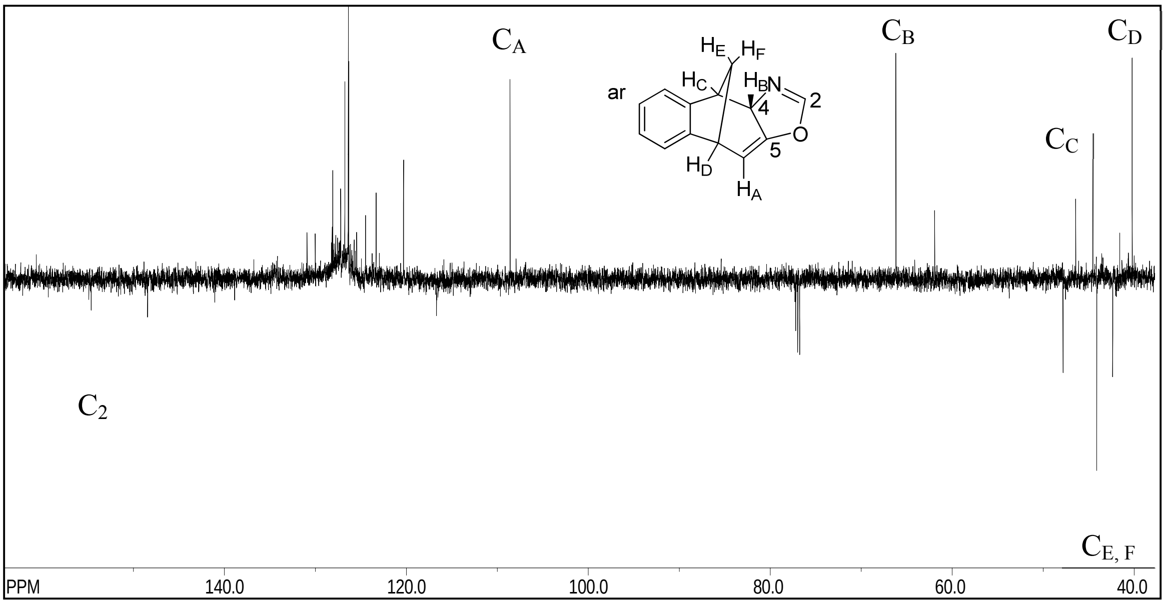

^{13}C NMR spectrum (3s, 9d, 1t) (150 MHz, C_6D_6)

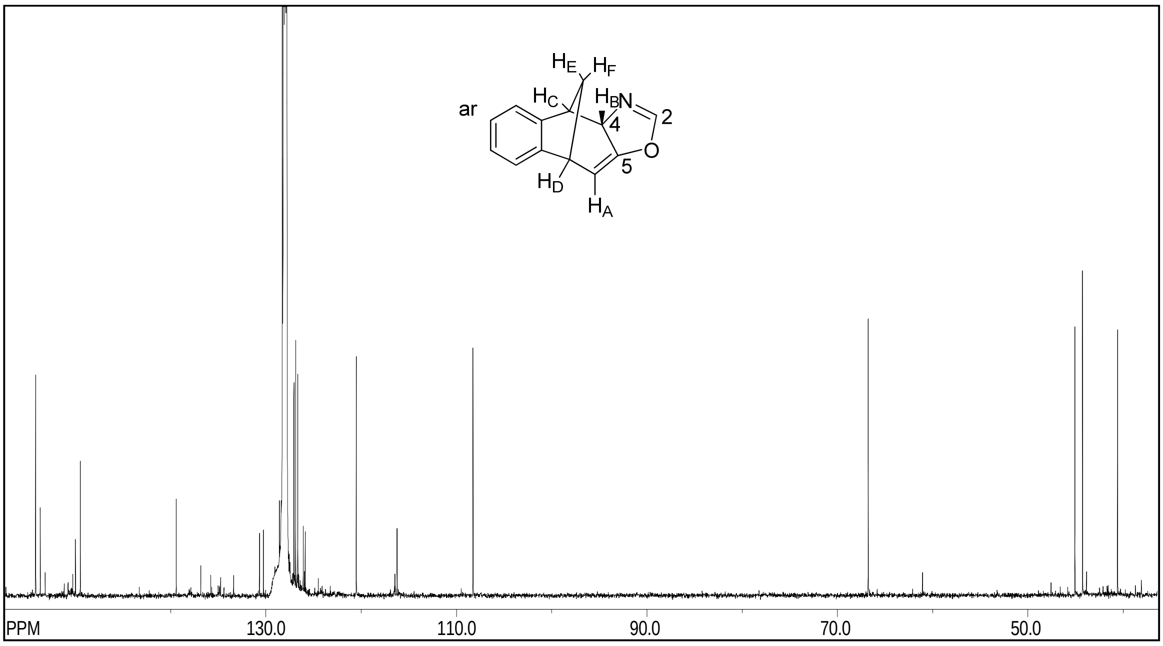

^{1}H NMR entire range spectrum (600 MHz, C_6D_6)

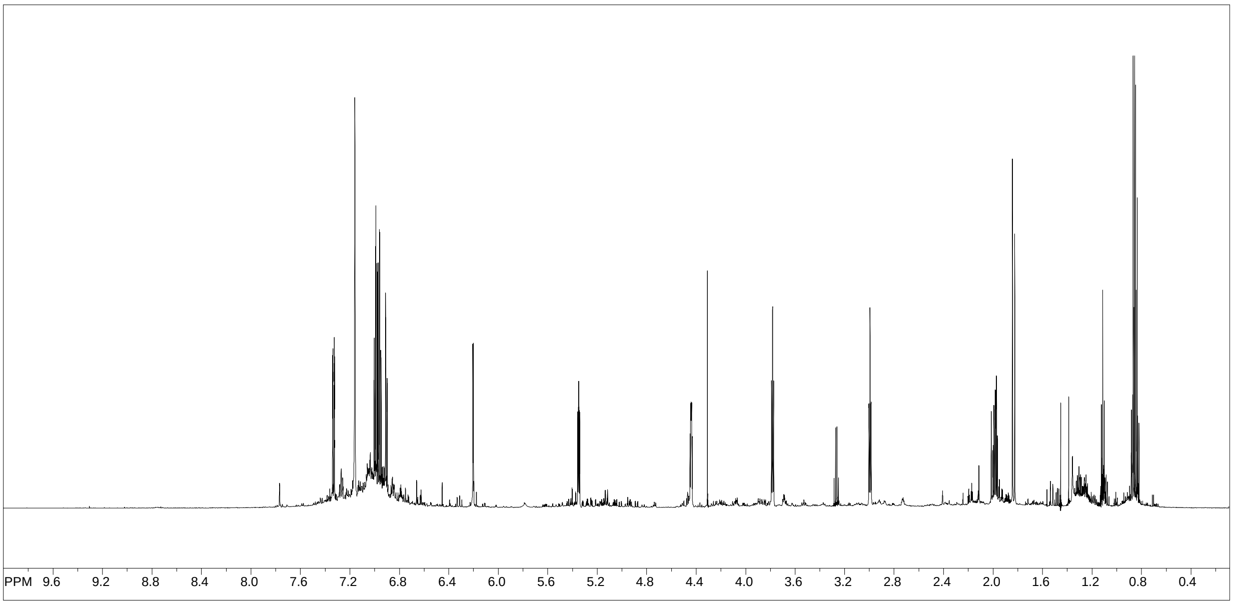

^{13}C NMR spectrum (3s, 9d, 1t) (150 MHz, C_6D_6)

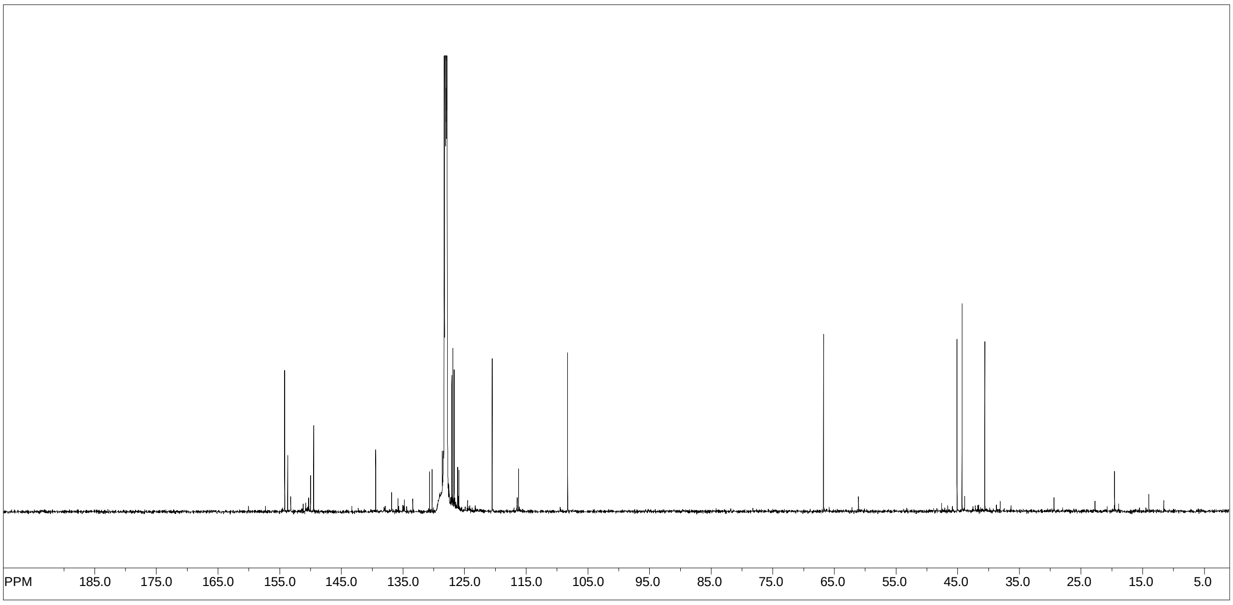

COSY spectrum (C_6D_6)

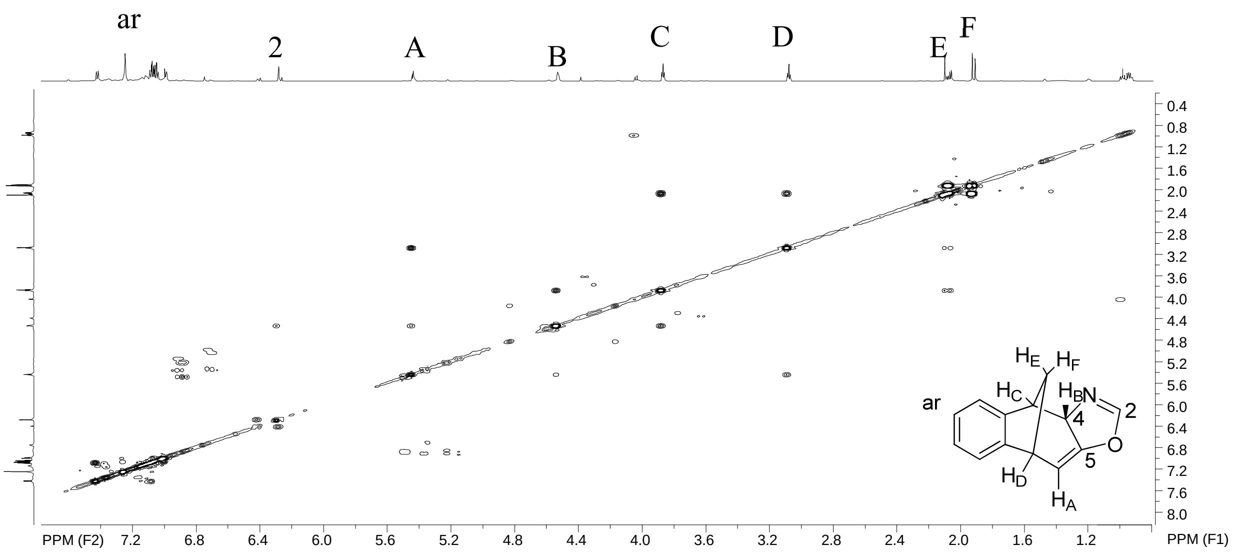

NOESY spectrum (C_6D_6) (**10**)

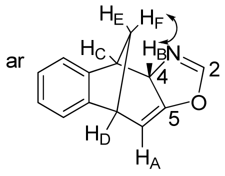

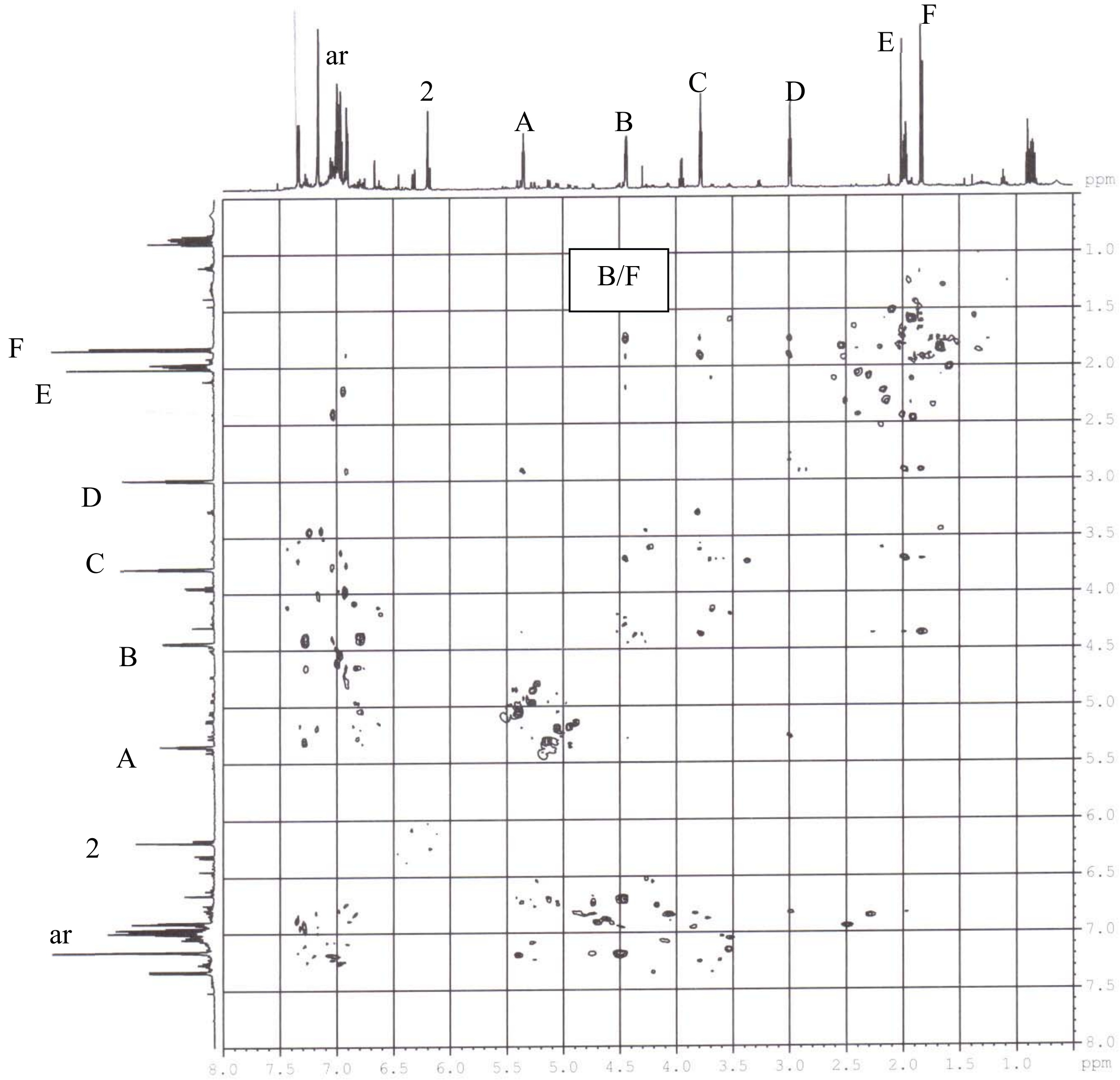

***N*-[*rel*-(9*S*)-10-Oxotricyclo[6.3.1.0^{2,7}]dodeca-2,4,6-trien-9-yl]formamide (compound *rel*-(9*S*)-11)**

^{1}H NMR spectrum (600 MHz, $CDCl_3$) (**11**)

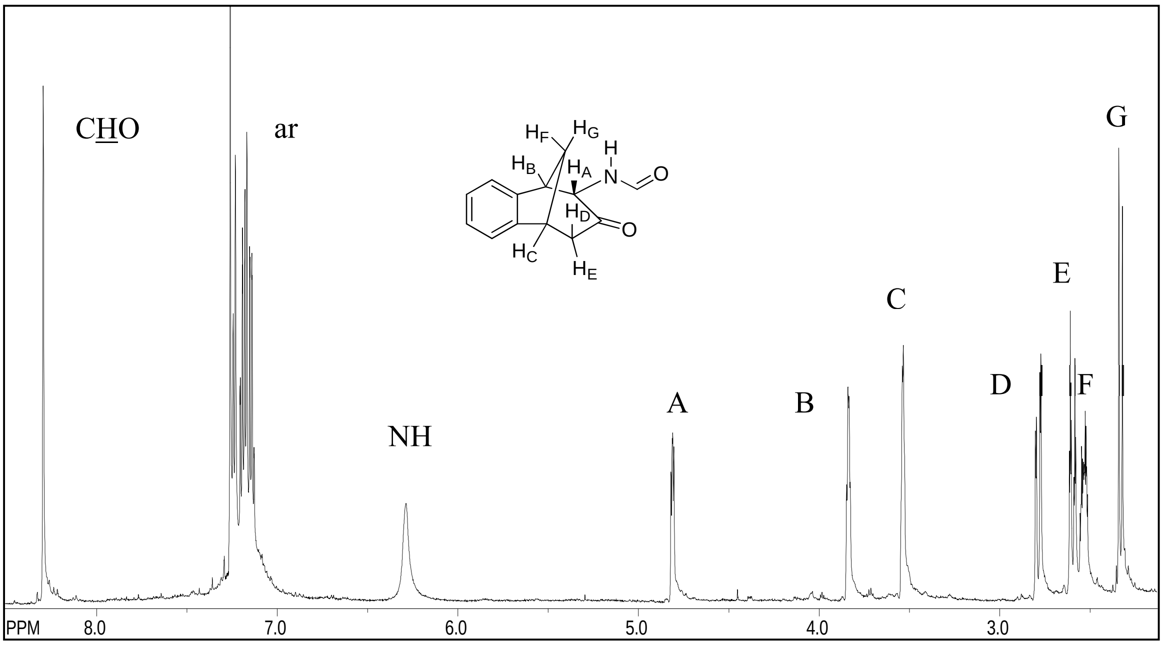

^{13}C NMR spectrum (3s, 8d, 2t) (150 MHz, $CDCl_3$) (**11**)

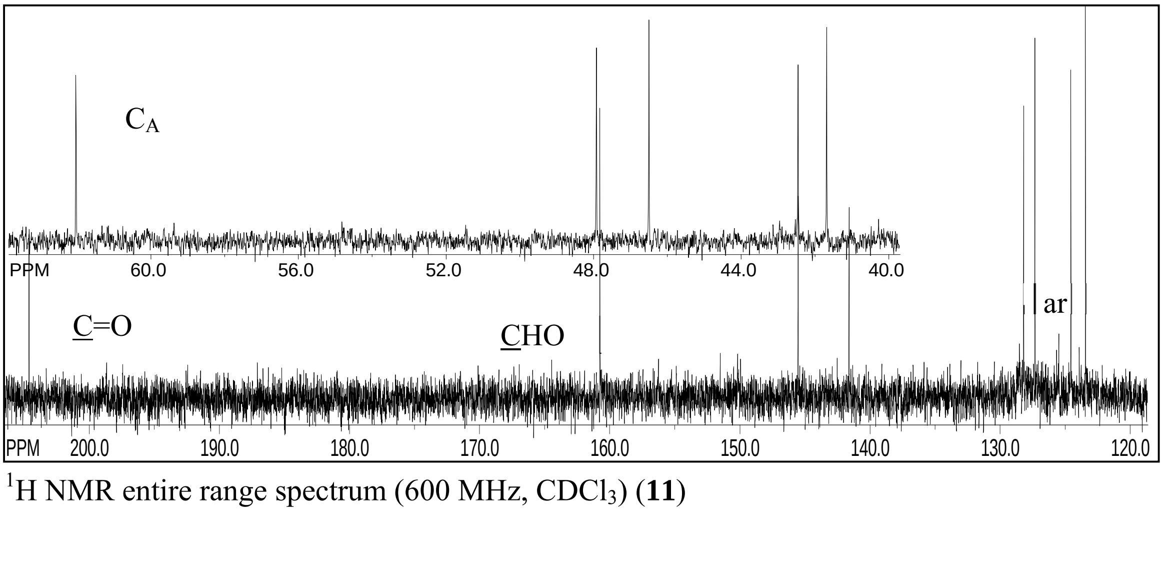

^{1}H NMR entire range spectrum (600 MHz, $CDCl_3$) (**11**)

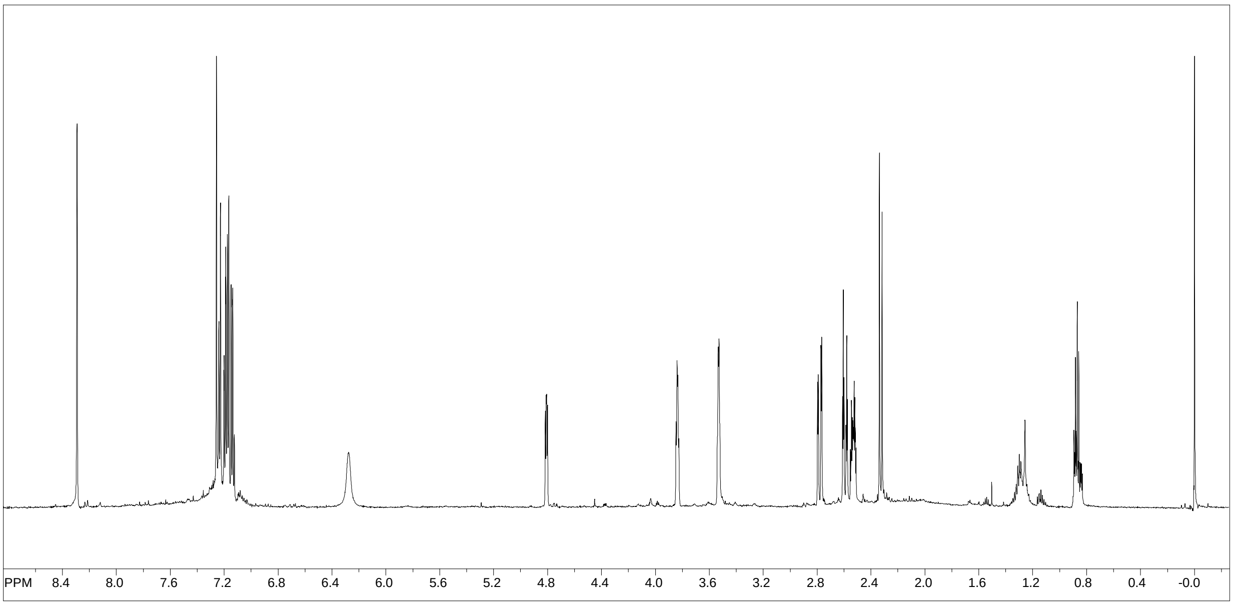

^{13}C NMR entire range spectrum (3s, 8d, 2t) (150 MHz, $CDCl_3$) (**11**)

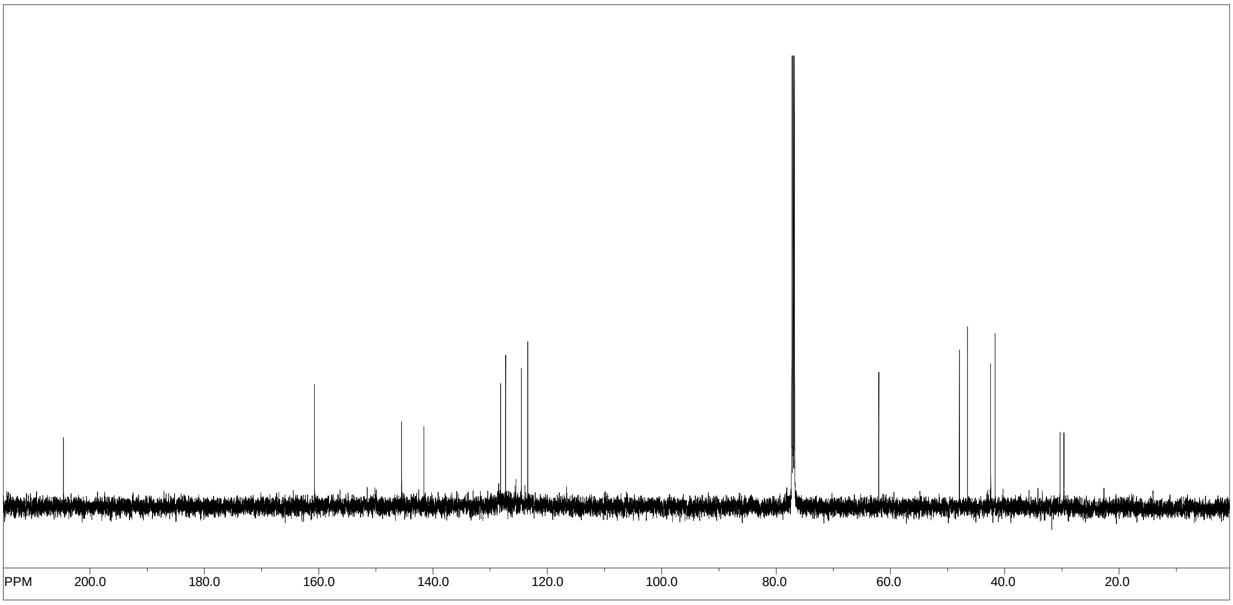

COSY spectrum ($CDCl_3$) (**11**)

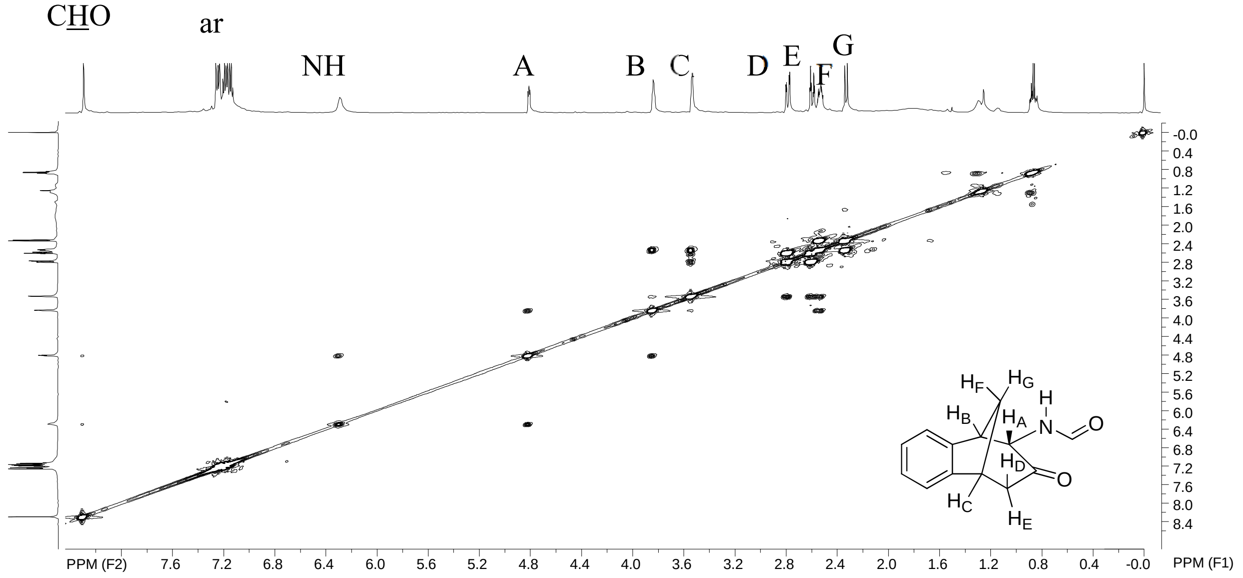

HSQC spectrum ($CDCl_3$) (**11**)

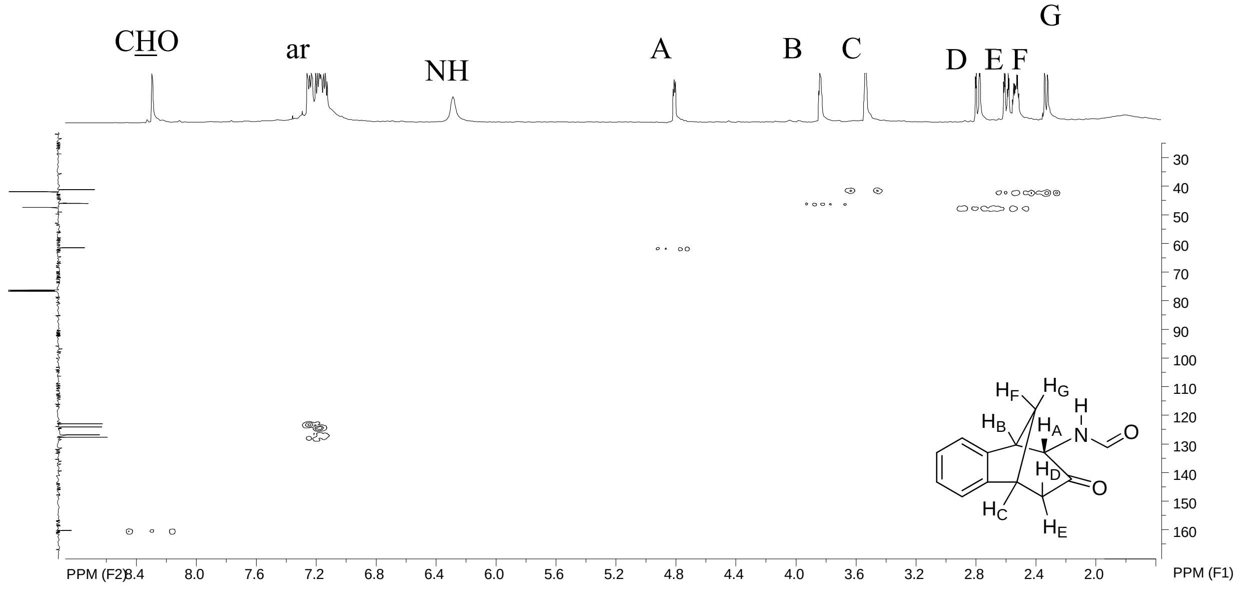

NOESY spectrum ($CDCl_3$) (**11**)

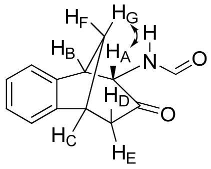

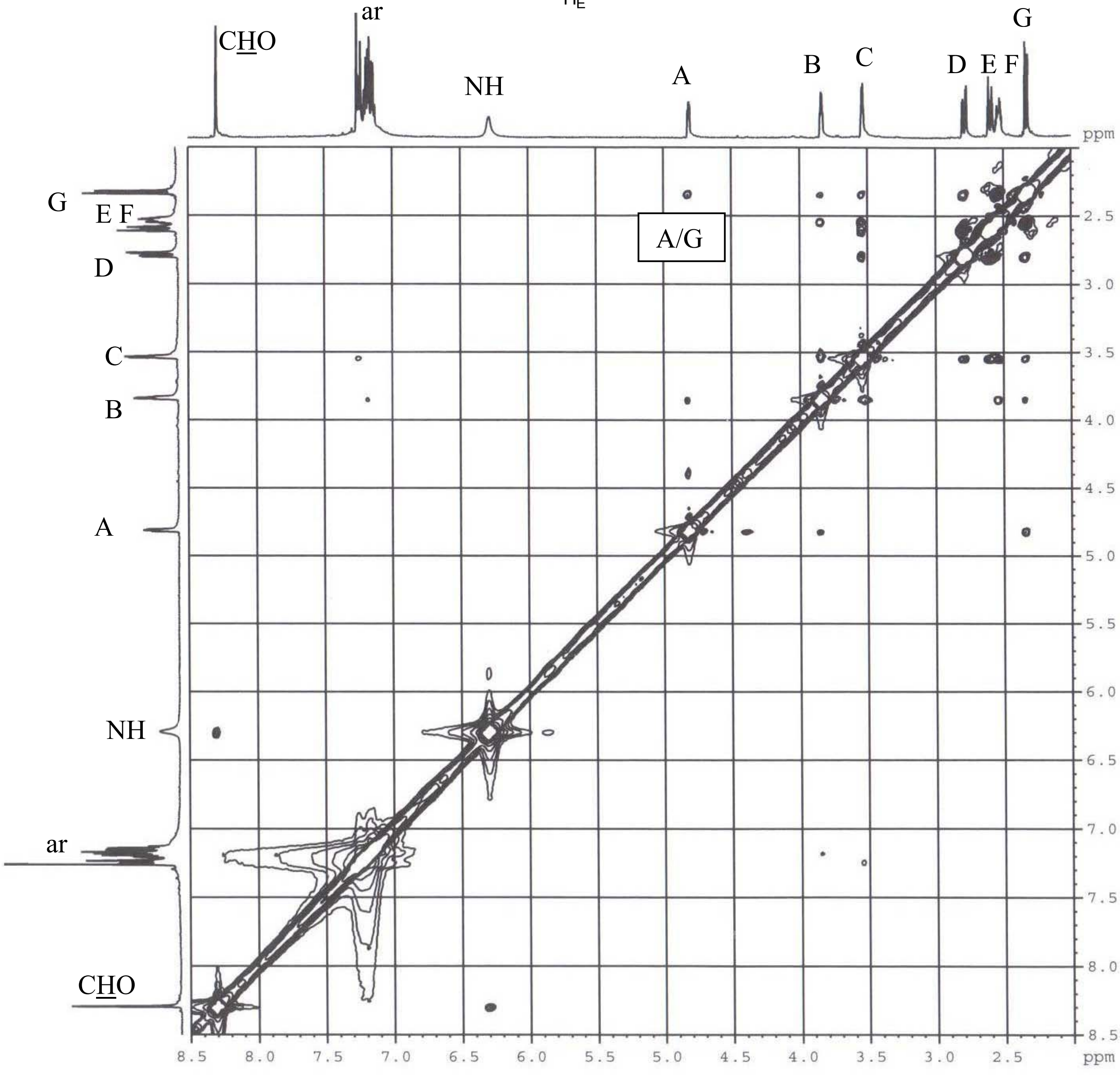

IR spectrum (**11**)

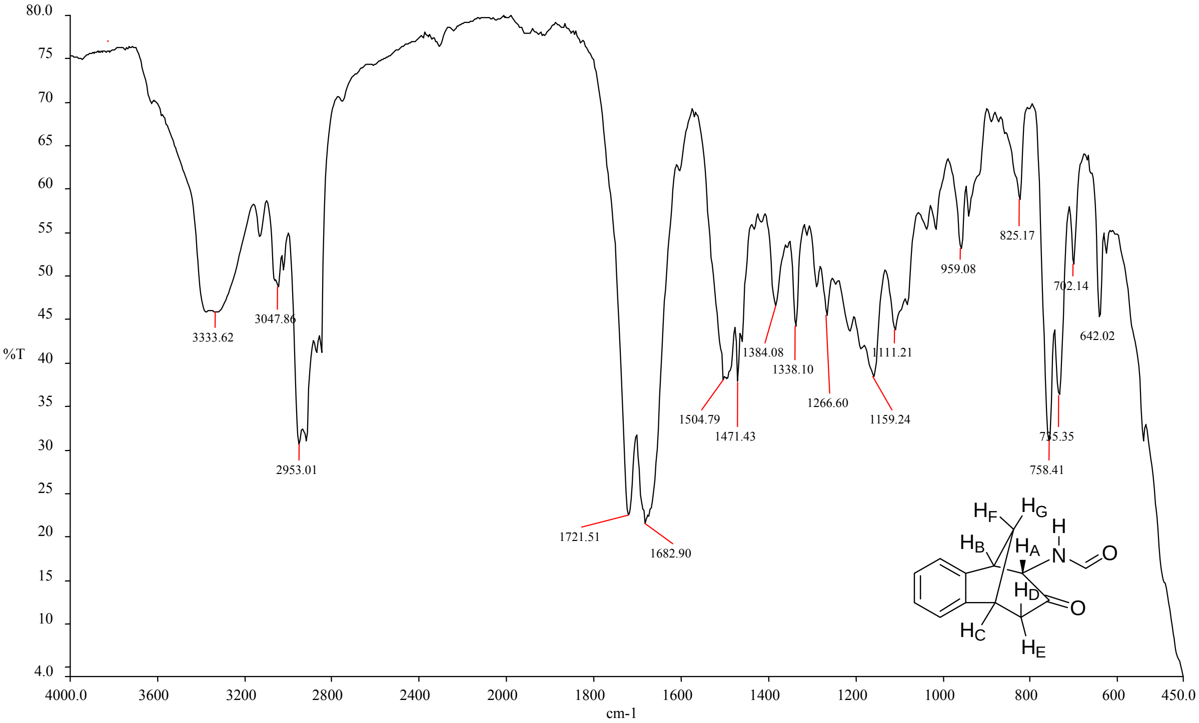

***rel*-(9*S*)-10-Oxotricyclo[6.3.1.0^{2,7}]dodeca-2,4,6-trien-9-yl formiate (compound *rel*-(2*S*)-12a)**

^{1}H NMR spectrum (600 MHz, $CDCl_3$) (**12a**)

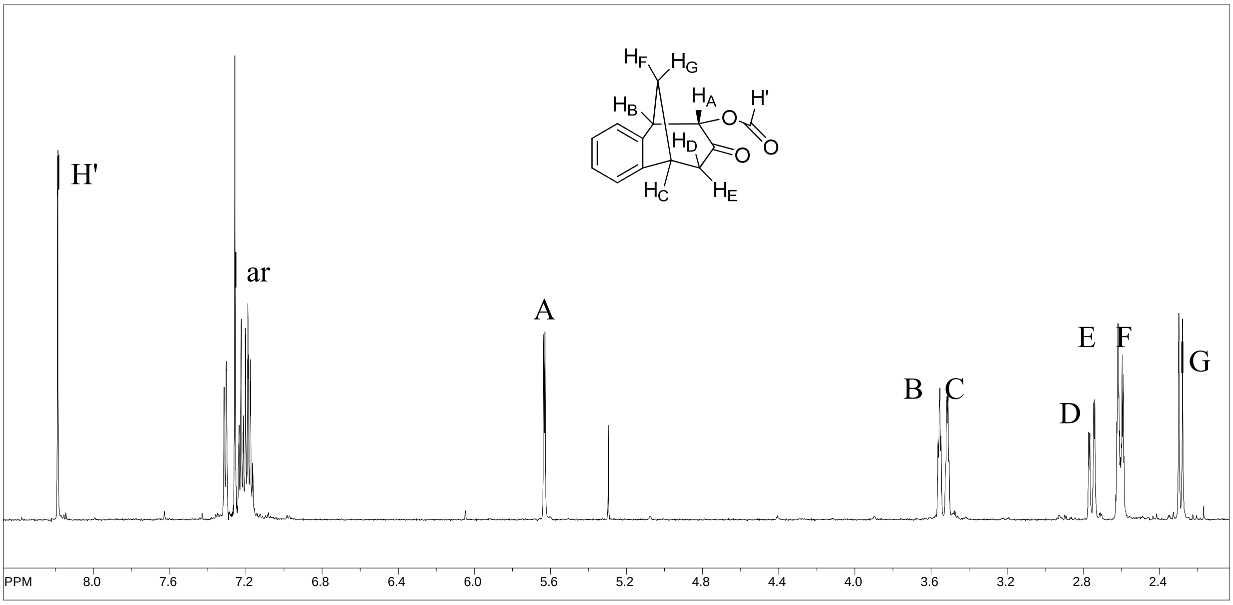

^{13}C NMR spectrum (150 MHz, 3s, 8d, 2t) ($CDCl_3$) (**12a**)

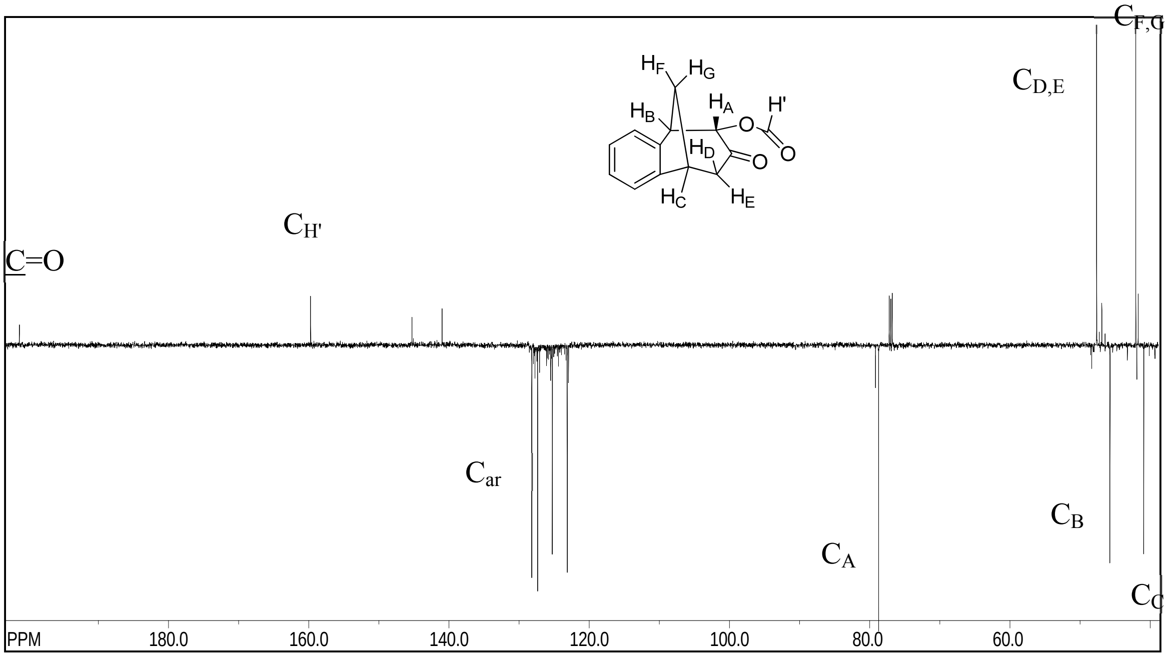

50S

^{1}H NMR entire range spectrum (600 MHz, $CDCl_3$) (**12a**)

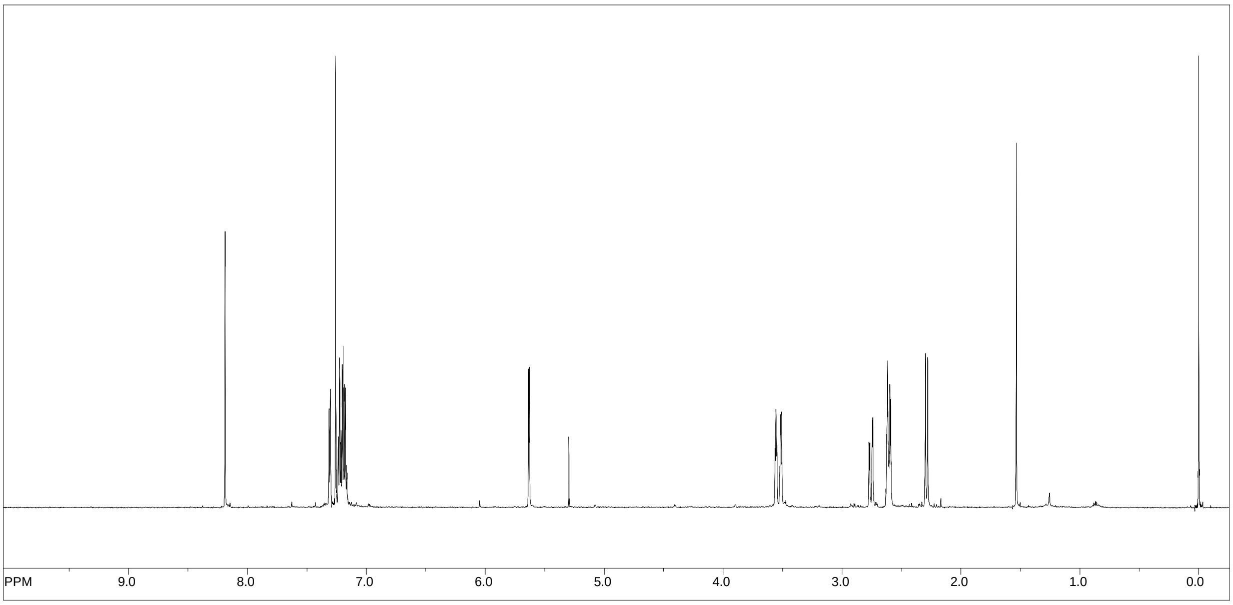

^{13}C NMR entire range spectrum (150 MHz, 3s, 8d, 2t) ($CDCl_3$) (**12a**)

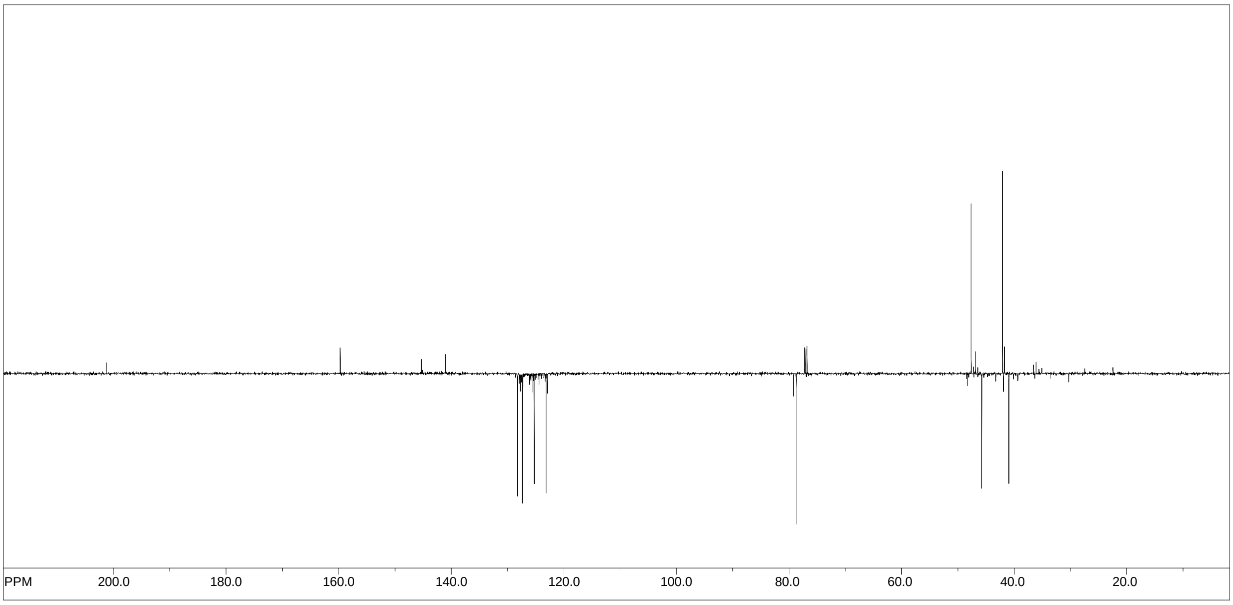

COSY spectrum ($CDCl_3$) (**12a**)

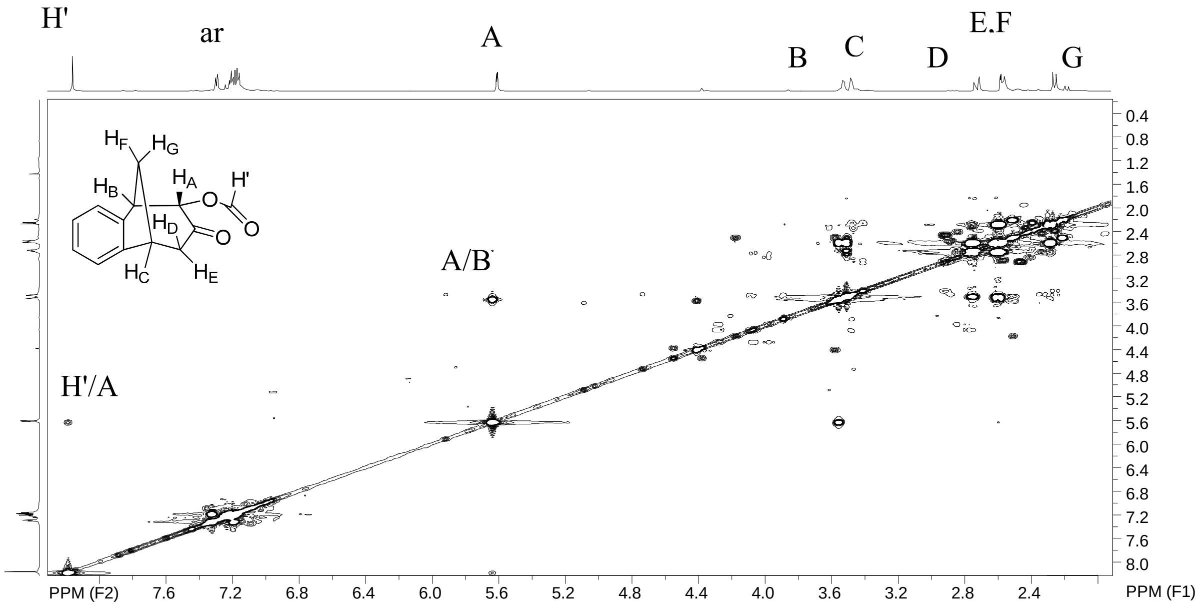

HSQC spectrum ($CDCl_3$) (**12a**)

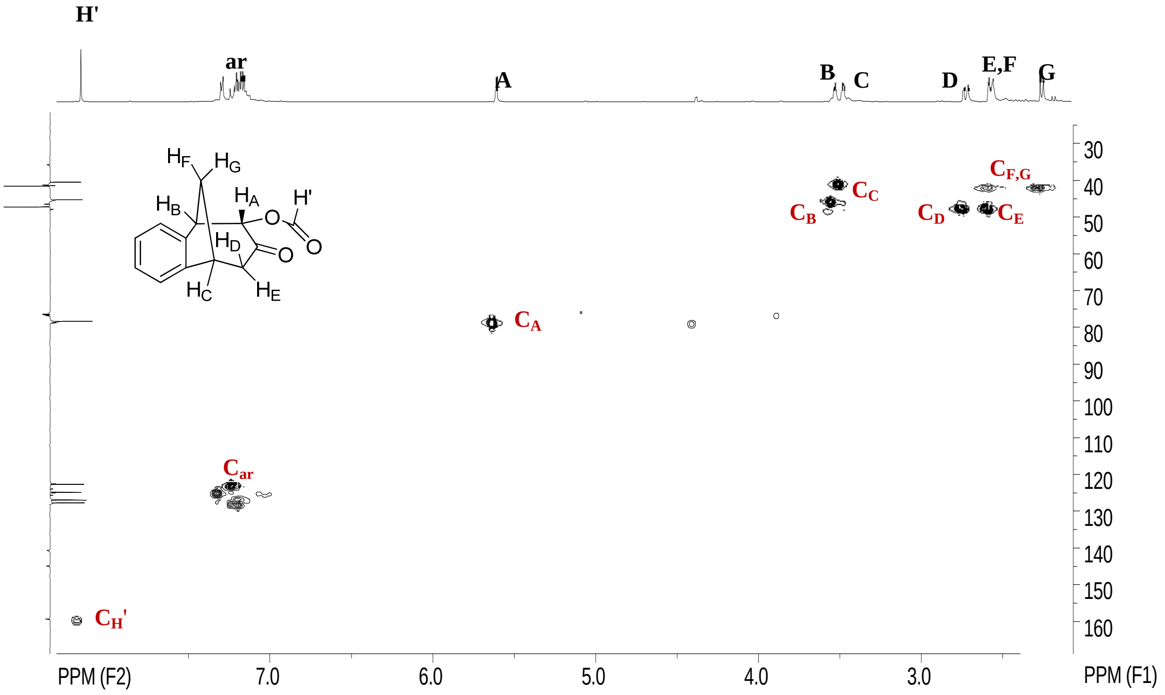

NOESY spectrum ($CDCl_3$) (**12a**)

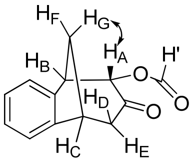

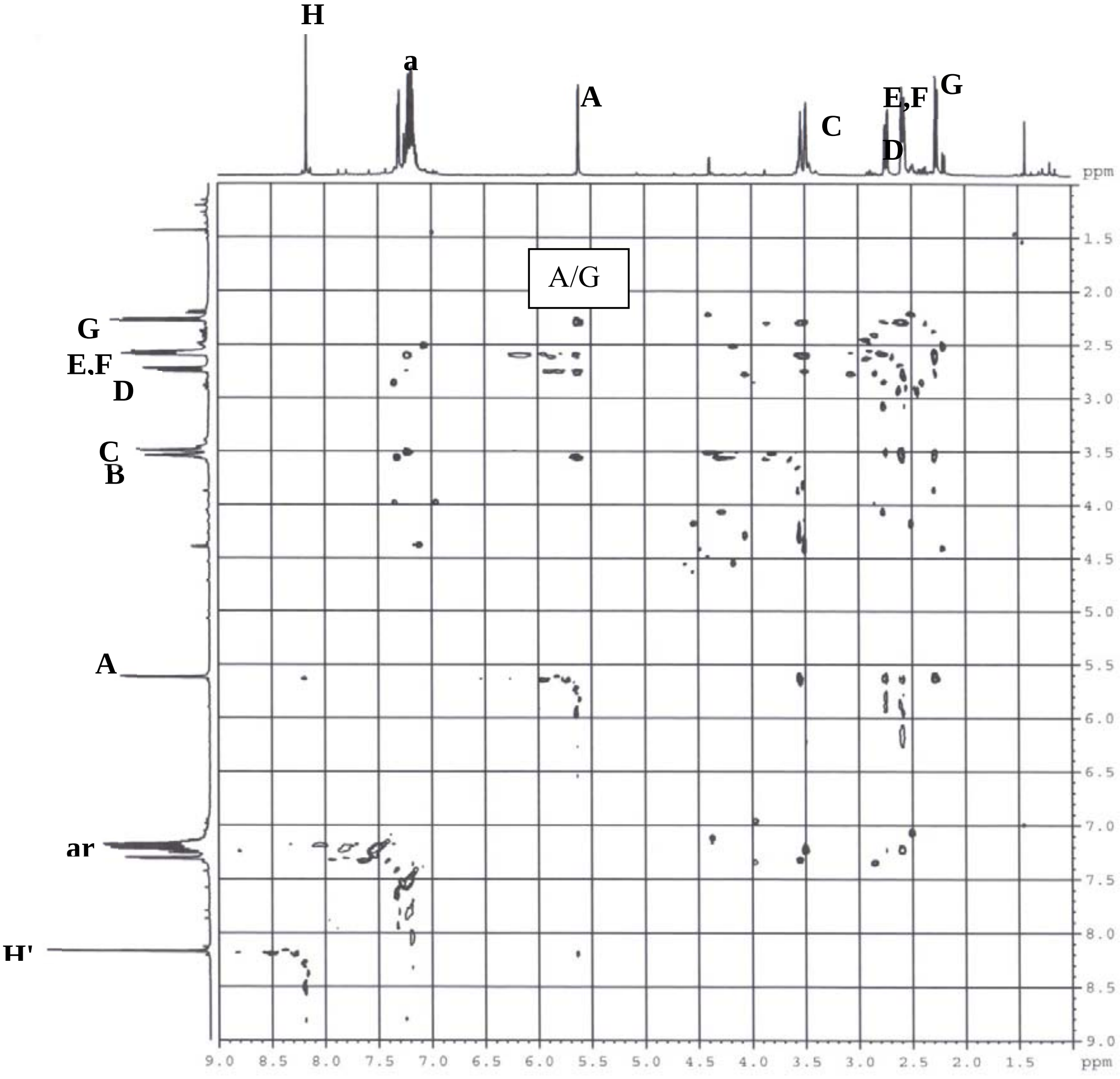

IR spectrum (**12a**)

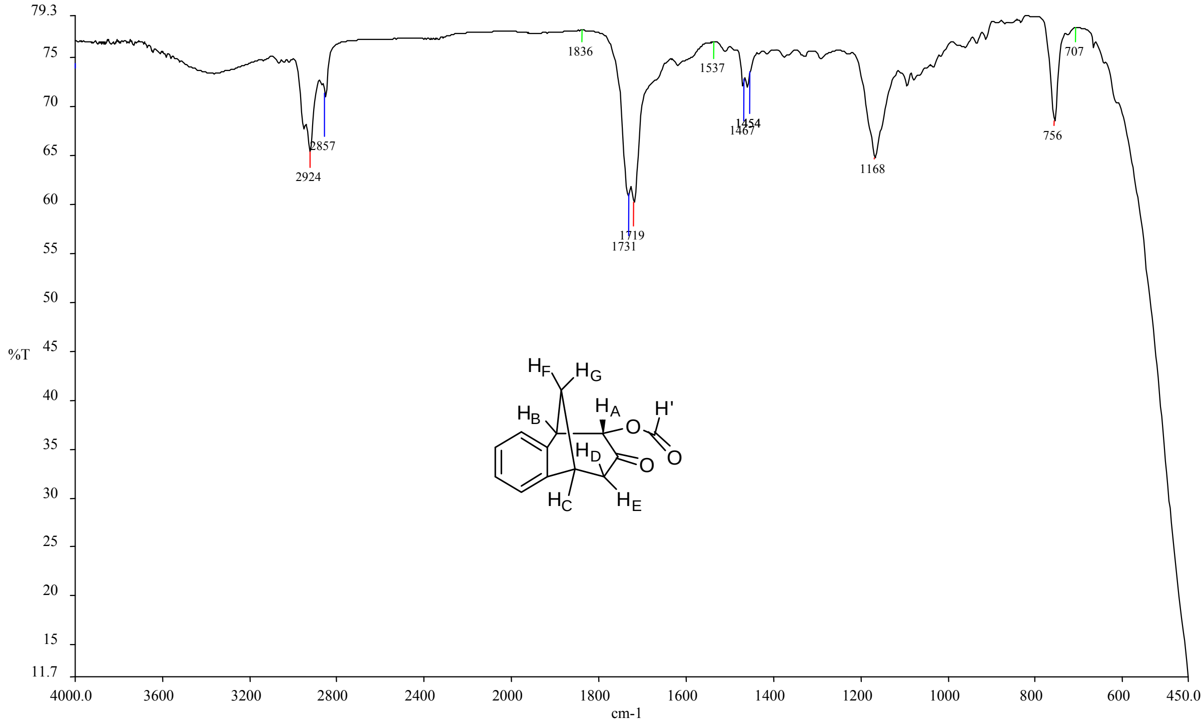

***rel*-(9*R*)-10-Oxotricyclo[6.3.1.0^{2,7}]dodeca-2,4,6-trien-9-yl formiate (compound *rel*-(9*R*)-12b)**

^{1}H NMR spectrum (600 MHz, $CDCl_3$) (**12b**)

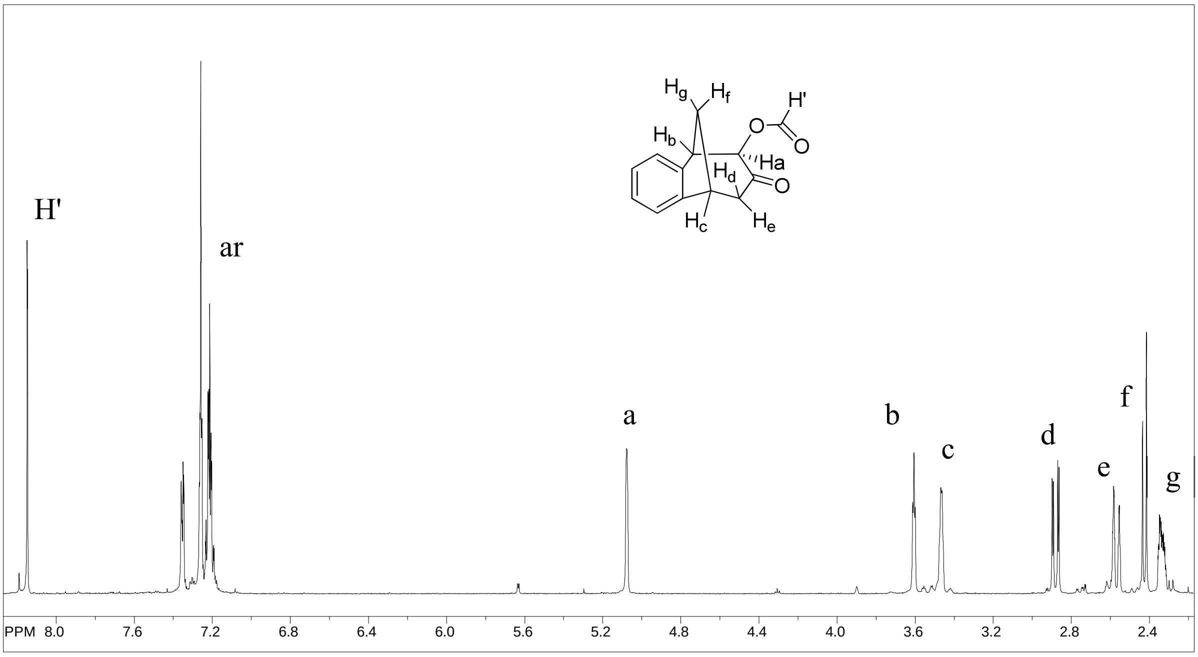

^{13}C NMR spectrum (150 MHz, 3s, 8d, 2t) ($CDCl_3$) (**12b**)

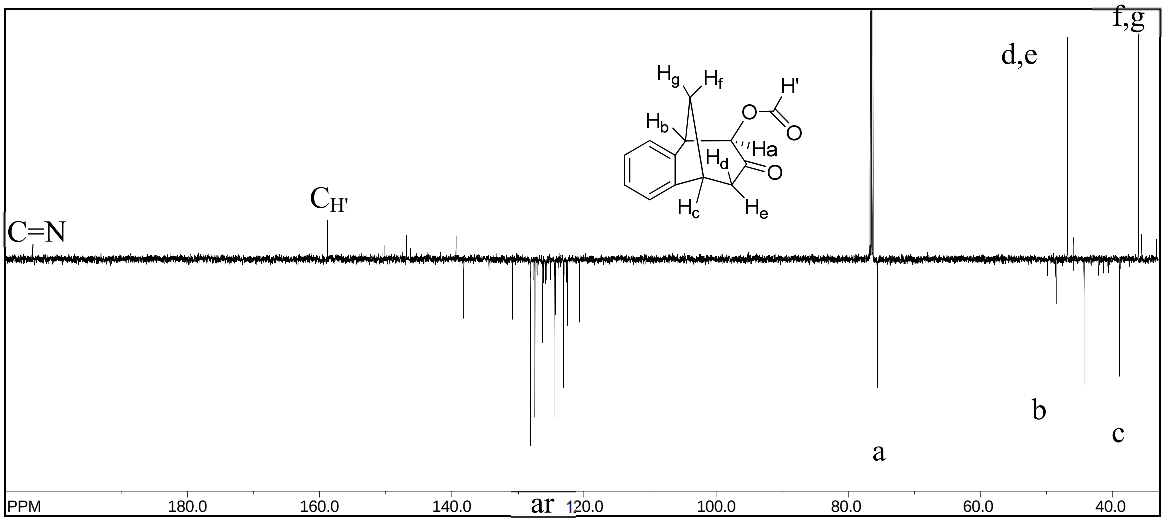

^{1}H NMR entire range spectrum (600 MHz, $CDCl_3$) (**12b**)

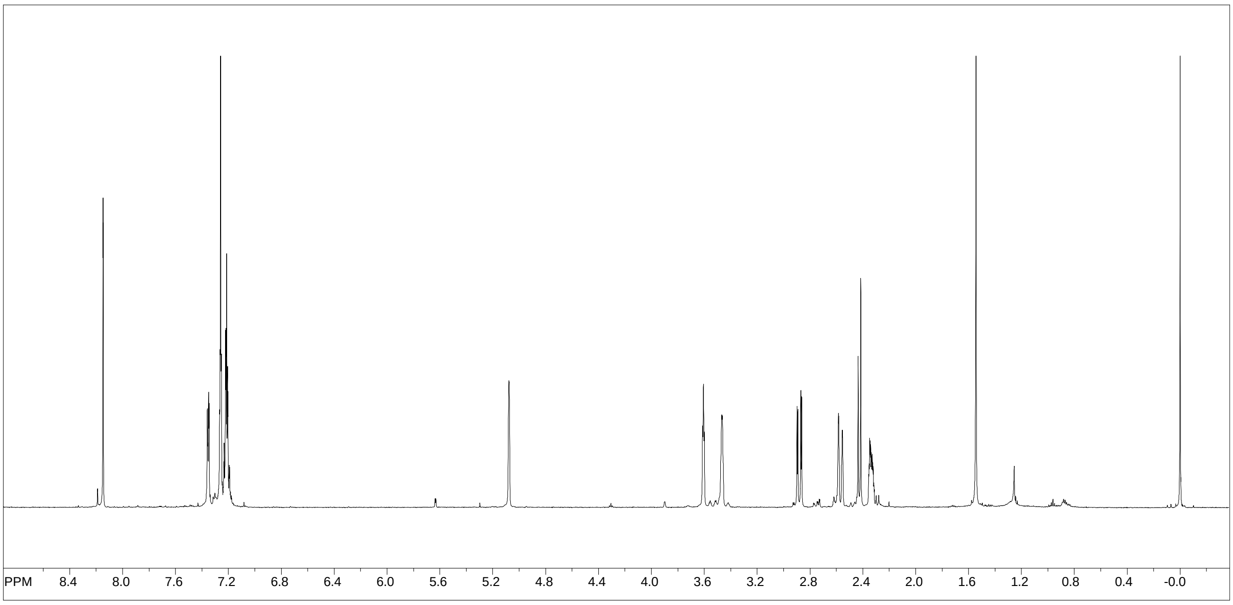

^{13}C NMR entire range spectrum (150 MHz, 3s, 8d, 2t) ($CDCl_3$) (**12b**)

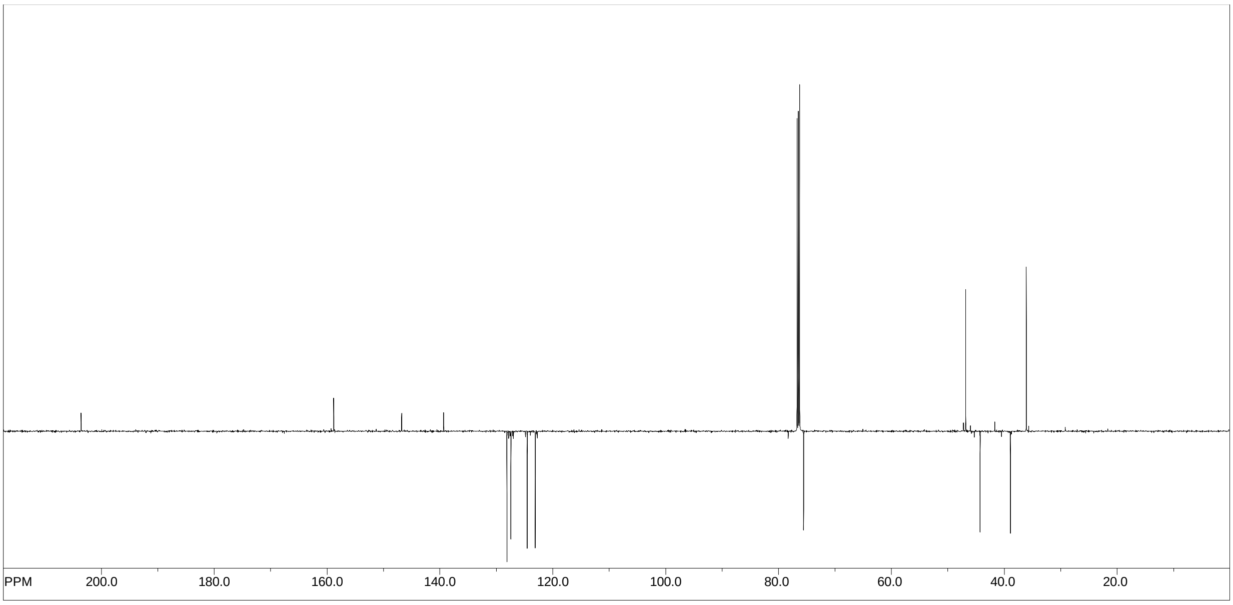

COSY spectrum ($CDCl_3$) (**12b**)

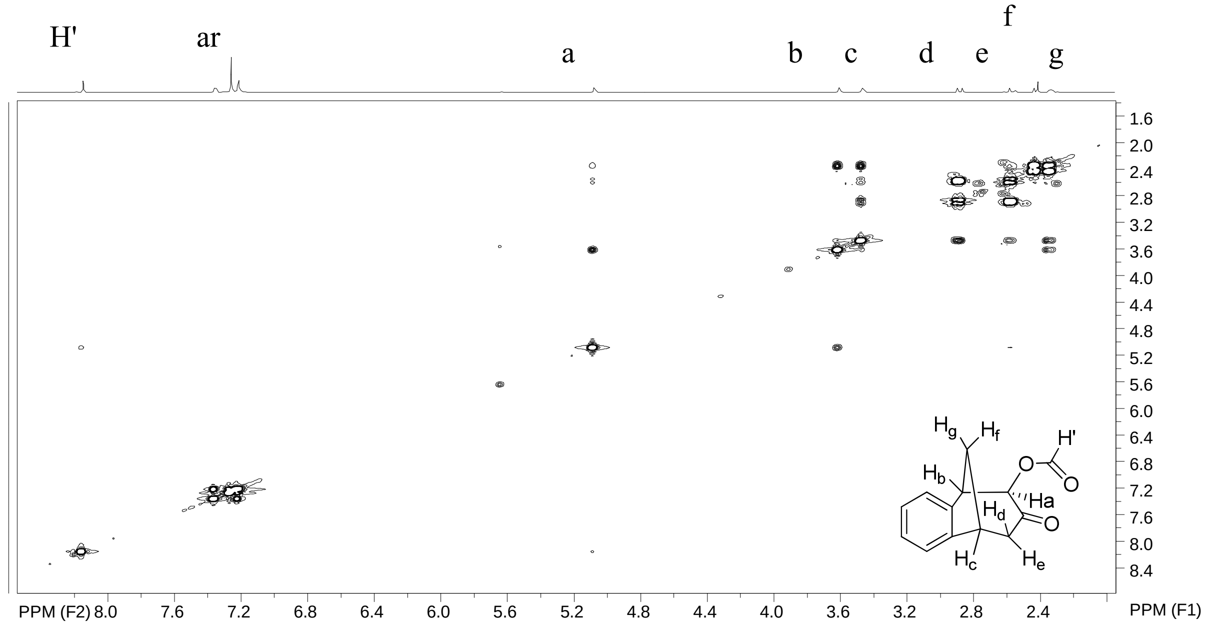

HSQC spectrum ($CDCl_3$) (**12b**)

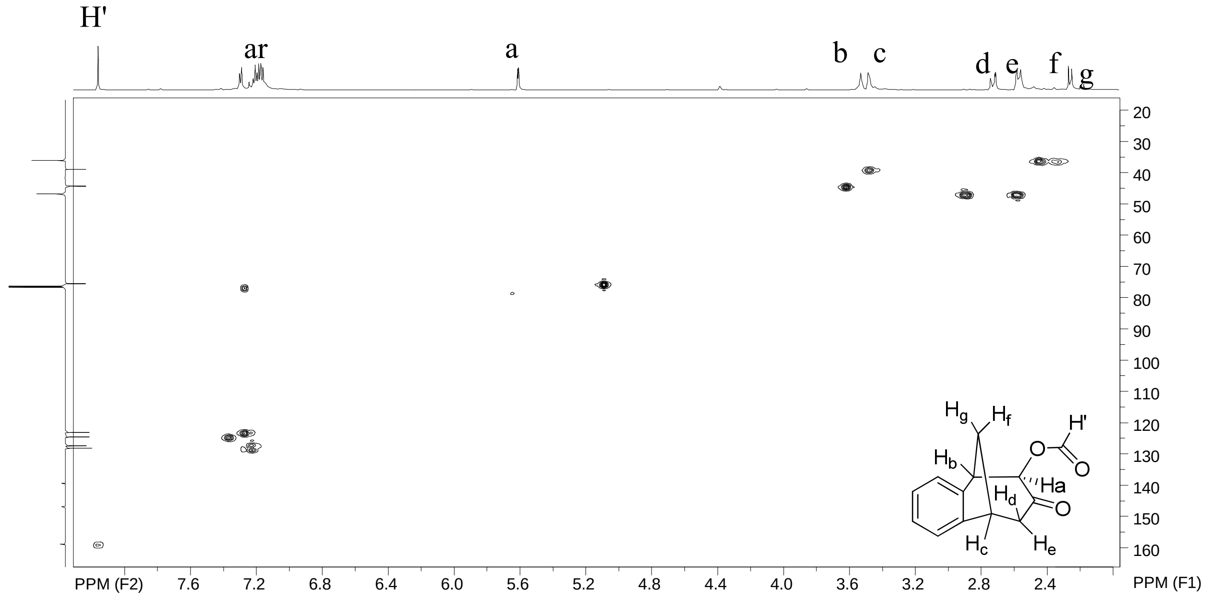

NOESY spectrum ($CDCl_3$) (**12b**)

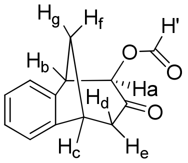

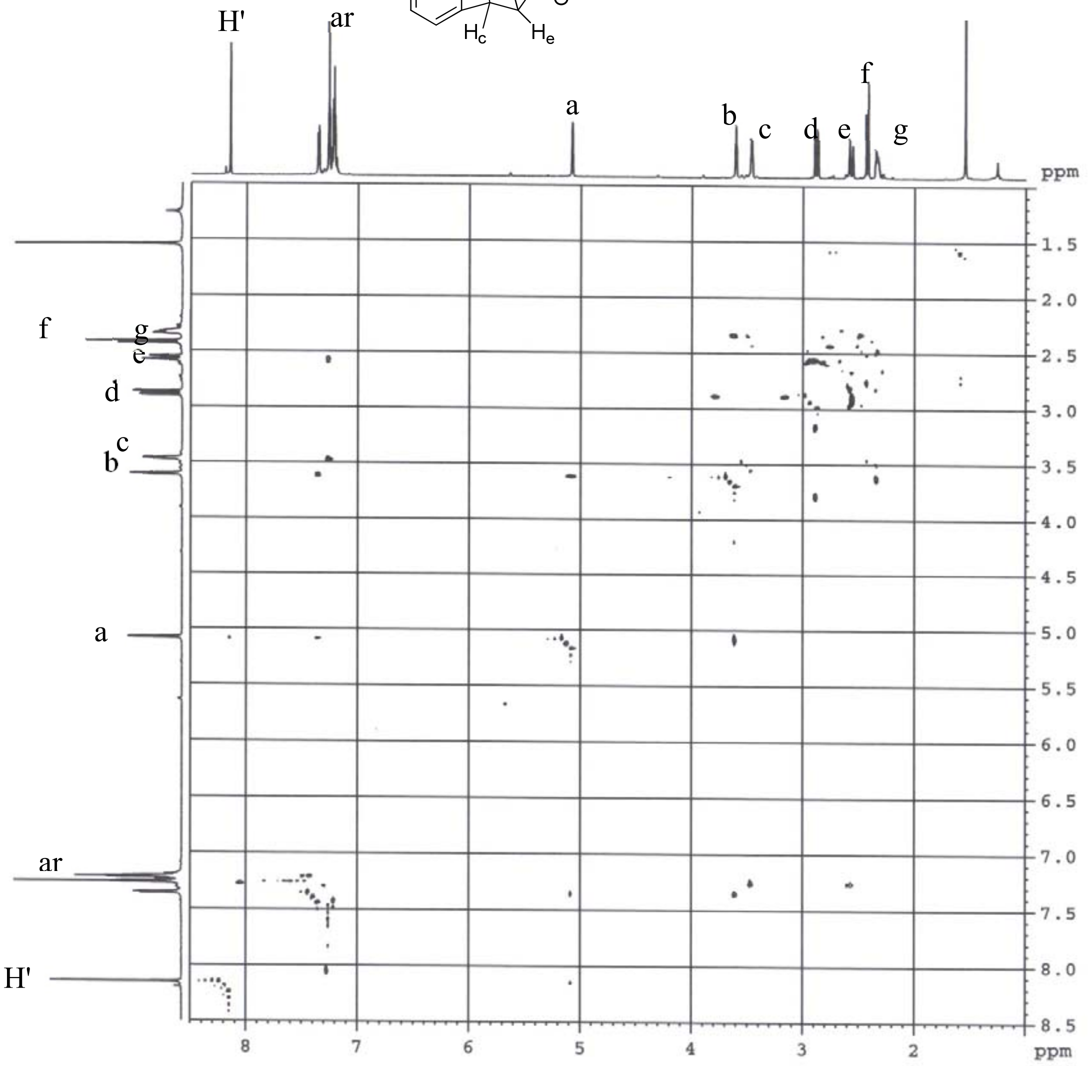

***rel*-(2*S*)-4-Ethoxy-3-oxa-5-azatetracyclo[6.6.1.0^{2,6}.0^{9,14}]pentadeca-5,9,11,13-tetraen (compound *rel*-(2*S*)-13)**

^{1}H NMR spectrum (600 MHz, $CDCl_3$) (**13**)

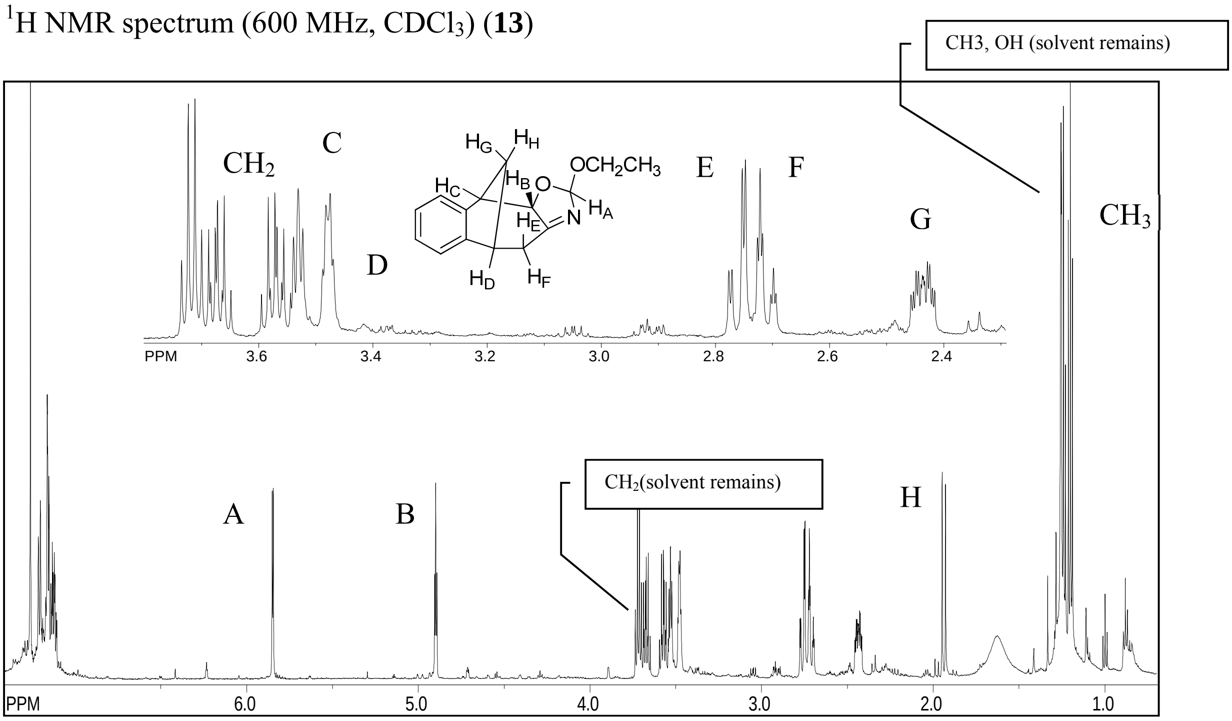

^{13}C NMR spectrum (150 MHz, 3s, 8d, 3t, 1q) ($CDCl_3$) (**13**)

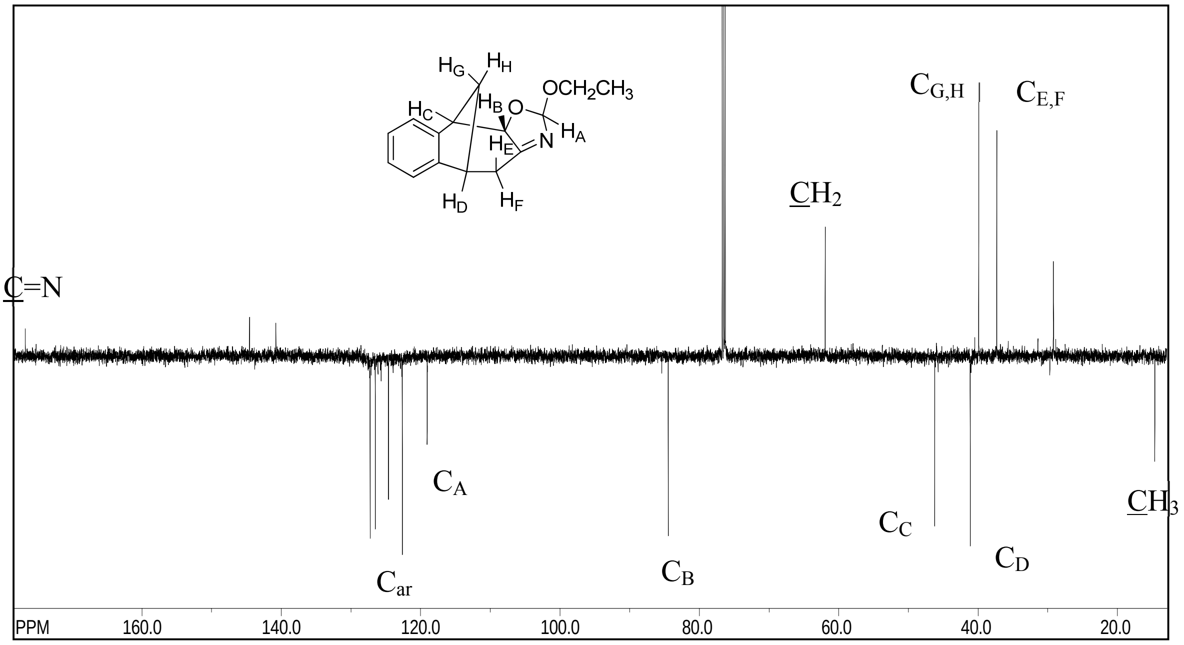

^{1}H NMR entire range spectrum (600 MHz, $CDCl_3$) (**13**)

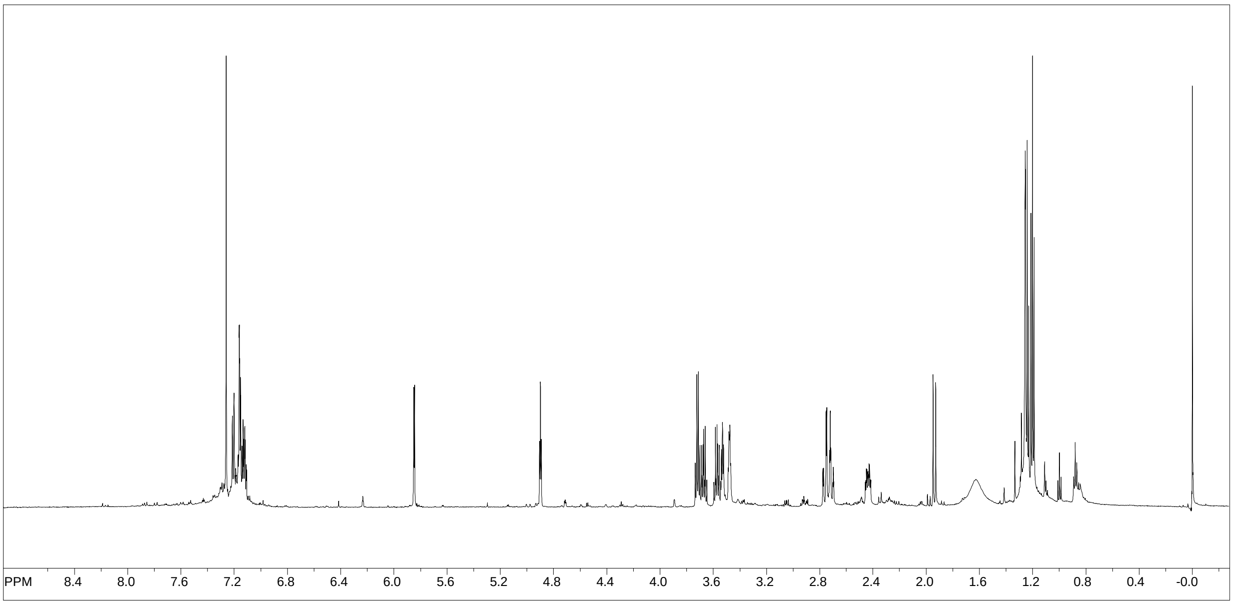

^{13}C NMR entire range spectrum (150 MHz, 3s, 8d, 3t, 1q) ($CDCl_3$) (**13**)

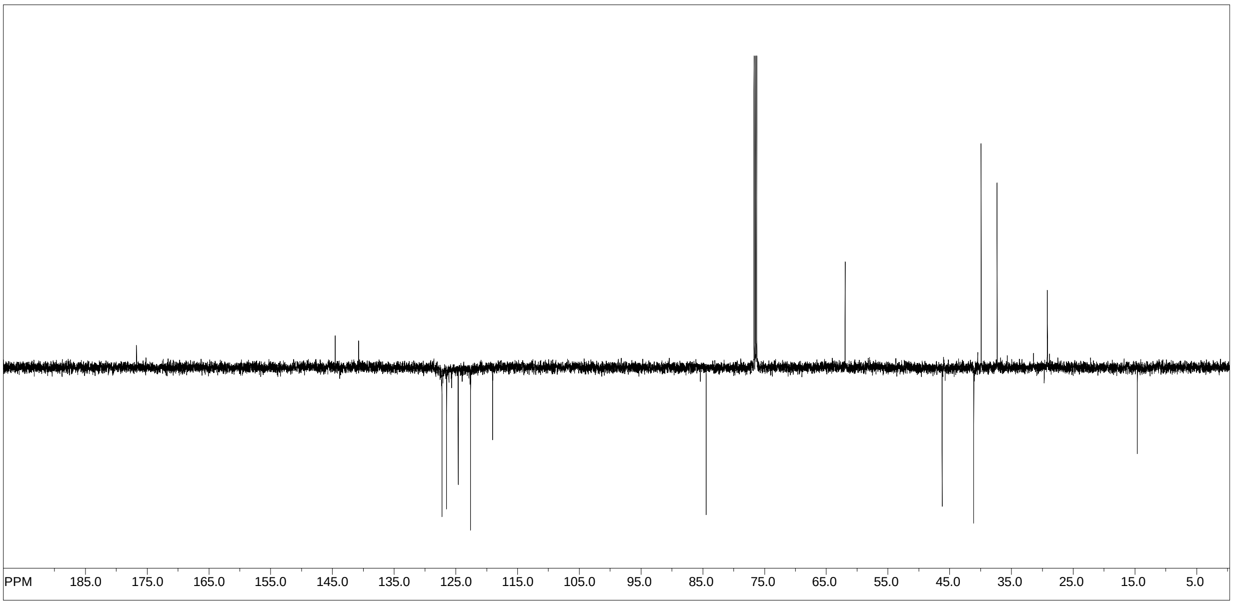

COSY spectrum ($CDCl_3$) (**13**)

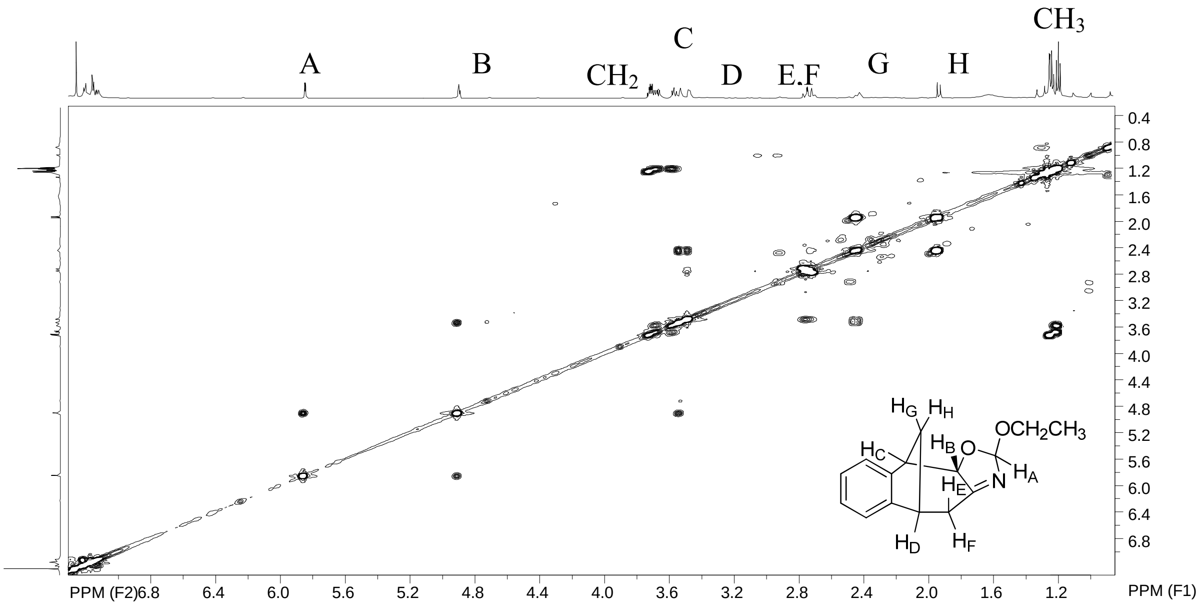

HSQC spectrum ($CDCl_3$) (**13**)

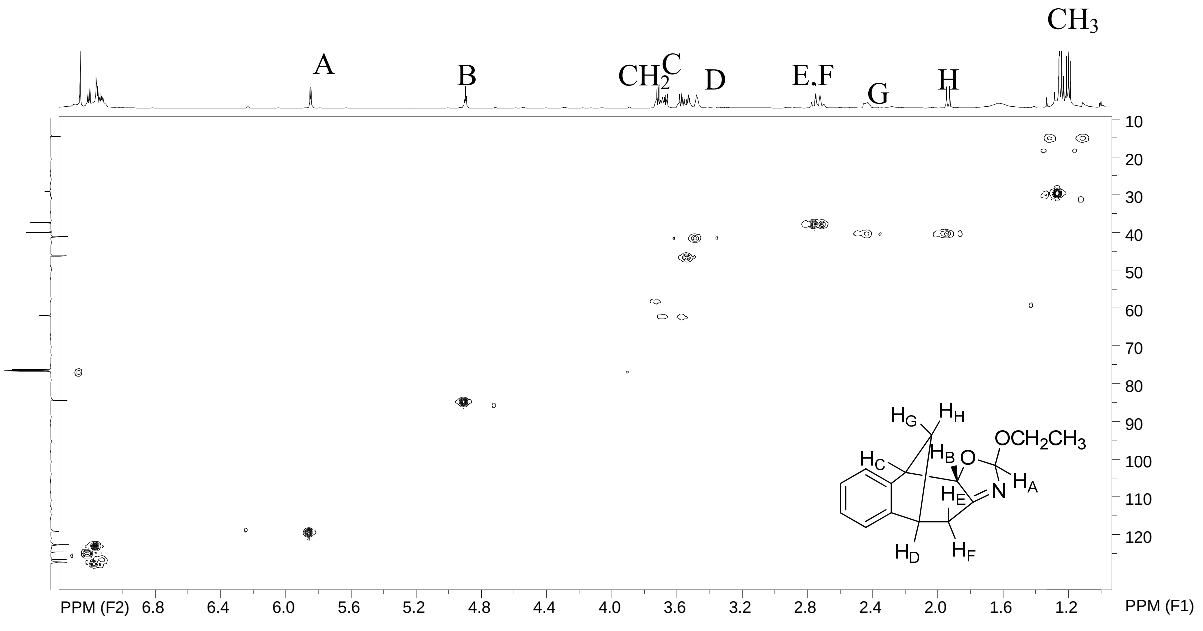

NOESY spectrum ($CDCl_3$) (**13**)

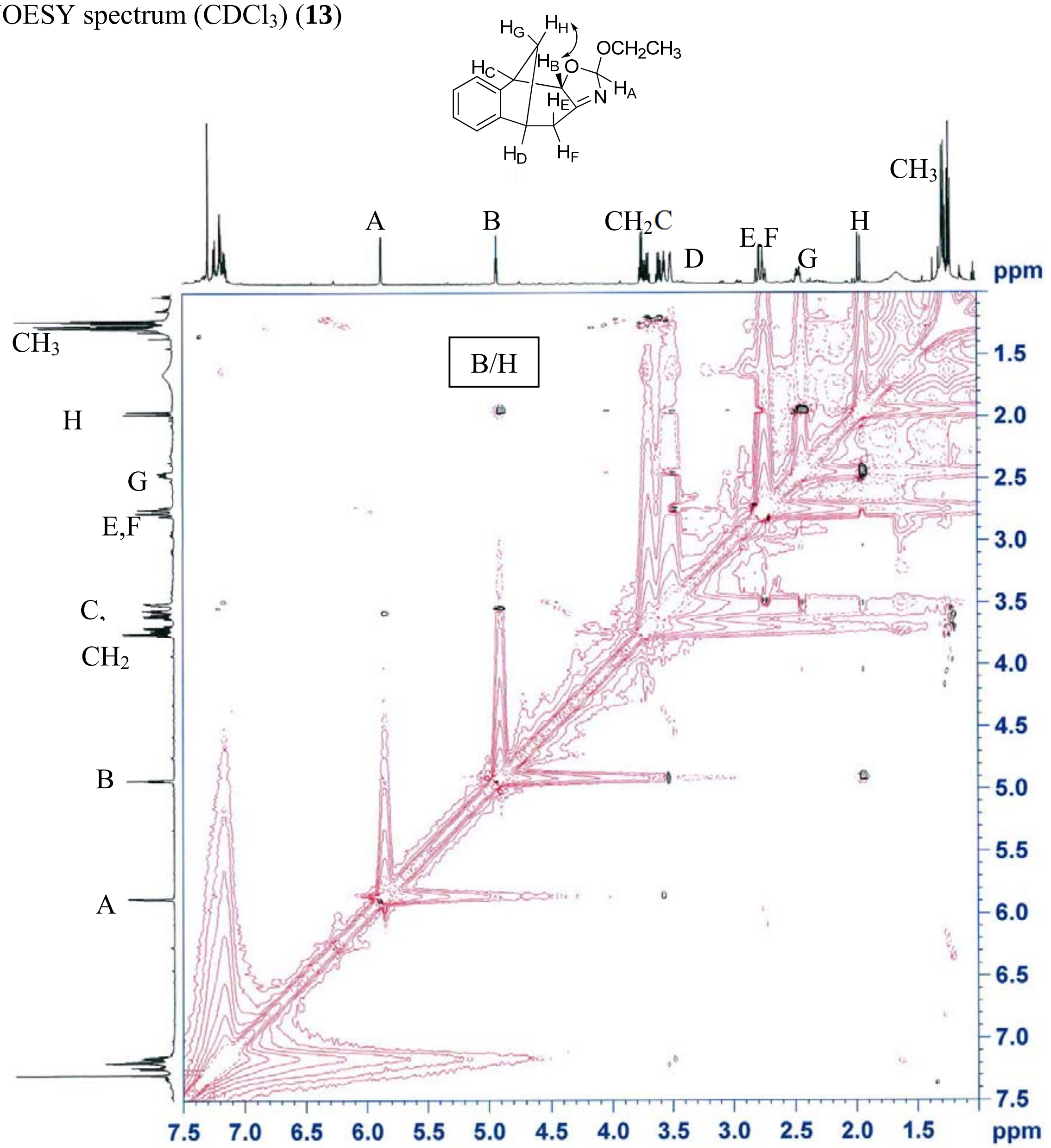

***rel*-(2*S*)-4-Methoxy(7-$^2H_1$)-3-oxa-5-azatetracyclo[6.6.1.0^{2,6}.0^{9,14}]pentadeca-5,9,11,13-tetraen (compound *rel*-(2*S*)-14)**

^1H NMR spectrum (600 MHz, $CDCl_3$) (**14**)

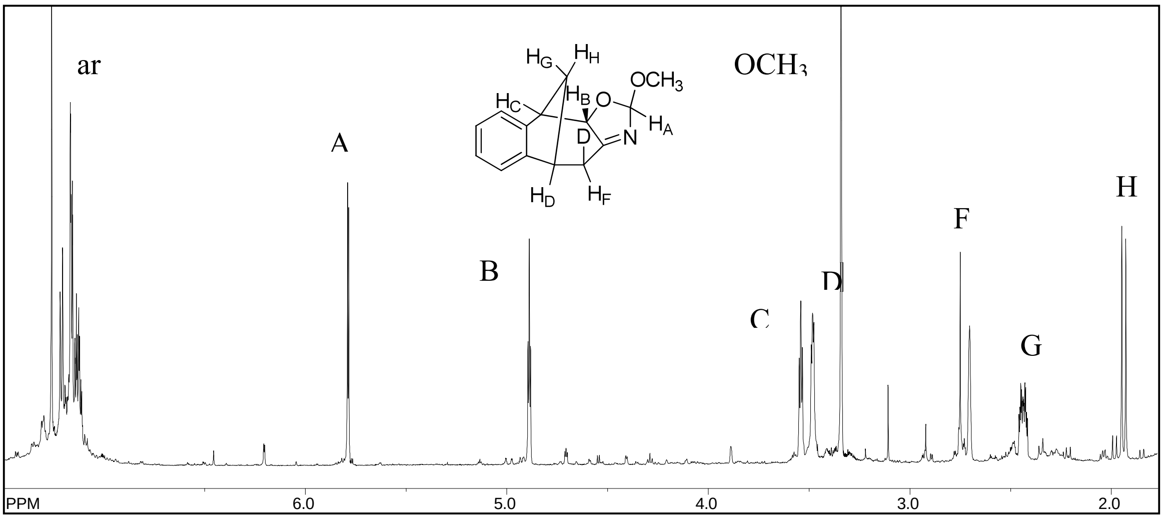

^{13}C NMR spectrum (150 MHz, 3s, 9d, 1t, 1q) ($CDCl_3$) (**14**)

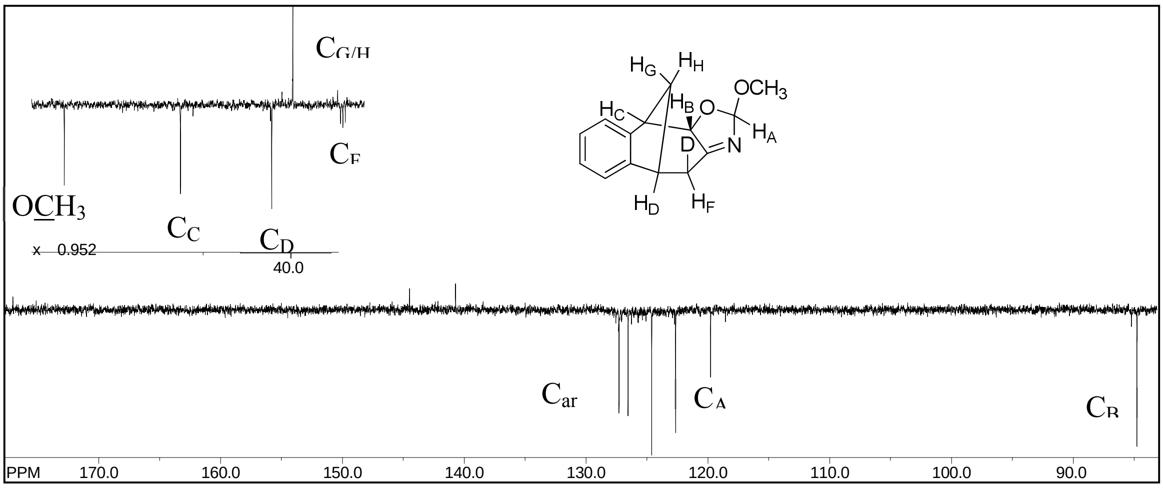

^{1}H NMR entire range spectrum (600 MHz, $CDCl_3$) (**14**)

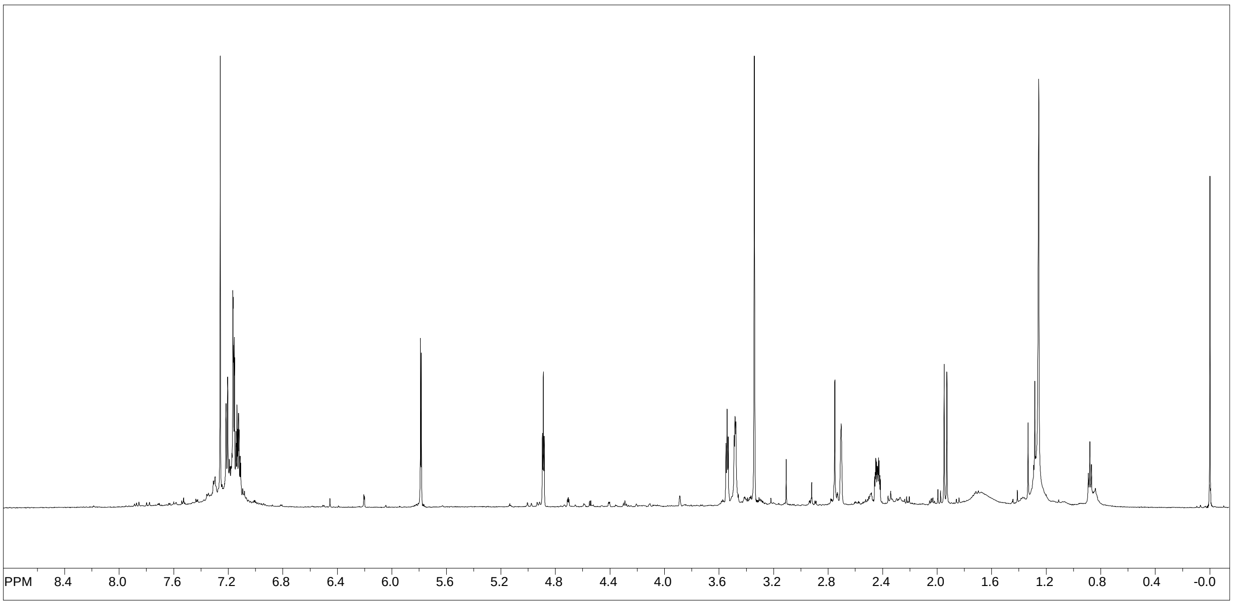

^{13}C NMR entire range spectrum (150 MHz, 3s, 9d, 1t, 1q) ($CDCl_3$) (**14**)

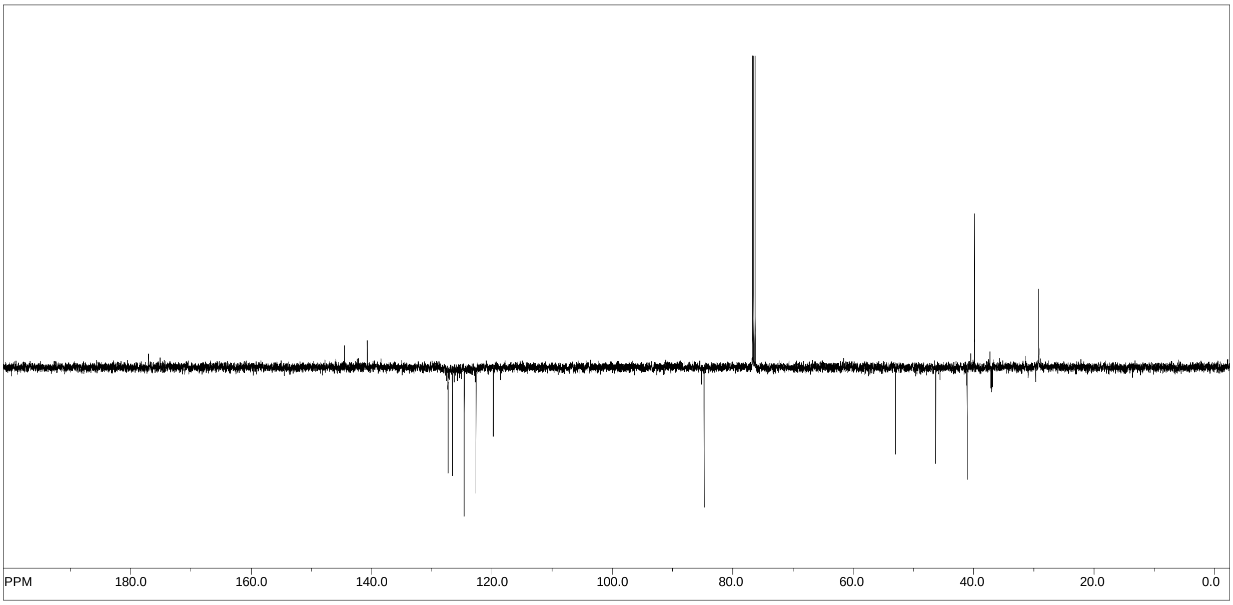

COSY spectrum ($CDCl_3$) (**14**)

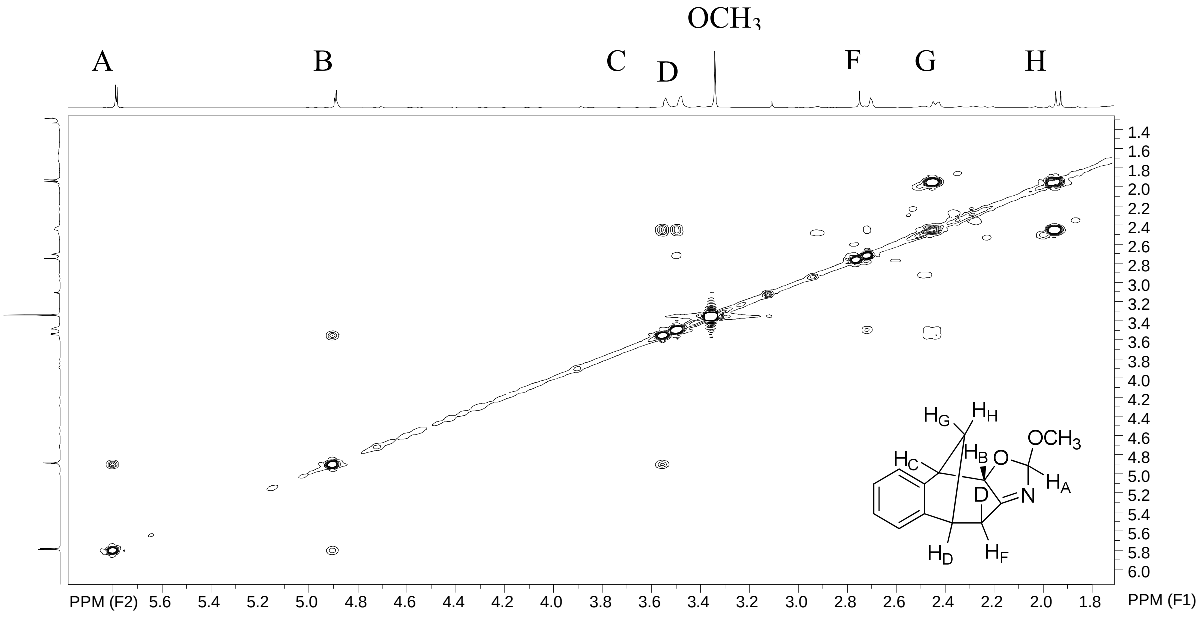

HSQC spectra of ($CDCl_3$) (**14**)

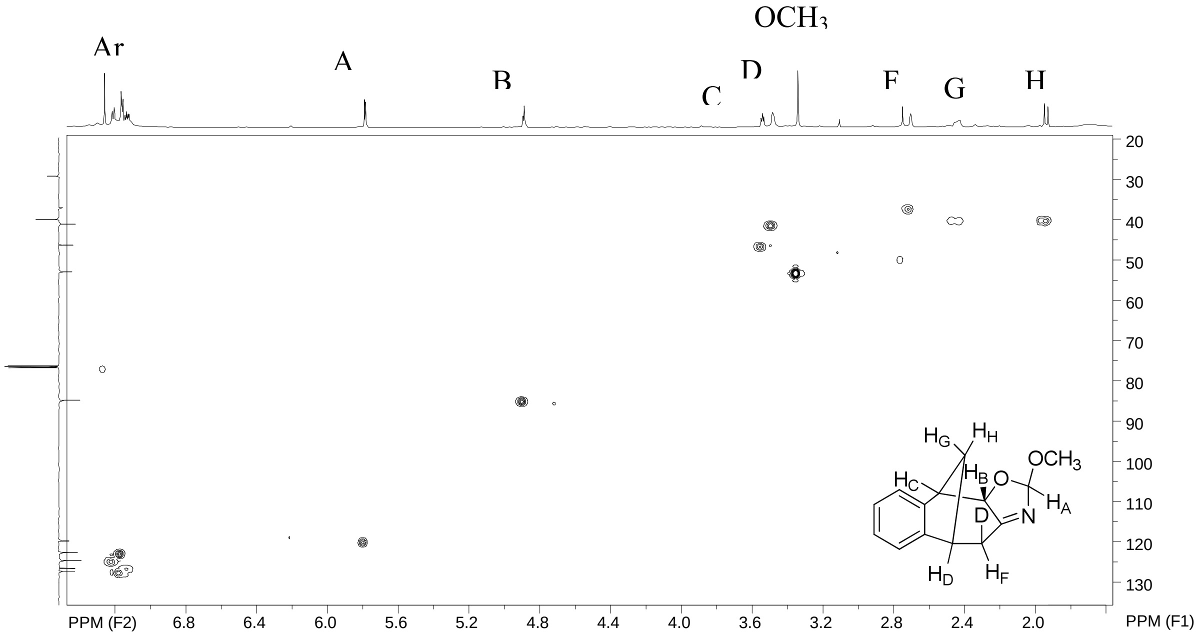

NOESY spectrum (CDCl$_3$) (**14**)

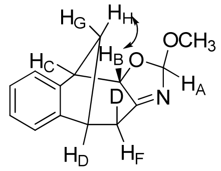

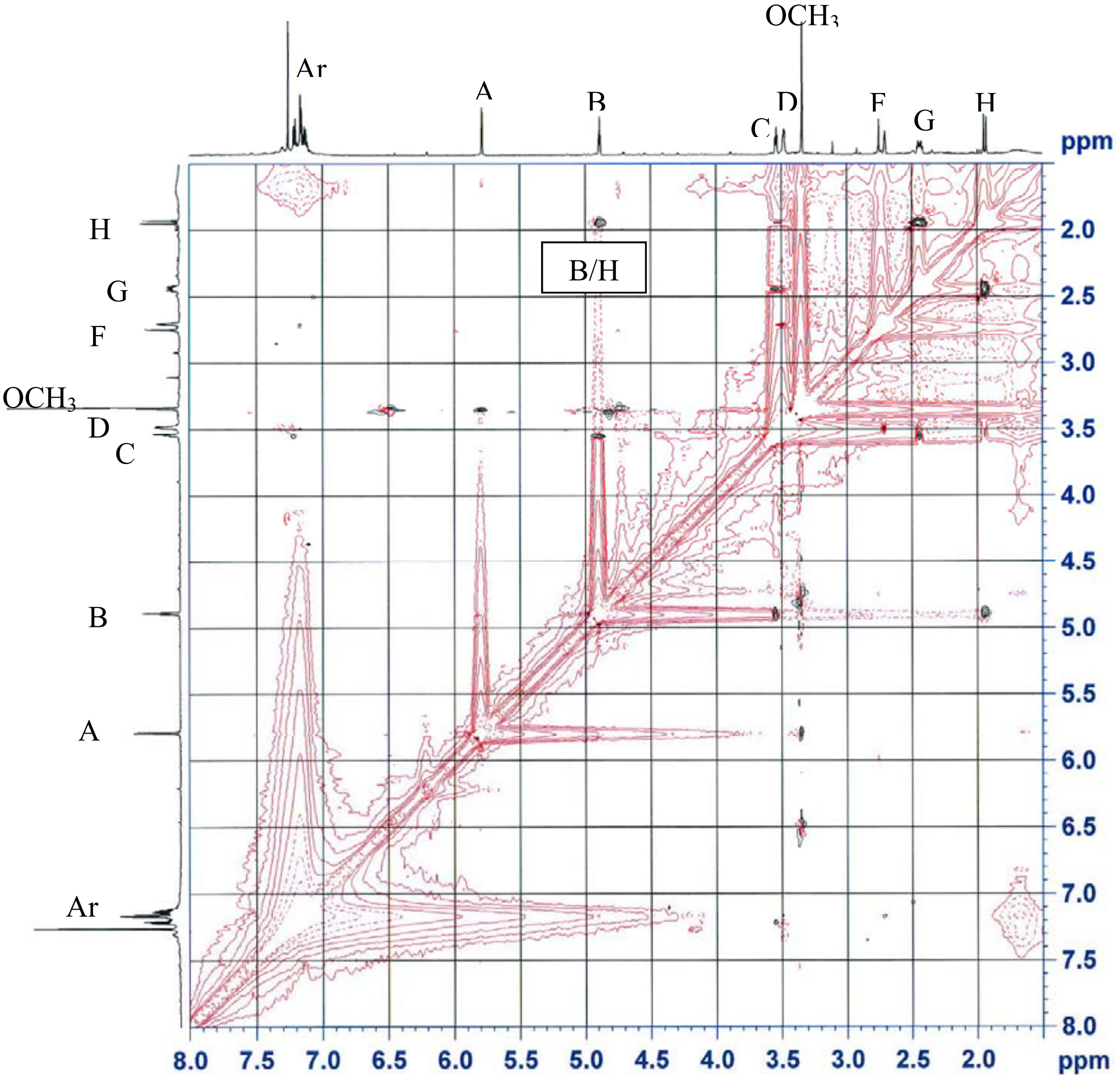

Supplement: File 1 — Experimental part, NMR and IR spectra. [file Beilstein_J_Org_Chem-10-2222-s001.pdf]
